# A half-center oscillator regulates sleep pressure

**DOI:** 10.1038/s41593-026-02431-9

**Published:** 2026-10-15

**Authors:** Peter S. Hasenhuetl, Raffaele Sarnataro, Eleftheria Vrontou, H. Olof Rorsman, Clifford B. Talbot, Ruth Brain, Gero Miesenböck

**Affiliations:** Centre for Neural Circuits and Behaviour, University of Oxford, Oxford, UK

**Keywords:** Sleep, Slow-wave sleep, Sleep deprivation, Cellular neuroscience, Synaptic transmission

## Abstract

Changes in oscillatory neural dynamics reflect—but may not necessarily determine—transitions between sleep stages and variations in sleep pressure. Here we show that sleep-control neurons innervating the dorsal fan-shaped body of *Drosophila* (dFBNs) produce slow-wave activity (SWA) that is causally linked to sleep. The dFBN ensemble contains bistable cells whose membrane voltages oscillate between hyperpolarized DOWN and depolarized UP states. The oscillations rely on interhemispheric competition of two half-centers connected by inhibitory glutamatergic synapses. dFBN-restricted interference with glutamate release disrupts SWA and baseline as well as rebound sleep, whereas the optogenetic replay of SWA induces sleep. dFBNs generate SWA throughout the sleep–wake cycle (despite mutual antagonism with arousing dopaminergic neurons) and adjust its power to sleep need via sleep history-dependent increases in excitability and homeostatic synaptic depression. The oscillatory format permits a continuous encoding of sleep pressure but requires mechanisms that convert the amplitude-modulated signal into binary sleep–wake states.

## Abstract (web summary)

Hasenhuetl et al. show that neurons innervating the dorsal fan-shaped body of *Drosophila* form a half-center oscillator, a circuit motif typically found in central pattern generators for locomotion. Here, this circuit generates slow-wave activity for the regulation of sleep.

## Main

The dynamics of neurons responsible for the homeostatic regulation of sleep, like those of all feedback controllers, are coupled to the dynamics of the controlled object: the outputs of one system serve as inputs to the other. Studies in *Drosophila* have begun to paint a detailed picture of how sleep-inducing neurons sense changes in the physiology of the sleeping and waking organism. Sleep-control neurons^1,2^ with projections to the dorsal layers of the fan-shaped body (dFBNs) estimate sleep pressure by monitoring the flow of electrons through their own mitochondria^3,4^. Sleep loss creates an imbalance between electron supply and ATP demand^4^ that diverts electrons from the respiratory chain into uncontrolled side reactions with molecular O_2_, producing reactive oxygen species^3^, which fragment the polyunsaturated fatty acyl (PUFA) chains of membrane lipids into short- or medium-chain carbonyls^5^. dFBNs count the release of PUFA-derived carbonyls (and transduce this signal into sleep) in a process that involves an allosteric dialog between the voltage-gated potassium channel Shaker—a critical determinant of dFBN activity^6^—and its carbonyl-reactive β-subunit Hyperkinetic^3,5,7^.

These insights were gained in experiments that clamped certain variables in order to isolate others. For example, to determine how sleep history^2^ or mitochondrial dynamics^4^ alter the current–spike frequency function of dFBNs, the neurons were driven with fixed current patterns; to resolve how cellular redox chemistry^3^, lipid peroxidation products^5^ or arousing dopamine^6^ modulate particular ion channels, the membrane potential of dFBNs was stepped between fixed voltages. How the unclamped dFBN population responds to changes in sleep pressure, and how these responses alter the sleep–wake state of the organism, therefore remains unknown.

## Results

### dFBN slow oscillations

**Fig. 1:**
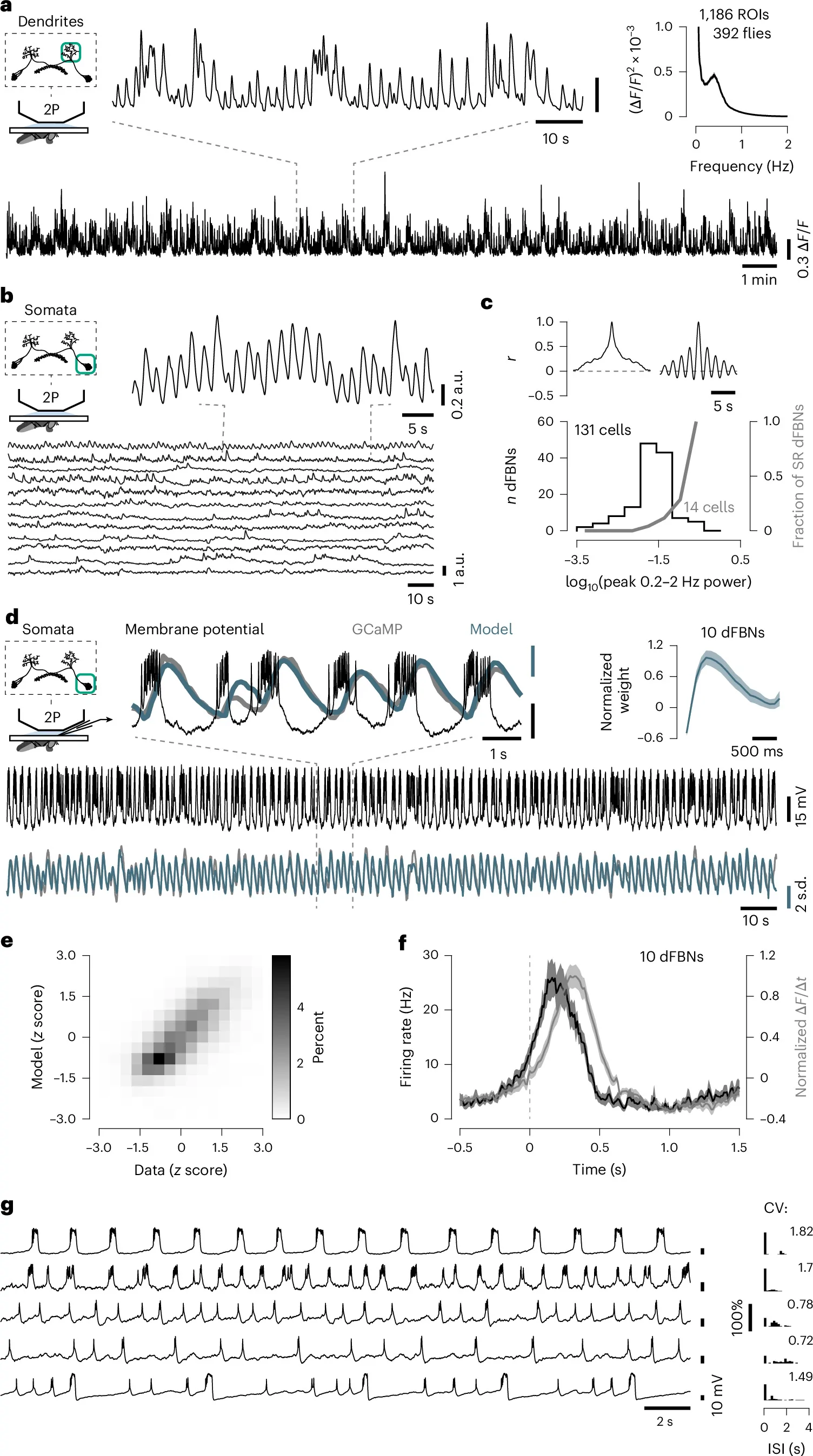
A subset of dFBNs generate SWA. **a**, Imaging of dFBN dendrites. The example shows 100 s of a GCaMP trace spanning ~22 min (bottom) at expanded *x* and *y* scales on top. Top *y*-axis scale bar, 0.3 Δ*F*/*F*. Top right: power spectrum. **b**, Simultaneous imaging of 12 dFBN somata in one hemisphere (intensity-normalized fluorescence). A portion of the uppermost trace is shown at expanded *x* and *y* scales on top. **c**, Top: GCaMP fluorescence autocorrelograms of the uppermost (right) and lowermost (left) dFBNs in **b**. Bottom: log-scaled distributions of 0.2–2 Hz power among 131 dFBNs in 17 hemispheres of 13 flies (black) and fraction of dFBNs with a slow rhythm (SR dFBNs) in each bin (gray). Prominent periodicity in the autocorrelogram with a wide amplitude swing during the first period was diagnostic of SR dFBNs. **d**, Simultaneous imaging and patch-clamp recording from dFBN somata. Black, gray and blue traces represent recorded voltages and *z*-score-normalized measured and predicted GCaMP traces, respectively, shown at expanded *x* and *y* scales on top. Lower end of voltage scale bar, –45 mV. Top right: normalized GCaMP impulse response. **e**, Two-dimensional histogram of measured versus predicted GCaMP signals, color coded according to the key on the right. *R*^2^ = 0.54 ± 0.21 (mean ± s.d.). **f**, Firing rate (30-ms moving average, black) aligned to GCaMP transient onset, defined as the zero crossing of the slope function of the normalized fluorescence trace (gray). **g**, Example whole-cell recordings from spontaneously spiking dFBNs. The coefficient of variation (CV) of the interspike interval (ISI) differs between tonic (<1), bursting (>1) and random (~1) activity. Data are means ± s.e.m. For imaging details, see Supplementary Table 1.

**Extended Data Fig. 1:**
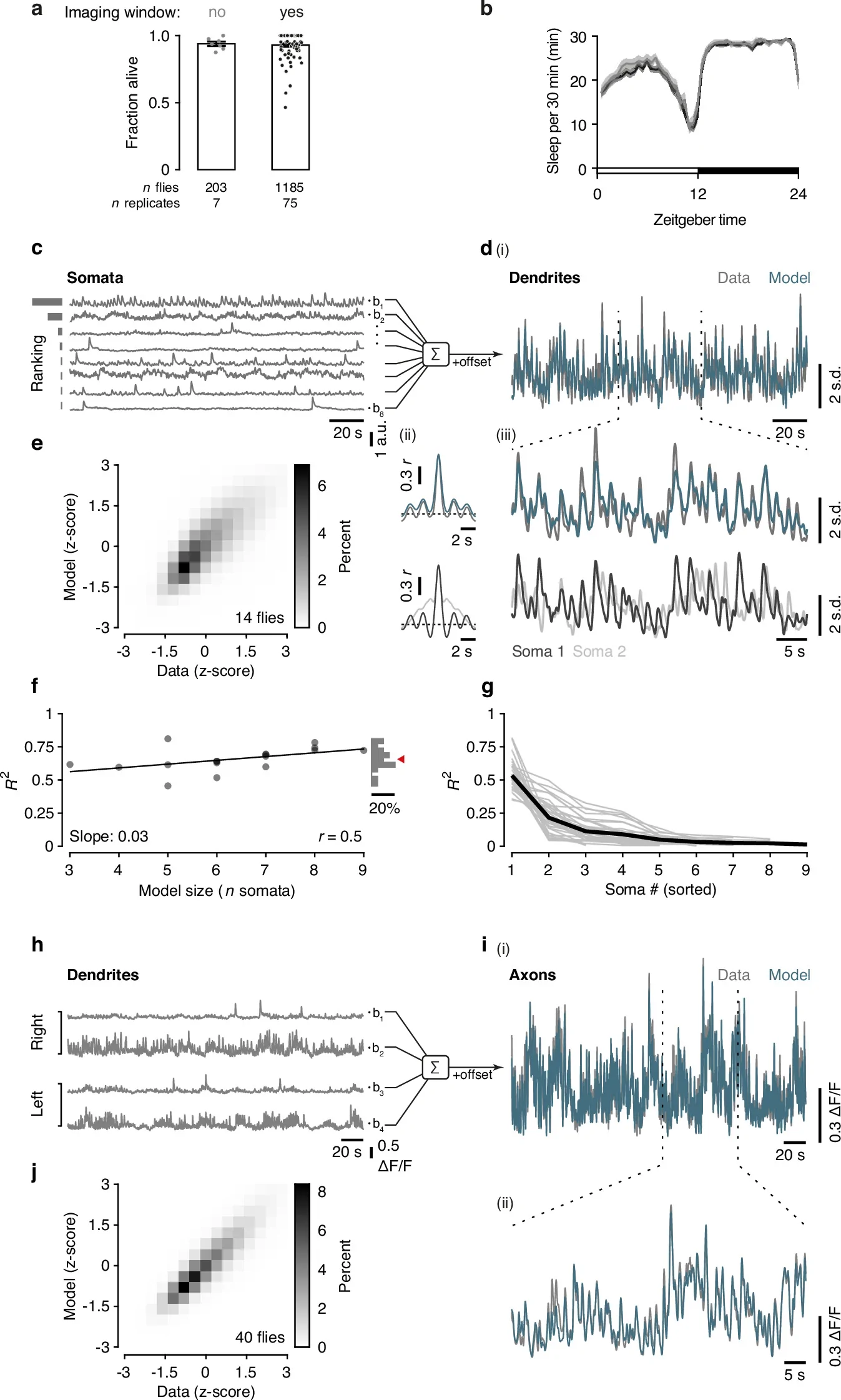
Implantation of chronic imaging windows and simultaneous imaging of dFBN somata and dendrites, or of dendrites and axons. **a**, Implantation of a chronic optical imaging window does not reduce the survival of operated flies at 24–48 h after surgery (black) relative to controls (gray) (*P* = 0.5419, two-sided Mann-Whitney test). **b**, Sleep profiles of flies implanted with a chronic imaging window (black, *n* = 74) versus controls (gray, *n* = 37). Two-way repeated-measures ANOVA fails to detect significant effects on sleep (effect of window implantation: *P* = 0.2355, time × window implantation interaction: *P* = 0.9890). Data are means ± s.e.m. **c**, **d**, Example GCaMP traces of dFBN somata (**c**) and dendrites (**d**). A linear model using the somatic signals as predictor variables (blue) accurately describes the dendritic GCaMP trace (**d**(i), gray), which is dominated by dFBNs with a slow rhythm (ii) and shown at an expanded time scale below (iii). Cells in **c** are ranked by the proportion of variation in dendritic fluorescence predicted by somatic fluorescence (see **g**). **e**, Two-dimensional histogram of measured versus predicted dendritic GCaMP signals (*n* = 14 flies, 17 separately recorded hemispheres, 35 dendritic ROIs). Data in the histogram were *z*-score-normalized for display but not for fitting and are color coded according to the key on the right. **f**, Model performance (*R*^*2*^) as a function of the number of simultaneously imaged dFBNs. The vertical histogram summarizes the dataset; red arrowhead, mean *R*^*2*^. **g**, Proportion of variation in dendritic fluorescence predicted by somatic fluorescence, in decreasing order of *R*^*2*^. **h**, **i**, Example GCaMP traces of dFBN dendrites (**h**) and axons (**i**). A linear model using the dendritic signals as predictor variables (blue) accurately describes the axonal GCaMP trace (**h**(i), gray, shown at an expanded time scale below (ii)). **j**, Two-dimensional histogram of measured versus predicted axonal GCaMP signals. Data in the histogram were *z*-score-normalized for display but not for fitting and are color coded according to the key on the right. For imaging details see Supplementary Table 1. For statistical details see Supplementary Table 3.

To visualize unperturbed dFBN ensemble activity in vivo, we implanted flies expressing the genetically encoded Ca^2+^-sensor GCaMP6f (GCaMP in brief) under the control of *R23E10-GAL4* with translucent windows (Methods; Extended Data Fig. 1a,b) and imaged the dendritic tufts of dFBNs by two-photon microscopy. Dendritic fluorescence displayed prominent Ca^2+^ transients, which repeated rhythmically at 0.2–1 Hz (Fig. 1a and Supplementary Video 1). In analogy to the nomenclature in mammals^8^, we refer to activity in the frequency band of 0.2–2 Hz as slow-wave activity (SWA).

The regularity of GCaMP transients across the dataset in Fig. 1a raises the question of whether the entire dFBN population is engaged in synchronous oscillations or whether a subset of rhythmic cells dominates dendritic fluorescence, which represents a weighted sum of contributions from all dFBN processes in the imaged region. To distinguish these possibilities, we recorded simultaneous GCaMP signals from a median of 8 cell bodies (range: 3–12) in the same hemisphere (Fig. 1b and Supplementary Video 2) and found that time series of somatic fluorescence were, on average, uncorrelated (Pearson *r* = 0.05 ± 0.15, mean ± s.d). Different dFBNs concentrated variable amounts of power in the 0.2–2 Hz band, giving rise to a heavily skewed distribution (Fig. 1c, note the logarithmic scale). At the upper extreme of this continuum were neurons with pacemaker-like Ca^2+^ oscillations that resembled those in dendrites (Fig. 1b,c and Extended Data Fig. 1c–g). A handful of neurons could therefore account for much of the variance of the dendritic compound signal (mean *R*^2^ = 0.66, range: 0.46–0.81; Extended Data Fig. 1c–g), which in turn correlated tightly with axonal fluorescence (Extended Data Fig. 1h–j).

To characterize the electrical events underlying GCaMP transients in slowly oscillating dFBNs, we performed simultaneous whole-cell patch-clamp and two-photon Ca^2+^-imaging experiments in vivo. The superimposition of voltage and GCaMP traces in Fig. 1d shows that Ca^2+^ transients coincided with large, sustained depolarizations generating high-frequency action potential bursts. These UP states alternated with DOWN states of somewhat longer average duration during which the neurons were hyperpolarized and stopped firing. For a quantitative analysis, we estimated the GCaMP impulse response from these recordings and used it successfully to predict fluorescence signals from the action potential record (Fig. 1d,e); a spike frequency histogram aligned to the onset of GCaMP transients peaked ~120 ms before the maximal fluorescence change (Fig. 1f).

The metronome-like alternations of UP and DOWN states underpinning the dendritic Ca^2+^ signal represent only one of several forms of temporally structured activity in the dFBN population. Whole-cell recordings showed that some neurons released rhythmic bursts of action potentials at faster repetition rates than 0.5 Hz, others fired tonically at regular interspike intervals, and still others produced repeating sequences of spikes and bursts (Fig. 1g). A few, but by no means all, of these activity patterns could also be discerned in the somatic Ca^2+^ traces of individual dFBNs (Fig. 1b and Extended Data Fig. 1c).

### A half-center oscillator generates SWA

Rhythmic neural activity is typically a product of cell-intrinsic oscillators, such as hyperpolarization-activated cation channels^9^, or oscillatory circuits, which abound in the neural control of movement^10–13^ but have also been proposed to regulate sleep cycle time^14,15^, if not sleep itself. In a prototypical oscillatory circuit, exemplified by the two-neuron swimming system of *Clione*^11^ or the myriad central pattern generators that populate invertebrate ganglia or the vertebrate brain stem, mutual inhibition between two half-centers controlling opponent sets of muscles produces self-perpetuating cyclic movement^10–13^.

**Fig. 2:**
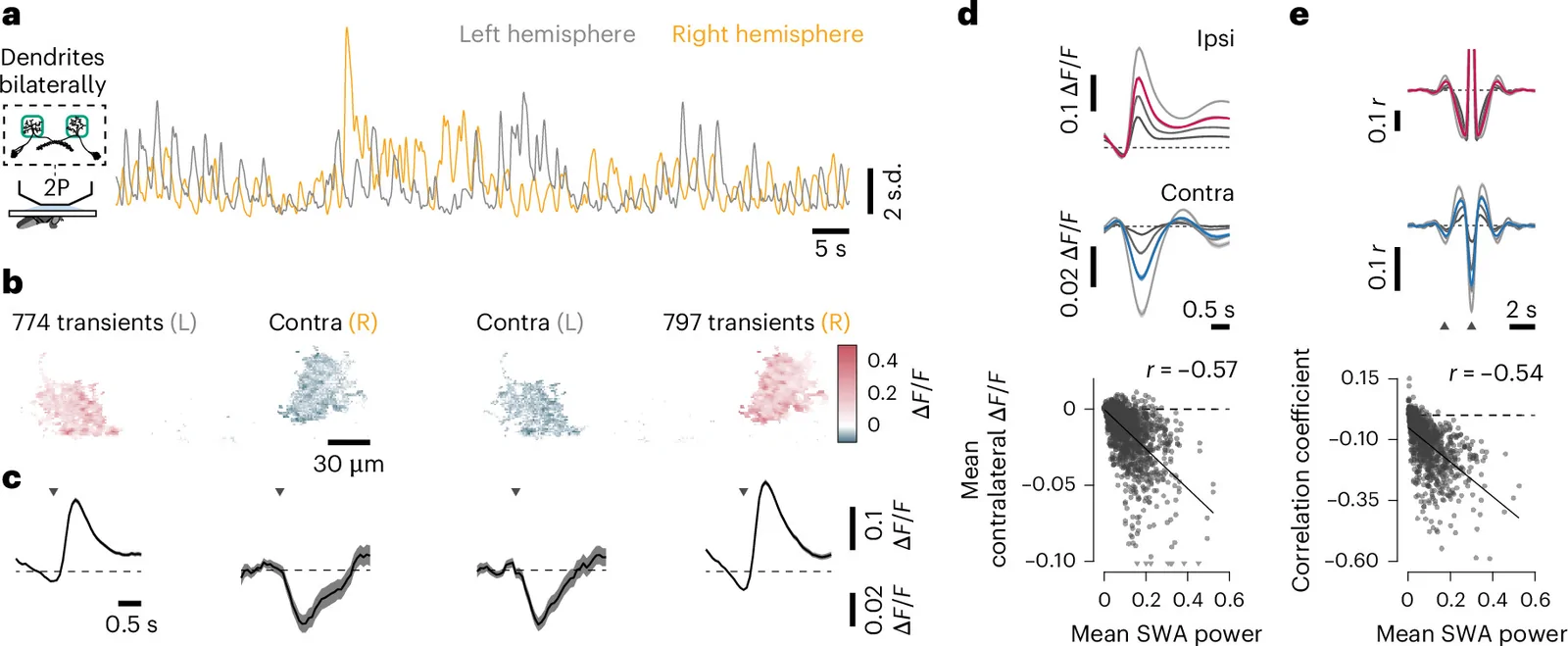
dFBNs form a half-center oscillator. **a**, Simultaneous imaging of dFBN dendrites in both hemispheres. The example shows 100 s of *z*-score-normalized GCaMP traces spanning ~22 min. **b**,**c**, Ipsi- (red) and contralateral (blue) *z*-score-normalized fluorescence changes (**b**) and GCaMP traces (**c**) during transients in the left (L) and right (R) hemispheres (same fly as in **a**). Images in **b** depict mean Δ*F*/*F* in 10 frames (~700 ms) after transient onset versus the preceding 15 frames; the images are thresholded for display and pseudocolored according to the key on the right. Ipsi- and contralateral GCaMP traces in **c** are plotted at different Δ*F*/*F* scales, arrowheads mark the onset of ipsilateral transients and dashed horizontal lines indicate Δ*F*/*F* = 0. **d**,**e**, Transient-aligned average ipsi- (top) and contralateral (center) GCaMP traces (**d**) and their auto- and crosscorrelograms (**e**) in the full dataset (*n* = 326 flies). Shades of gray from dark to light show increasing quartiles of SWA power in both hemispheres, and red and blue traces represent ipsi- and contralateral population averages, respectively. Bottom: linear regression of mean contralateral Δ*F*/*F* (**d**) and interhemispheric correlation coefficients (**e**) versus SWA power in both hemispheres. Ten data points exceeding the *y*-axis limit are plotted as triangles at the bottom of the graph in **d**; the correlation coefficient is based on the actual values. Shades of gray from dark to light show increasing quartiles of SWA power and colored traces represent population averages. Correlograms are clipped near autocorrelation peaks and arrowheads indicate one period. Data are means ± s.e.m. For imaging details see Supplementary Table 1.

**Extended Data Fig. 2:**
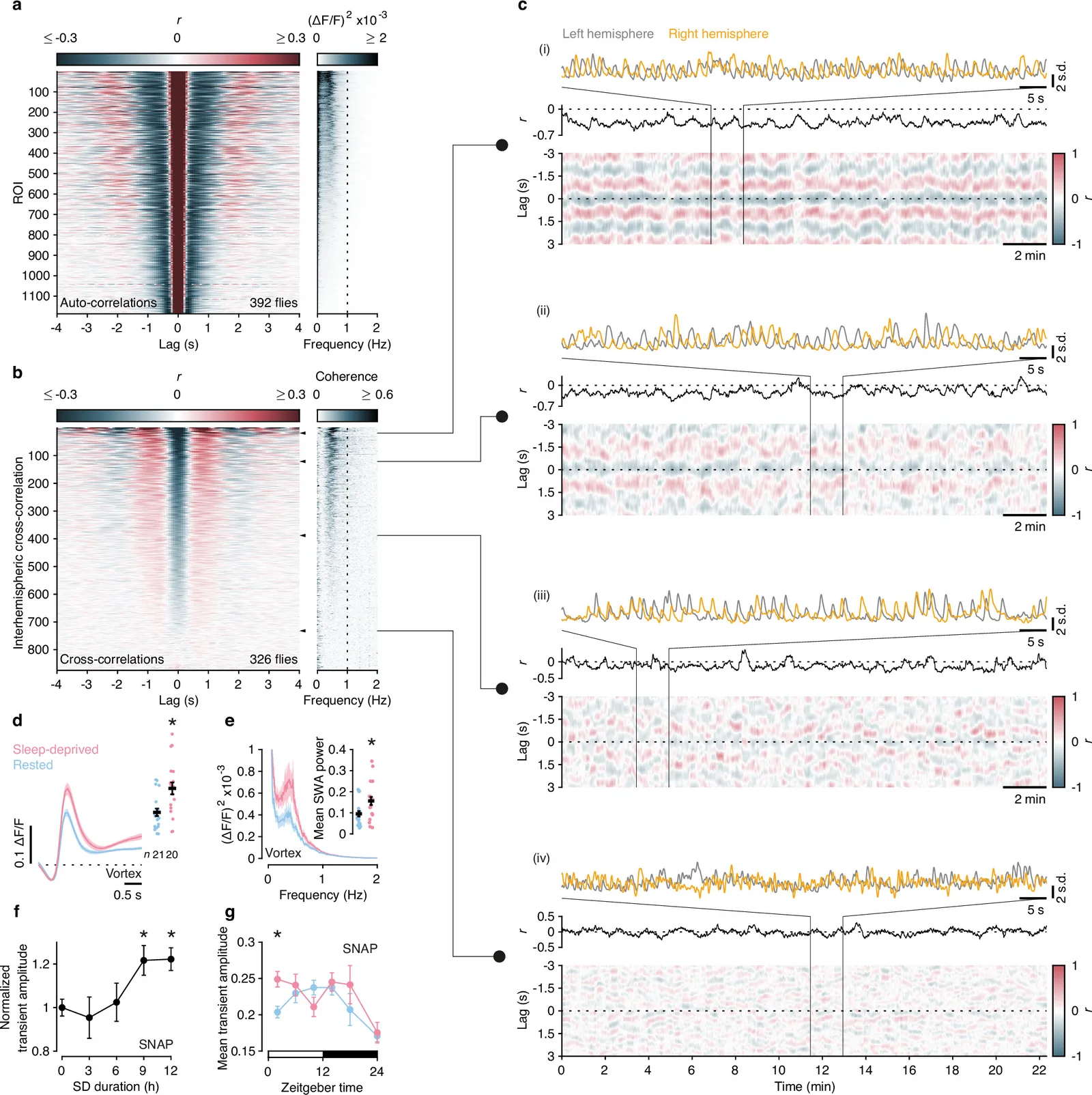
dFBN ensemble dynamics as a function of sleep need and zeitgeber time. **a**, Autocorrelograms (left) and power spectra (right) of GCaMP traces in all dendritic regions of interest (ROIs), sorted (in descending order) by SWA power and color coded according to the keys on top. **b**, Crosscorrelograms (left) and coherence spectra (right) of dendritic GCaMP traces during imaging of both hemispheres, sorted (in descending order) by the average interhemispheric anticorrelation at a lag of 0 ± 300 ms and color coded according to the keys on top. **c**, Time-varying crosscorrelograms (sliding 20-s windows, bottom panels) and correlation coefficients (center panels) of dendritic GCaMP traces in the left and right hemispheres at four levels of coherence. Top panels, left and right dendritic GCaMP traces at an expanded time scale. **d**, **e**, Sleep deprivation by vortex increases the average GCaMP transient (**d**, left), its peak amplitude (**d**, right, *P* = 0.0013, two-sided *t*-test) and SWA power (**e**, *P* = 0.0122, two-sided *t*-test). **f**, GCaMP transient amplitudes (normalized to unperturbed controls) at ZT 0–4 as a function of the duration of prior sleep deprivation (*P* = 0.0045, Kruskal-Wallis ANOVA). *n* at SD durations of 0, 3, 6, 9 and 12 h: 55, 14, 12, 47, 41 (same data at 0- and 12-h time points as in **g** and Fig. 6b). **g**, Mean amplitude of GCaMP transients during the course of a day in rested (blue) and sleep-deprived (red) flies (effect of sleep history: *P* < 0.0001, Kruskal-Wallis ANOVA; effect of ZT on rested flies: *P* = 0.0011, Kruskal-Wallis ANOVA). *n* of rested and sleep-deprived flies, respectively, at the given ZT were 0–4: 55, 41; 4–8: 32, 14; 8–12: 28, 14; 12–16: 34, 15; 16–23: 13, 9; 23–4: 9, 11. Same dataset as in Fig. 6e. Data are means ± s.e.m.; asterisks indicate significant differences (*P* < 0.05) in planned pairwise comparisons. For imaging details see Supplementary Table 1. For statistical details see Supplementary Table 3.

Analyses of dFBN activity, along with connectomic evidence^16,17^, indicate that two mutually inhibitory half-centers contribute to the generation of SWA. Simultaneous bilateral imaging showed that Ca^2+^ transients in dFBNs of the left and right hemispheres alternated out of phase (Fig. 2a and Supplementary Video 1), so that the occurrence of a fluorescence maximum in one hemisphere was associated, on average, with a minimum in the other (Fig. 2b,c). In keeping with the idea that direct inhibition from the contralateral hemisphere is the source of the negative fluorescence deflection, the GCaMP signal dipped deeper the higher was the mean SWA power in both hemispheres (Fig. 2d). As expected for two neuronal populations synchronized in antiphase, interhemispheric crosscorrelations returned minima at a lag of 0 s and a period of ~2 s, corresponding to an oscillation frequency of 0.5 Hz (Fig. 2e and Extended Data Fig. 2a–c).

**Extended Data Fig. 3:**
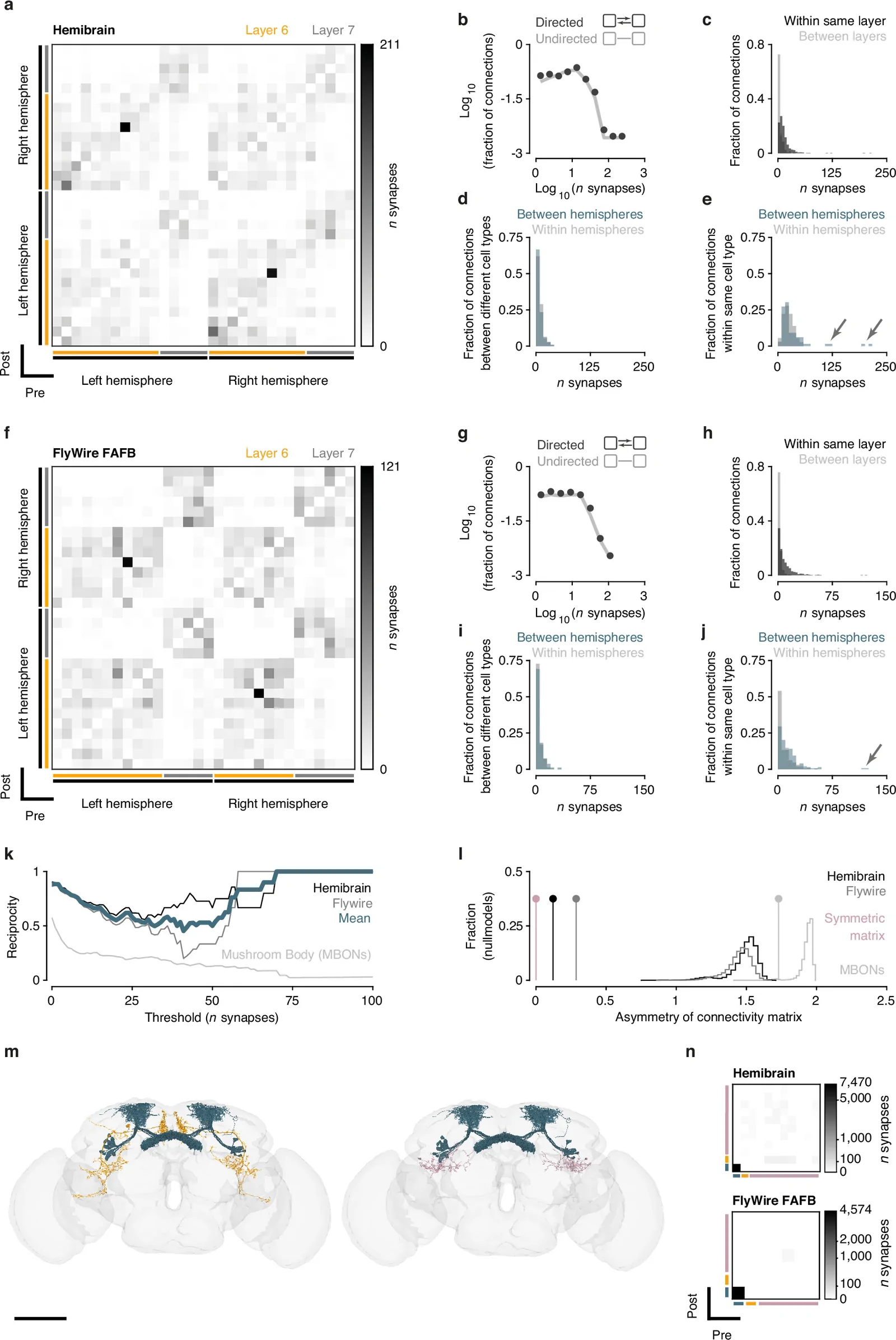
Connectomic support for a half-center oscillator. **a**, Connectivity matrix of dFBNs in the hemibrain:v1.2.1 dataset (*n* = 31 dFBNs of both hemispheres, projecting to layers 6 and 7). Presynaptic neurons in rows, postsynaptic neurons in columns. **b**, Number of synapses per inter-dFBN connection. Conversion of directed (dark gray) to undirected edges (light gray) leaves the histogram unchanged. **c**, Inter-dFBN connections within (black) and between (gray) layers. dFBNs interconnect preferentially within their respective layers (*P* < 0.0001, two-sided Mann-Whitney test). **d**, **e**, Inter- (green) and intra-hemispheric connections (light gray) between different (**d**) and the same (**e**) hemibrain dFBN types. The average number of synapses between the same dFBN types (29.96) exceeds the average number of synapses between different types (7.62) (*P* < 0.0001, two-sided Mann-Whitney test). **f**, Connectivity matrix of dFBNs in the FlyWire FAFB v783 dataset (*n* = 30 dFBNs of both hemispheres, projecting to layers 6 and 7). Presynaptic neurons in rows, postsynaptic neurons in columns. **g**, Number of synapses per inter-dFBN connection. Conversion of directed (dark gray) to undirected edges (light gray) leaves the histogram unchanged. **h**, Inter-dFBN connections within (black) and between (gray) layers. dFBNs interconnect preferentially within their respective layers (*P* < 0.0001, two-sided Mann-Whitney test). **i**, **j**, Inter- (green) and intra-hemispheric connections (light gray) between different (**i**) and the same (**j**) hemibrain dFBN types. The average number of synapses between the same dFBN types (10.54) exceeds the average number of synapses between different types (4.15) (*P* < 0.0001, two-sided Mann-Whitney test). **k**, Reciprocity of inter-dFBN connections as a function of synapse number. Black, gray and blue traces are hemibrain, FlyWire and the average of both, respectively. Light gray trace, connectivity of mushroom body output neurons (MBONs; FlyWire dataset). **l**, Asymmetry of inter-dFBN connectivity matrices of hemibrain and FlyWire connectomes, compared to their null models (black and gray stems and histograms, respectively). Values close to the mean of the respective null models are a sign of asymmetric connectivity. Light gray stem and histogram, MBONs. Red stem, symmetric matrix. **m**, Skeleton reconstructions via FlyWire Codex of dFBNs (teal) and extraneous cells labelled by the *R23E10 ∩ VGlut-GAL4* driver: aMe24 (yellow, left) and 1–3 cells with arborizations in the posterior lateral protocerebrum, including PLP119 (pink, right). Scale bar, 100 μm. **n**, Connectivity matrices of all dFBNs (teal) and potential extraneous cells (aMe24 in yellow; neurons with arborizations in the posterior lateral protocerebrum in pink) in the hemibrain:v1.2.1 (top) and FlyWire FAFB v783 datasets (bottom). Presynaptic neurons in rows, postsynaptic neurons in columns. The number of synaptic connections is color coded according to the keys on the right and zero between dFBNs and all potential extraneous cells (aMe24, CL141, LHPV2c3, PLP119, PLP120, PLP129, PLP145, PLP149, PLP200, SLP361 in the hemibrain:v1.2.1 dataset; aMe24, CB3920, LHPV6k2, LHPV6o1, PLP119, PLP156 in the FlyWire FAFB v783 dataset). For statistical details see Supplementary Table 3.

The symmetry and strength of connections among dFBNs in the hemibrain^16^ and FlyWire^17^ connectomes meets the anatomical requirements of a half-center oscillator (Extended Data Fig. 3). Almost perfectly symmetric weighted connectivity matrices (Extended Data Fig. 3a,f), each exhibiting a correlation coefficient of 0.92 or 0.81, respectively, with its transpose and a weight distribution indistinguishable from an undirected graph (Extended Data Fig. 3b,g), link the members of the population. Although dFBN interconnectivity is generally dense (full dFBN network: 0.66–0.73; dFBNs within layer 6: 0.94–0.96; dFBNs within layer 7: 0.96–0.97) and reciprocal, regardless of synapse number, both connectomes favor connections within layers and between hemispheres and contain subsets of layer 6-projecting dFBNs^16^ with exceptionally strong connections with their contralateral counterparts (Extended Data Fig. 3a–j). We tentatively equate these neurons with dFBNs displaying a slow rhythm (Fig. 1c) but stress that a high degree of connection symmetry and reciprocity characterizes the dFBN population as a whole (Extended Data Fig. 3k,l).

**Fig. 3:**
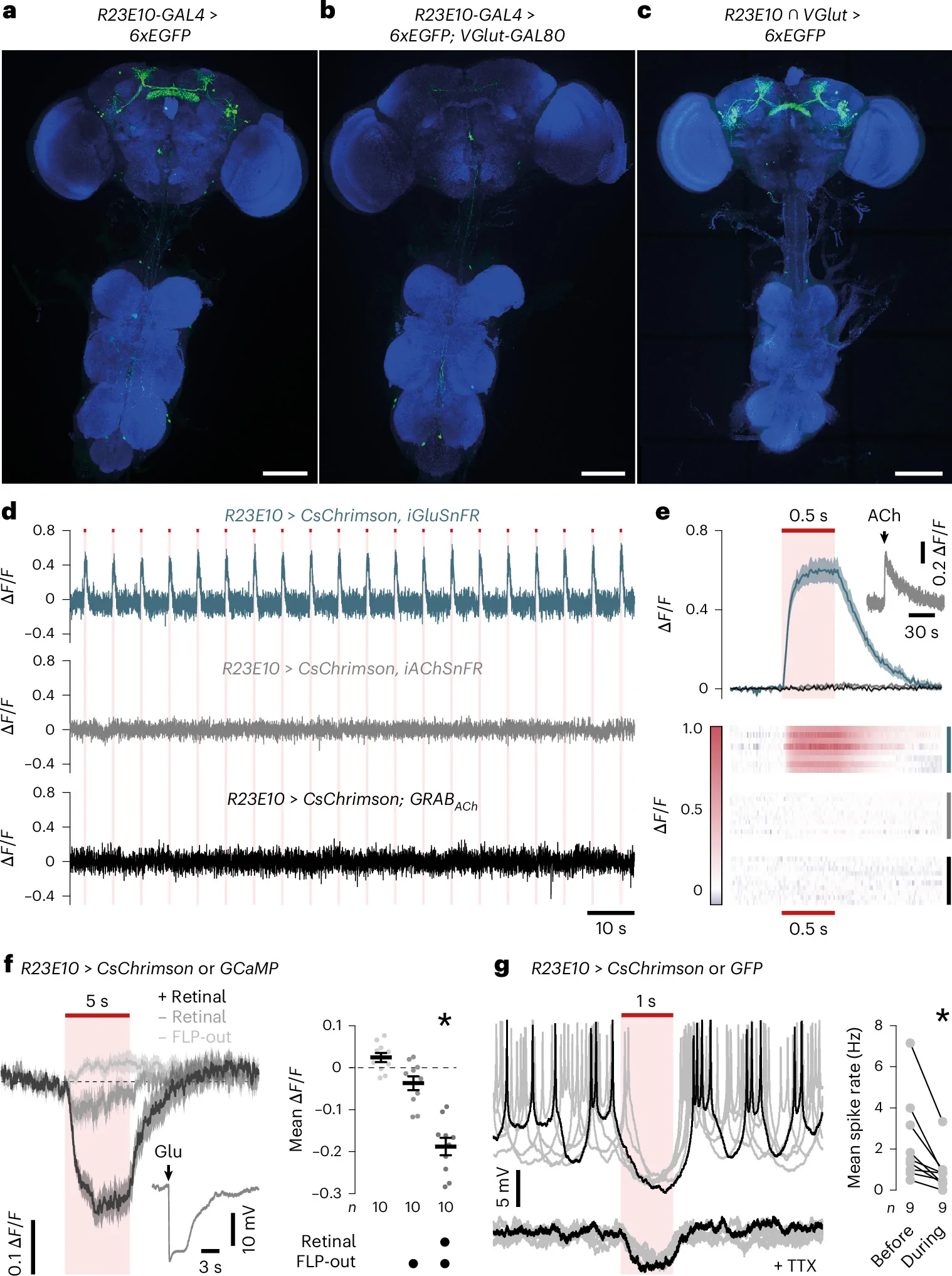
dFBNs are glutamatergic and mutually inhibitory. **a**,**b**, Maximum-intensity projections of the central nervous system of flies expressing 6xEGFP under the control of *R23E10-GAL4*, in the absence (**a**) or presence (**b**) of the transcriptional repressor *VGlut-GAL80*. **c**, Maximum-intensity projection of the central nervous system of a fly expressing 6xEGFP under the control of hemidrivers *R23E10-DBD* and *VGlut-p65AD* (*R23E10 ∩ VGlut-GAL4*). Green, 6xEGFP; blue, Bruchpilot (BRP). **d**,**e**, Optogenetic stimulation of dFBNs coexpressing CsChrimson and iGluSnFR (blue), iAChSnFR (gray) or GRAB_ACh_ (black) in the presence of 1 μM TTX (*n* = 8, 10 and 10 flies, respectively). Baseline-corrected example traces during 20 light pulses (**d**; vertical bars, 500 ms). Average stimulus-aligned, normalized fluorescence transients (**e**, top; inset, iAChSnFR after pressure ejection of acetylcholine) and stimulus-aligned averages of individual flies, color coded as in **d** (**e**, bottom). **f**,**g**, Imaging (**f**) and electrophysiological (**g**) demonstration of inhibition in dFBN mosaics expressing CsChrimson or a green fluorophore (GCaMP in **f**, mCD8::GFP in **g**) after FLP-mediated recombination (inset in **f**, membrane voltage after pressure ejection of glutamate onto dFBN dendrites). Somatic GCaMP fluorescence of CsChrimson-negative dFBNs declines during optogenetic stimulation of CsChrimson-positive dFBNs in the presence of retinal (**f**, *P* < 0.0001, ANOVA). Optogenetic stimulation of CsChrimson-positive dFBNs hyperpolarizes CsChrimson-negative dFBNs (overlaid voltage traces of six consecutive trials, one trial in black) and suppresses spiking (**g**, *P* = 0.0039, two-sided Wilcoxon test). Inter-dFBN inhibition persists in the presence of 1 µM TTX. Data are means ± s.e.m.; *n*, number of flies (**f**) or cells (**g**); asterisks indicate significant differences (*P* < 0.05) in planned pairwise comparisons. Scale bars, 100 μm. For imaging details see Supplementary Table 1. For statistical details see Supplementary Table 2.

**Extended Data Fig. 4:**
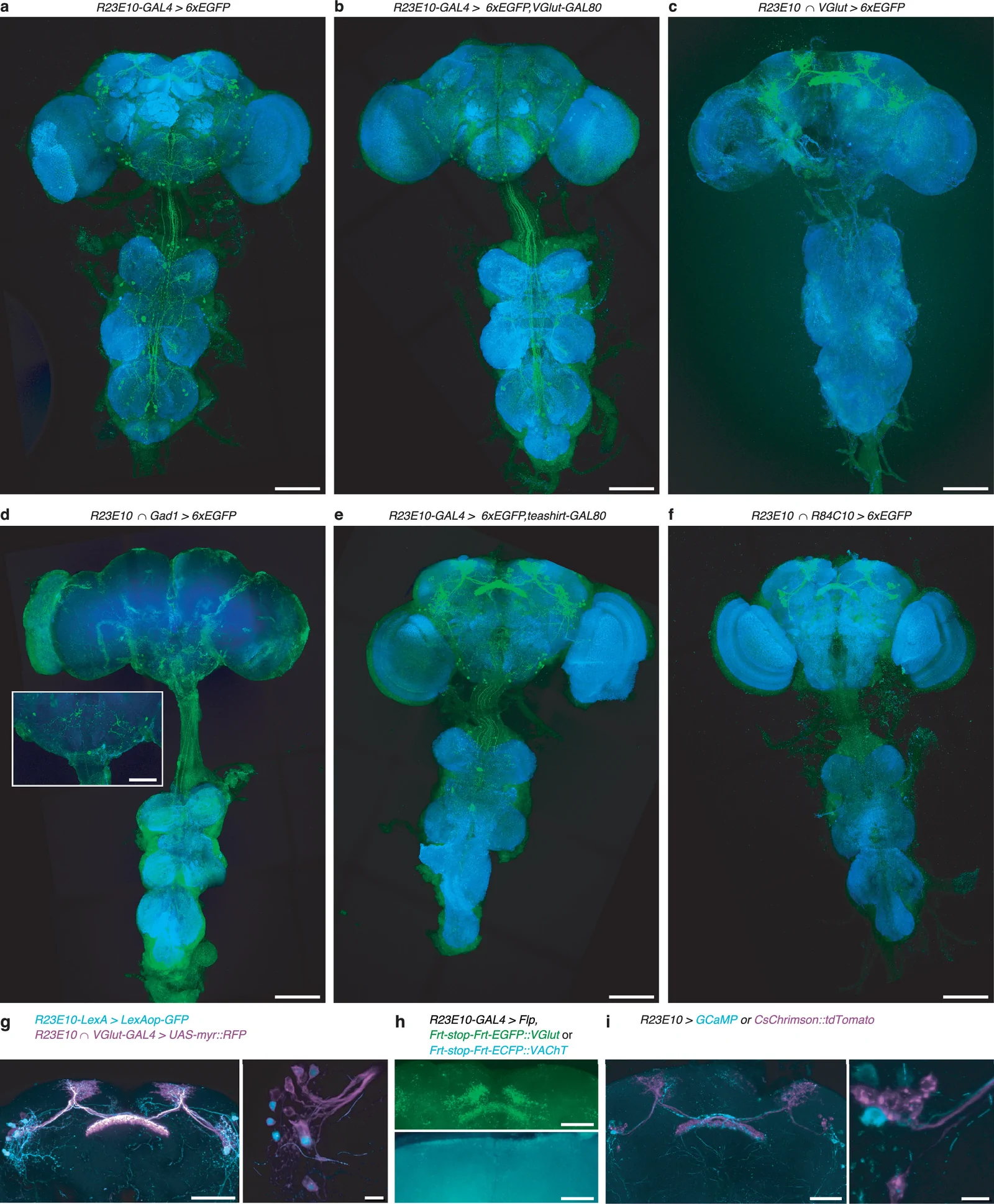
Genetic resolution of dFBNs. **a**–**f**, Saturated maximum-intensity projections of the CNS of flies expressing 6xEGFP under the control of *R23E10-GAL4*, in the absence (**a**) or presence of the transcriptional repressors *VGlut-GAL80* (**b**) or *teashirt-GAL80* (**e**), or under the control of hemidrivers *R23E10-DBD* and *VGlut-p65AD* (**c**, *R23E10* ∩ *VGlut-GAL4*), *R23E10-DBD* and *Gad1-p65AD* (**d**, *R23E10* ∩ *Gad1-GAL4*) or *R23E10-DBD* and *R84C10-p65AD* (**f**, *R23E10* ∩ *R84C10-GAL4)*. The inset in **d** shows a magnified view of the suboesophageal zone. Green, 6xEGFP; blue, BRP. Differences in the visibility of grid lines reflect differences in the acquisition settings of composite scans. **g**, Maximum-intensity projections of fly brains expressing GFP under the control of *R23E10-LexA* and myr::RFP under the control of *R23E10* ∩ *VGlut-GAL4* (left, whole midbrain; right, dFBN somata). *R23E10* ∩ *VGlut-GAL4* captures all dFBNs labelled by *R23E10-LexA*. Additional myr::RFP-positive dFBNs are included in the *R23E10-GAL4* pattern but missing from *R23E10-LexA*. **h**, Maximum-intensity projections of the dorsal fan-shaped bodies of fly brains expressing EGFP::VGlut (top) or ECFP::VAChT fusion proteins (bottom, saturated) after *R23E10-GAL4*-driven FLP-mediated recombination. **i**, Maximum-intensity projections of fly brains expressing CsChrimson::tdTomato (magenta) or GCaMP (cyan) in a mutually exclusive fashion under the control of *R23E10-GAL4* after FLP-mediated recombination (left, whole midbrain; right, dFBN somata). Scale bars, 100 μm (**a**–**f**), 50 μm (inset in **d**, **g** (left), **h**, **i** (left)), 10 μm (**g** (right), **i** (right)).

Single-cell transcriptomic data^4^ and ultrastructural features^18^ identify glutamate as the fast-acting transmitter of dFBNs, in agreement with existing electrophysiological evidence that transmission from dFBNs (like glutamate release at many central synapses of *Drosophila*^19^) is inhibitory^20^. We confirmed this transmitter assignment anatomically and functionally. The subtraction of glutamatergic neurons from the *R23E10-GAL4* pattern (*R23E10-GAL4*∖*VGlut-GAL80*) retained two pairs of GABAergic cells in the suboesophageal zone and a handful of cholinergic neurons in the ventral nerve cord^21^ but excluded all dFBNs (Fig. 3a,b and Supplementary Video 3); conversely, the intersection of the *R23E10-GAL4* pattern with glutamatergic neurons (*R23E10 ∩ VGlut-GAL4*, created by reconstituting GAL4 from hemidrivers *R23E10-DBD* and *VGlut-p65AD*) highlighted dFBNs and two to four extraneous cells^22^ in the central brain but lacked cholinergic VNC sleep-promoting (VNC-SP) neurons^21^ of the nerve cord (Fig. 3c, Extended Data Fig. 4a–g and Supplementary Video 4). The extraneous cells, in all likelihood aMe24 neurons and one to three cells arborizing in the posterior lateral protocerebrum (Extended Data Fig. 3m), have no monosynaptic connections to dFBNs (Extended Data Fig. 3n) and no known roles in the regulation of sleep.

dFBNs coexpressing the optogenetic actuator CsChrimson and the glutamate sensor iGluSnFR responded to light pulses with stimulus-locked fluorescence transients in their axonal fields, verifying synaptic glutamate release (Fig. 3d,e). Like many glutamatergic neurons in the fly brain, dFBNs also transcribe genes encoding cholinergic machinery^4,22^ but repress the translation of these messages in a developmentally and perhaps functionally regulated manner^23^, as we confirmed for the vesicular acetylcholine transporter (Extended Data Fig. 4h). Underscoring the strict translational restriction on the glutamatergic identity of dFBNs in our experimental animals, the acetylcholine sensors iAChSnFR and GRAB_ACh_ detected no trace of cholinergic (co)transmission (Fig. 3d,e).

For a demonstration that dFBNs themselves are targets of dFBN-mediated inhibition, we divided the dFBN ensemble randomly, via stochastic recombination of GAL4-responsive FLP-out constructs^24^, into two disjoint groups containing either a green fluorophore (GCaMP for imaging; alternatively mCD8::GFP for targeted whole-cell electrophysiology) or CsChrimson::tdTomato (Extended Data Fig. 4i). Like focal applications of glutamate (Fig. 3f), optical stimulation of the CsChrimson-expressing set inhibited the complementary set of dFBNs, resulting in reductions of GCaMP fluorescence in imaging experiments (Fig. 3f) or in hyperpolarization and the suppression of spiking in current-clamp recordings from GFP-positive (and therefore CsChrimson-negative) neurons (Fig. 3g). The addition of tetrodotoxin (TTX) eliminated action potentials but not inhibitory postsynaptic potentials (Fig. 3g), which persist when transmission from directly connected terminals is triggered by light^24^. dFBN-to-dFBN communication is therefore monosynaptic.

### dFBNs promote sleep through SWA

**Fig. 4:**
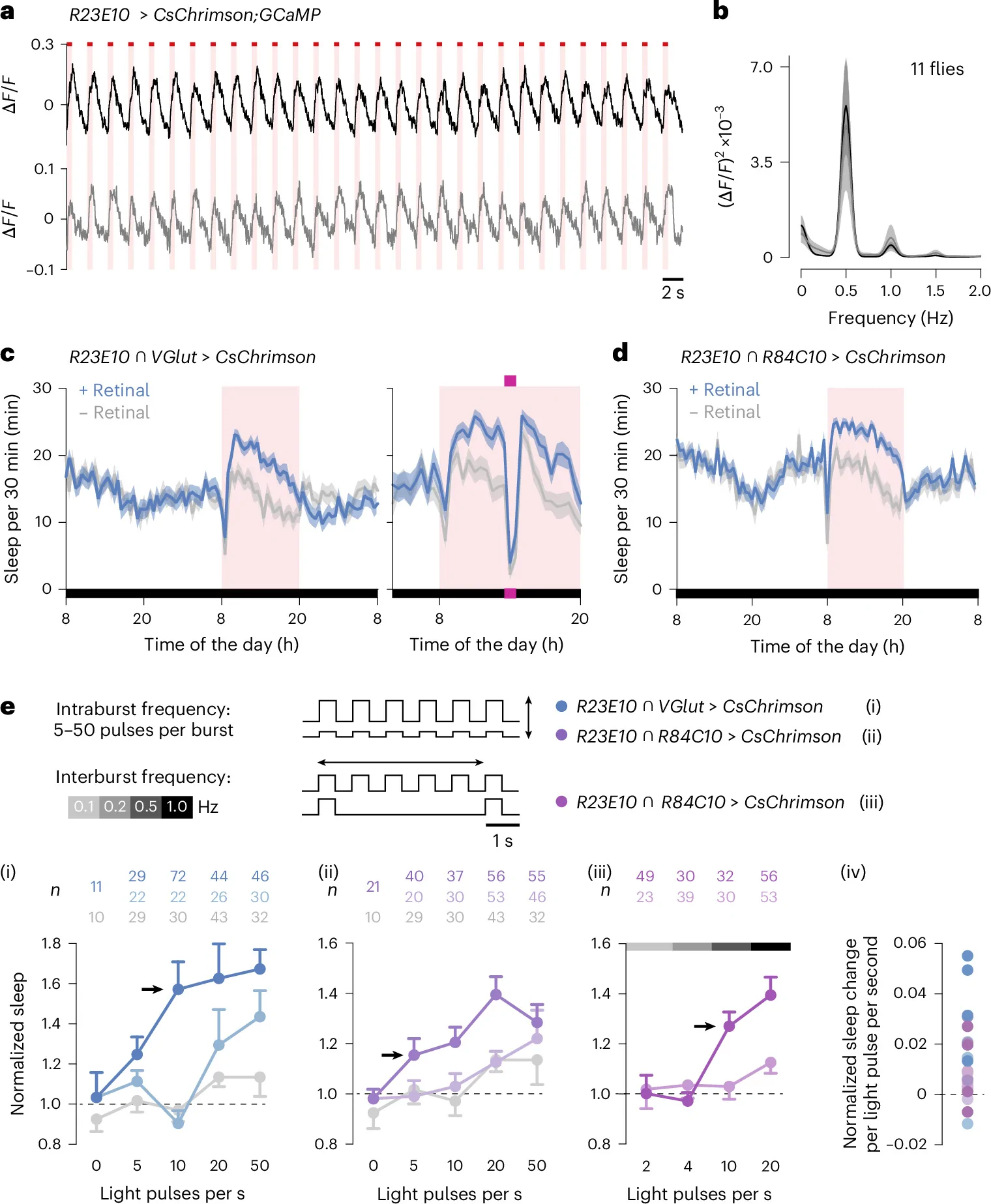
dFBN SWA promotes sleep. **a**, Simultaneous imaging of dFBN dendrites in both hemispheres during optogenetic replay of SWA (20 light pulses per 500-ms burst; 0.5 Hz interburst frequency). **b**, Power spectra of left (black) and right (gray) hemispheres. **c**, Sleep profiles of flies expressing CsChrimson under the control of *R23E10 ∩ VGlut-GAL4*, with or without retinal (left, *n* = 44 and 43, respectively), before, during and after optogenetic replay of SWA (20 light pulses per 500-ms burst; 1 Hz interburst frequency) (retinal effect: *P* = 0.0026; time × retinal interaction: *P* < 0.0001, two-way repeated-measures ANOVA). Near-ultraviolet illumination during optogenetic stimulation causes awakening (right, *n* = 19 and 24 flies with and without retinal, respectively). **d**, Sleep profiles of flies expressing CsChrimson under the control of *R23E10* ∩ *R84C10-GAL4*, with or without retinal (*n* = 56 and 43, respectively), before, during and after optogenetic replay of SWA (20 light pulses per 500-ms burst; 1 Hz interburst frequency) (retinal effect: *P* < 0.0001; time × retinal interaction: *P* < 0.0001, two-way repeated-measures ANOVA). **e**, Optogenetically induced sleep (normalized to non-retinal-fed controls) relative to carriers of an undriven *CsChrimson* transgene (same control group (gray) in i and ii; genotype effects: *P* < 0.0001 (i) and *P* = 0.0013 (ii), two-way ANOVA). In flies expressing CsChrimson under the control of *R23E10 ∩ VGlut-GAL4* (i) or *R23E10* ∩ *R84C10-GAL4* (ii and iii), optogenetic replay of SWA (dark colors) or tonic activity (pale colors) increases sleep as a function of the frequency and temporal structure of the optical pulse train (effects of the number of light pulses per second: *P* = 0.0223 (i), *P* = 0.0019 (ii), *P* < 0.0001 (iii); effects of temporal structure: *P* = 0.0149 (i), *P* = 0.0097 (ii), *P* = 0.0232 (iii); two-way ANOVA). Experiments in i and ii vary the number of light pulses per 500-ms burst at a constant interburst frequency of 1 Hz; iii varies the interburst frequency at a constant number of 20 light pulses per 500-ms burst. Sleep increases per light pulse are maximal during replay of naturalistic SWA with intraburst frequencies of 10–20 Hz (corresponding to 20–40 light pulses per 500-ms burst) and an interburst frequency of 0.5 Hz (iv and arrows in i–iii). Data are means ± s.e.m. (one-sided error bars in **e** for clarity); *n*, number of flies. For imaging details see Supplementary Table 1. For statistical details see Supplementary Table 2.

**Extended Data Fig. 5:**
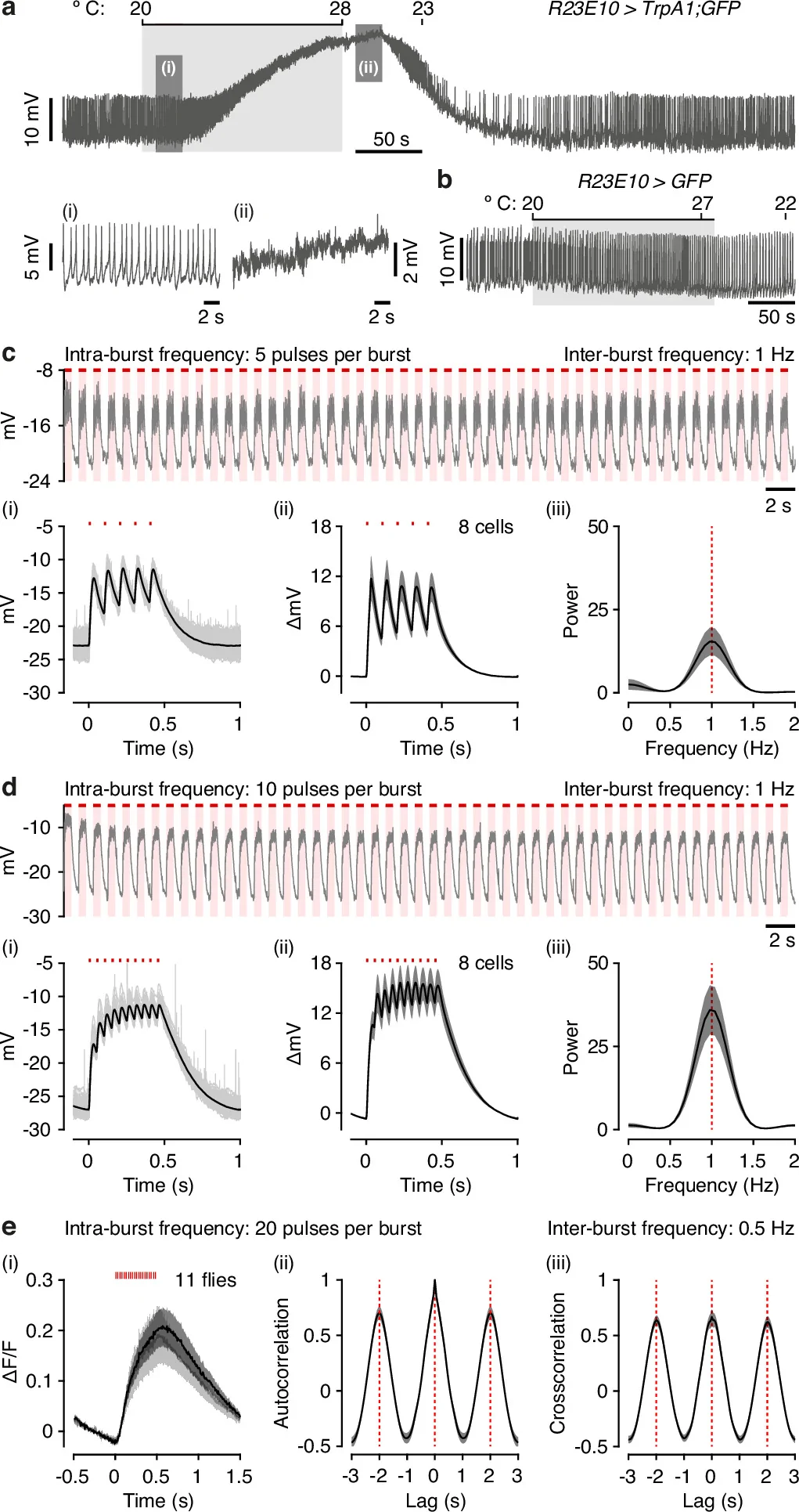
Pulsed optogenetic stimulation, but not thermogenetic depolarization, of dFBNs generates SWA. **a**, **b**, Voltage traces of dFBNs with (**a**) or without (**b**) *R23E10-GAL4*-driven expression of the thermosensitive cation channel TrpA1 during temperature ramps (light gray shading, heater on; top edge, 0 mV). Segments indicated by dark gray shading are shown at expanded *x* and *y* scales below. Regular spiking of the TrpA1-positive dFBN (**a**) at room temperature (i) ceases due to reversible depolarization block at ~28 ˚C (ii). The TrpA1-negative dFBN continues to spike at the elevated temperature (**b**). **c**, **d**, Voltage traces of dFBNs expressing CsChrimson under the control of *R23E10-GAL4* during optical stimulation with 5 (**c**) or 10 light pulses (**d**) per 500-ms burst and an inter-burst frequency of 1 Hz. Stimulus-locked averages of SWA cycles in the example traces (i; gray, individual bursts; black, average) or the voltage responses of 8 dFBNs (ii; gray, individual cells; black, average) are shown at expanded *x* and *y* scales below. Power spectra (iii; dark gray) were calculated after downsampling voltage traces to 500 Hz and correcting for slow linear drift. **e**, Simultaneous imaging of dFBN dendrites in both hemispheres during optogenetic stimulation of dFBNs coexpressing CsChrimson and GCaMP (500-ms bursts with an intra-burst frequency of 20 pulses and an inter-burst frequency of 0.5 Hz; see Fig. 4a for example GCaMP traces). Left, stimulus-locked voltage traces of 11 flies (black, left hemisphere; gray, right hemisphere). Center, autocorrelogram. Dashed red lines mark the autocorrelations at time lags of 0 and ±2 s. Right, crosscorrelogram of left and right hemispheres. Dashed red lines mark the crosscorrelations at time lags of 0 and ±2 s. Data are means ± s.e.m. For imaging details see Supplementary Table 1.

The dense interconnectivity of dFBNs (Extended Data Fig. 3) explains the prevalence of mutual inhibition in mosaics (Fig. 3f,g) but complicates attempts to control the activity of the population, with sometimes unforeseen consequences. Failures to observe increases in sleep after presumed (but unverified) activation of dFBNs led to a claim that the neurons are ineffective in promoting sleep^25^, when it was in reality the stimulation protocol—sustained depolarization with the thermosensitive cation channel TrpA1—that proved ineffective (Extended Data Fig. 5a,b). Pulsed illumination, which avoids depolarization block or a lasting cross-inhibitory stalemate (Fig. 4a,b and Extended Data Fig. 5c–e), readily induced sleep in flies expressing CsChrimson under the control of the intersectional drivers *R23E10 ∩ VGlut-GAL4* (Fig. 4c) or *R23E10* ∩ *R84C10-GAL4* (Fig. 4d), which lack expression in VNC-SP neurons^22^ (Extended Data Fig. 4c,f), even though dFBNs of both hemispheres were inevitably driven in synchrony rather than their natural antiphase (Fig. 4a and Extended Data Fig. 5e). Coincident exposures to 385-nm light canceled the sleep-promoting effect of optogenetic stimulation and led to awakening (Fig. 4c).

As in the regulation of sleep quality by clock neurons^26^ and the control of cortical SWA by the midline thalamus^27^, the temporal structure of the optical stimulus train mattered. Increases in sleep quotas followed the simulated convergence of alternating 500-ms action potential bursts from the left and right hemispheres in the axonal target layers of dFBNs, which we approximated by compressing 5, 10, 20 or 50 light pulses into one half of a 500:500 ms duty cycle (Fig. 4c–e), but were lacking or lessened when the same number of pulses were spread evenly across time, mimicking tonic activity (Fig. 4e). The higher the intraburst stimulation frequency was (a simulated increase in SWA power; Extended Data Fig. 5c,d), the stronger was the sleep-promoting effect up to a saturation frequency of ~40 Hz (Fig. 4e), approximating the peak intraburst firing rate in our recordings of spontaneous dFBN activity (Fig. 1d). At the saturation frequency, 500-ms bursts needed to recur every 1 or 2 s to induce sleep; interburst intervals exceeding 5 s proved as ineffective as did tonic stimulation at rates below 10 Hz (Fig. 4e). This systematic exploration of stimulation parameters along three axes (tonic versus burst stimulation and variable intra- and interburst frequencies) showed that sleep increases per pulse were maximal during the optogenetic replay^28^ of activity patterns that closely resembled endogenously generated SWA (Fig. 4e). SWA thus has a privileged role in the promotion of sleep by dFBNs.

**Fig. 5:**
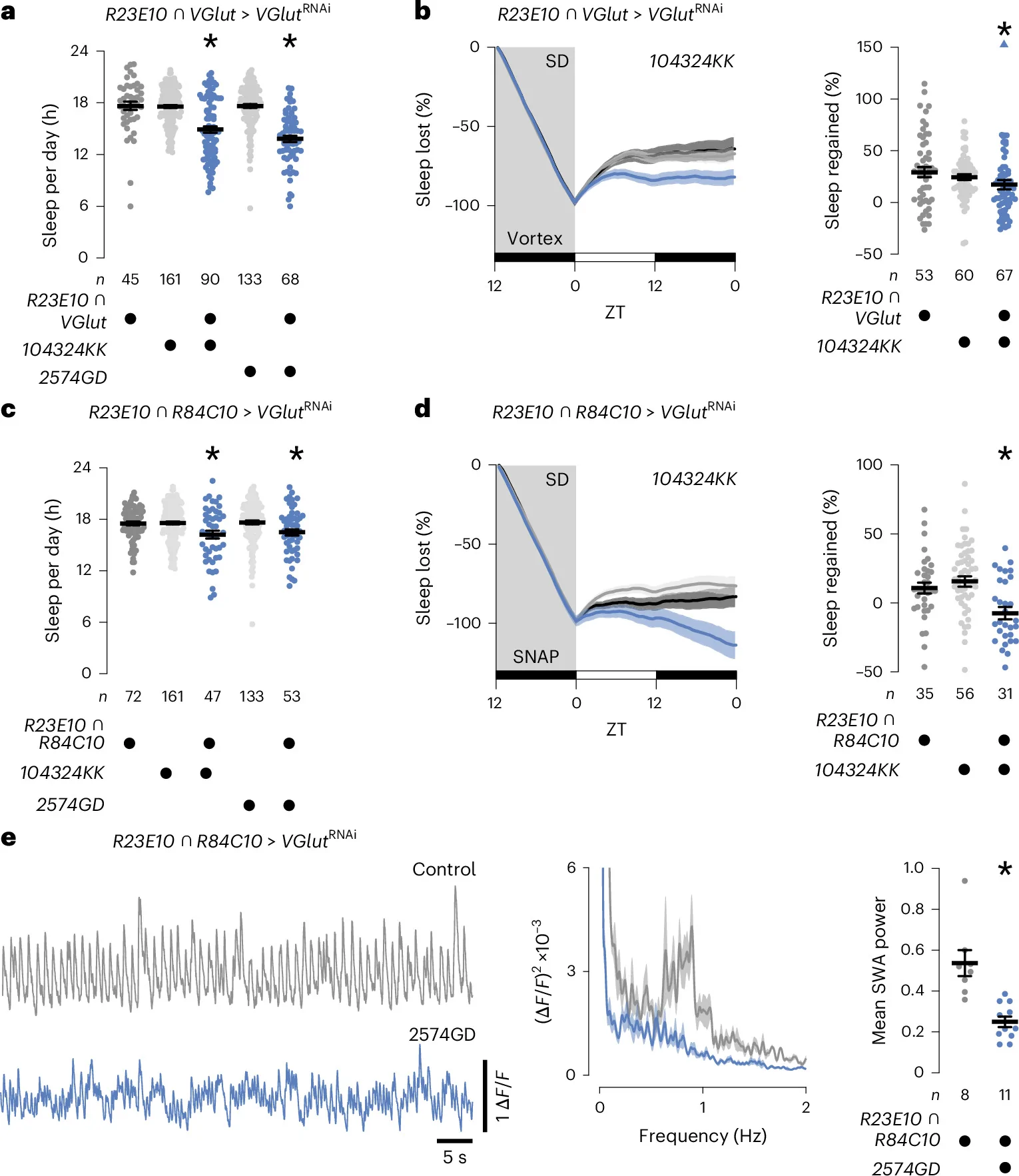
Impaired glutamatergic transmission from dFBNs disrupts sleep and SWA. **a**,**b**, *R23E10 ∩ VGlut-GAL4*-driven expression of *VGlut*^RNAi^ reduces daily sleep (**a**, *P* < 0.0001, Dunn’s test after Kruskal–Wallis ANOVA) and the time course and percentage of sleep regained after deprivation (SD) (**b**, left, genotype effect: *P* = 0.0268; time x genotype interaction: *P* = 0.0171, two-way repeated-measures ANOVA; right, *P* ≤ 0.0246, Dunn’s test after Kruskal–Wallis ANOVA). One data point exceeding the *y*-axis limit is plotted as a triangle at the top of the right graph; mean and s.e.m. are based on the actual values. **c**,**d**, *R23E10* ∩ *R84C10*-driven expression of *VGlut*^RNAi^ reduces daily sleep (**c**, left, *P* ≤ 0.0203, Holm-Šídák test after ANOVA) and the time course and percentage of sleep regained after deprivation (SD) (**d**, left, genotype effect: *P* = 0.0039; time × genotype interaction: *P* < 0.0001, two-way repeated-measures ANOVA; right, *P* ≤ 0.0251, Dunn’s test after Kruskal–Wallis ANOVA) without altering waking locomotor activity (Extended Data Fig. 6). Some parental controls in **a**–**d** are common to both experiments, which were conducted in parallel. **e**, Example dendritic GCaMP traces (left) and power spectra (center) of dFBNs expressing GCaMP without or with *VGlut*^RNAi^ under the control of *R23E10* ∩ *R84C10*-*GAL4*. *VGlut*^RNAi^ reduces the average SWA power (right, *P* < 0.0001, two-sided Mann–Whitney test). Data are means ± s.e.m.; *n*, number of flies; asterisks indicate significant differences (*P* < 0.05) in planned pairwise comparisons. For imaging details see Supplementary Table 1. For statistical details see Supplementary Table 2.

**Extended Data Fig. 6:**
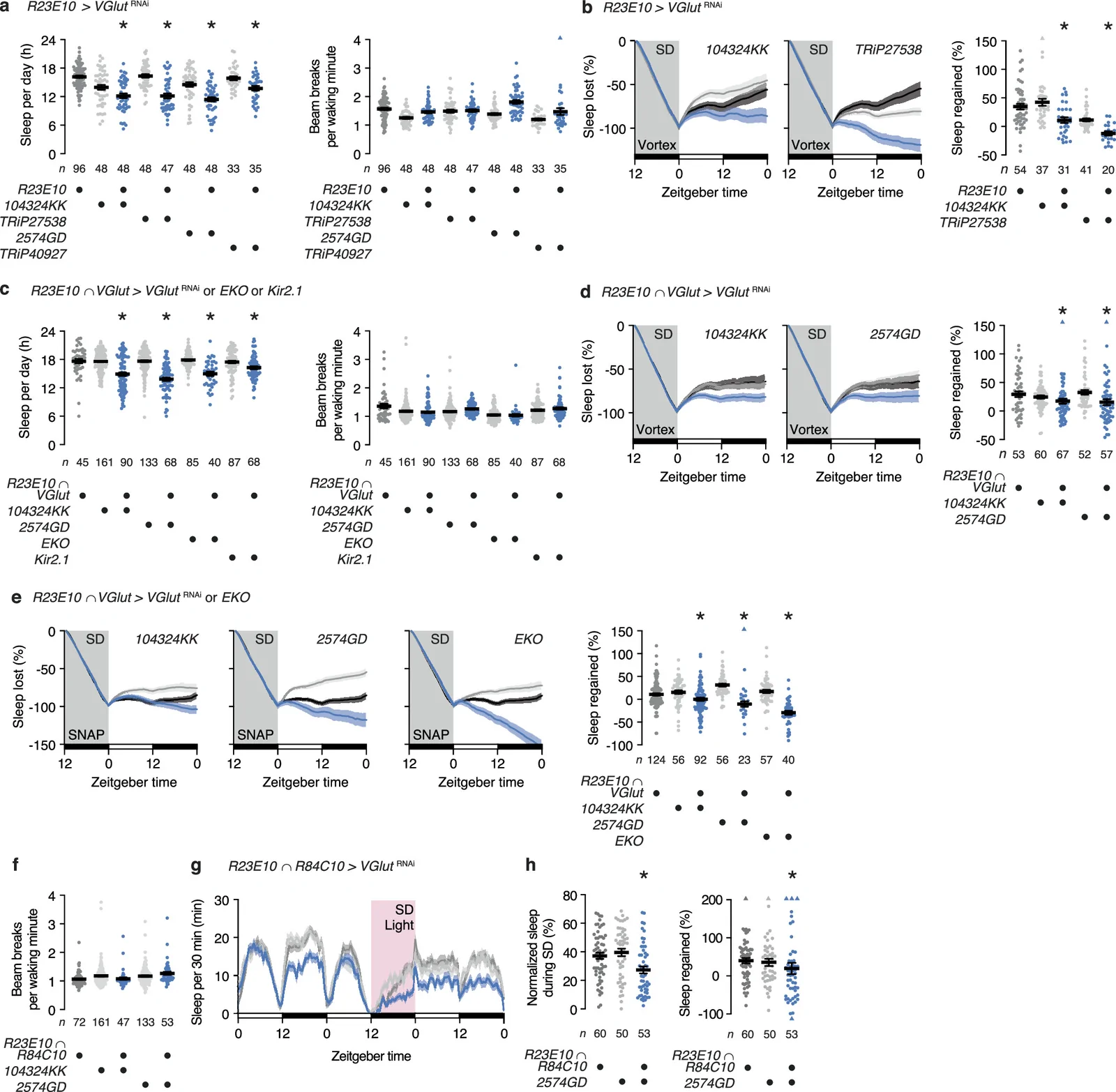
Different strategies for interfering with the function of dFBNs have concordant effects on sleep. **a**, **b**, *R23E10-GAL4*-driven expression of *VGlut*^RNAi^ reduces daily sleep (**a**, left, *P* ≤ 0.0077, Holm-Šídák test after ANOVA) and the time courses and percentages of sleep regained after deprivation by vortex (SD) (**b**, left panels, genotype effects: *P* ≤ 0.0176, time × genotype interactions: *P* < 0.0001, two-way repeated-measures ANOVA; right panel, *P* ≤ 0.0109, Dunn’s test after Kruskal-Wallis ANOVA) without altering waking locomotor activity (**a**, right, *P* ≥ 0.1601 relative to ≥1 parental control, Dunn’s test after Kruskal-Wallis ANOVA). **c**, *R23E10* ∩ *VGlut-GAL4*-driven expression of *VGlut*^RNAi^ or EKO or Kir2.1 reduces daily sleep (left, *P* ≤ 0.0135, Dunn’s test after Kruskal-Wallis ANOVA) without altering waking locomotor activity (right, *P* > 0.9999 relative to ≥1 parental control, Dunn’s test after Kruskal-Wallis ANOVA). The left panel is partially reproduced from Fig. 5a. **d**, *R23E10* ∩ *VGlut-GAL4*-driven expression of *VGlut*^RNAi^ reduces the time courses and percentages of sleep regained after deprivation by vortex (SD) (left panels, genotype effects: *P* ≤ 0.0314, time × genotype interactions: *P* ≤ 0.0171, two-way repeated-measures ANOVA; right panel, *P* ≤ 0.0429, Dunn’s test after Kruskal-Wallis ANOVA). The panels are partially reproduced from Fig. 5b. **e**, *R23E10 ∩ VGlut-GAL4*-driven expression of *VGlut*^RNAi^ or EKO reduces the time courses and percentages of sleep regained after deprivation (SD) by SNAP (left panels, genotype effects: *P* ≤ 0.0087, time × genotype interactions: *P* < 0.0001, two-way repeated-measures ANOVA; right panel, *P* ≤ 0.0278, Dunn’s test after Kruskal-Wallis ANOVA). **f**, *R23E10* ∩ *R84C10*-driven expression of *VGlut*^RNAi^ does not alter waking locomotor activity (*P* ≥ 0.1481 relative to ≥1 parental control, Dunn’s test after Kruskal-Wallis ANOVA). Some parental controls in **c** and **f** are common to both experiments, which were conducted in parallel. **g**, Sleep profiles of flies expressing *VGlut*^RNAi^ under the control of *R23E10* ∩ *R84C10* before, during and after sleep deprivation by flickering light stimulation (genotype effect: *P* < 0.0001, time × genotype interaction: *P* < 0.0001, two-way repeated-measures ANOVA). **h**, *R23E10* ∩ *R84C10*-driven expression of *VGlut*^RNAi^ increases sleep loss during flickering light stimulation (left, *P* ≤ 0.0069, Dunn’s test after Kruskal-Wallis ANOVA) and reduces the percentage of sleep regained after visual deprivation (right, *P* ≤ 0.0426, Dunn’s test after Kruskal-Wallis ANOVA). Data are means ± s.e.m.; *n*, number of flies; asterisks indicate significant differences (*P* < 0.05) in planned pairwise comparisons. Data points exceeding the *y*-axis limits are plotted as triangles at the extremes of graphs; mean and s.e.m. are based on the actual values. For statistical details see Supplementary Table 3.

**Extended Data Fig. 7:**
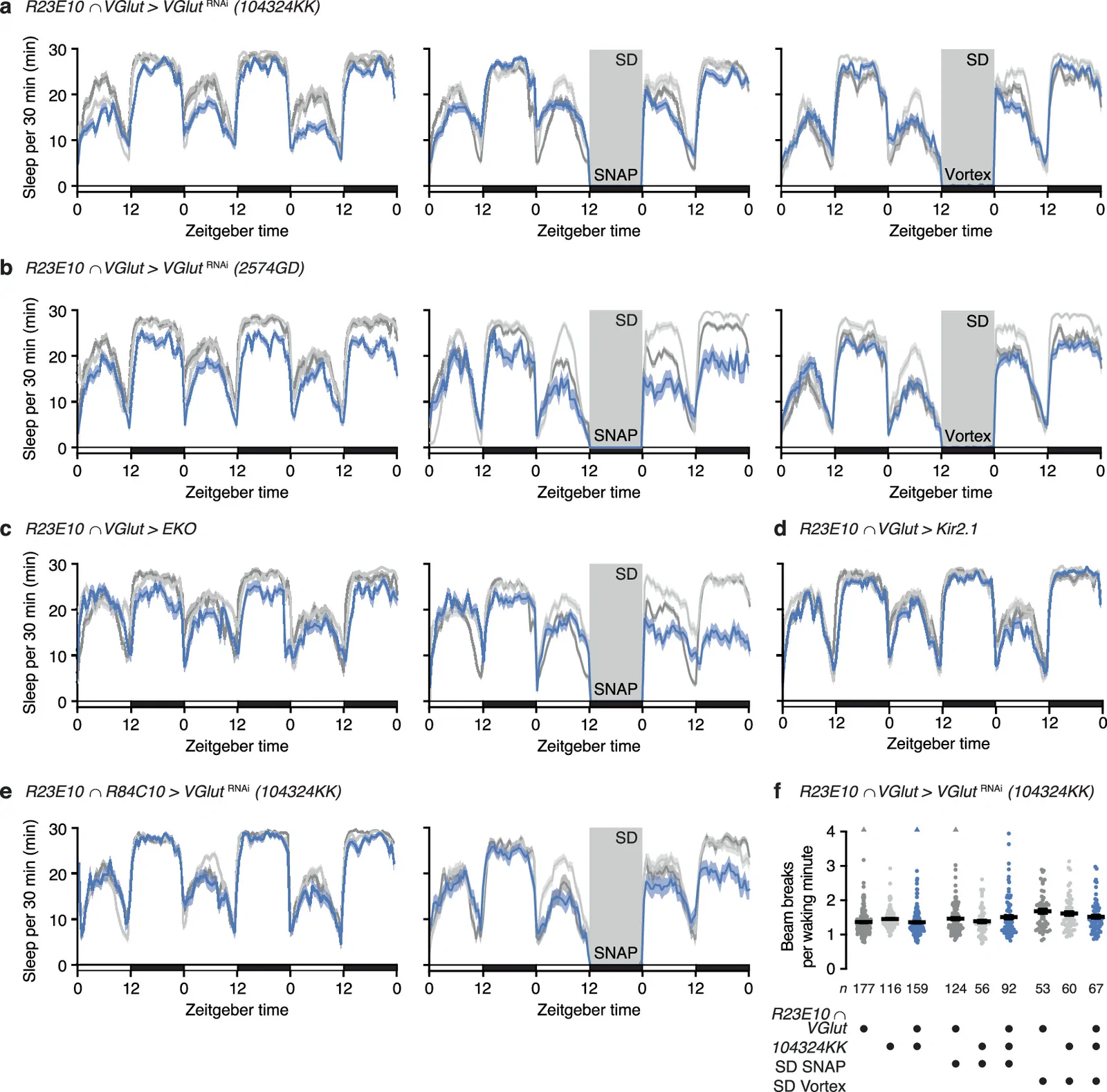
Sleep profiles during unperturbed sleep, two forms of sleep deprivation and recovery. **a**–**e**, Sleep profiles of experimental flies (blue) expressing *VGlut*^RNAi^ (**a**, **b**, **e**), EKO (**c**) or Kir2.1 (**d**) under the control of *R23E10* ∩ *VGlut-GAL4* (**a**–**d**) or *R23E10* ∩ *R84C10-GAL4* (**e**) and parental controls (gray). Sample sizes and symbol colors as in Fig. 5 and Extended Data Fig. 6, which report data from the same experiments. Left column (**a**–**c**) and **d**, unperturbed sleep; center column (**a**–**c**, **e**), 12-h sleep deprivation (SD) on day 2 via the sleep-nullifying apparatus (SNAP); right column (**a**–**b**), 12-h sleep deprivation (SD) on day 2 by vortex. Two-way repeated-measures ANOVA detects significant genotype effects on sleep (*P* ≤ 0.0091) and significant time × genotype interactions (*P* < 0.0001) during all quantification periods (unperturbed sleep recordings, days 2 and 3; sleep deprivation experiments, day 3). **f**, Waking locomotor activity in flies expressing *VGlut*^RNAi^ under the control of *R23E10* ∩ *VGlut-GAL4* and parental controls, before and after sleep deprivation (SD) by SNAP or vortex. Three data points exceeding the *y*-axis limit are plotted as triangles at the top of the graph; mean and s.e.m. are based on the actual values. Mildly elevated waking activity after sleep deprivation rules out injury (effect of sleep history: *P* < 0.0001, effect of SD method: *P* = 0.2287, genotype effect: *P* = 0.5877, three-way ANOVA). Data are means ± s.e.m.; *n*, number of flies. For statistical details see Supplementary Table 3.

**Extended Data Fig. 8:**
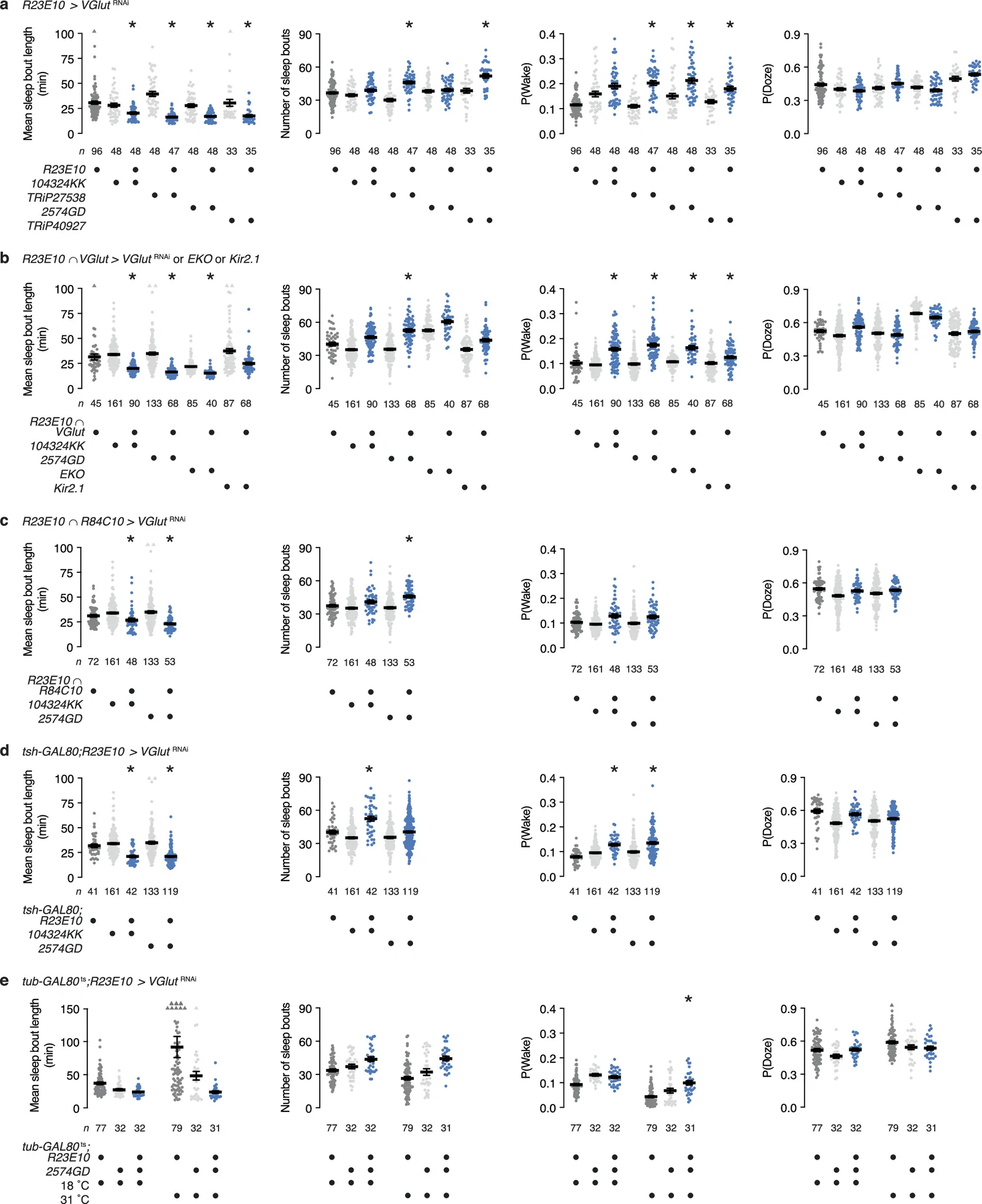
Interference with the function of dFBNs decreases sleep bout duration and increases the sleep-to-wake transition probability. **a**–**e**, Sleep architecture and sleep–wake transition probabilities of experimental flies (blue) expressing *VGlut*^RNAi^ (**a**–**e**), EKO (**b**) or Kir2.1 (**b**) under the control of *R23E10-GAL4* (**a**), *R23E10* ∩ *VGlut-GAL4* (**b**), *R23E10* ∩ *R84C10-GAL4* (**c**) or *R23E10-GAL4* in the presence of *teashirt-GAL80* (**d**) or the temperature-sensitive repressor *tub-GAL80*^*ts*^ (**e**) and parental controls (gray). Sample sizes and symbol colors as in Fig. 5 and Extended Data Fig. 6 and 9, which report data from the same experiments. Sleep bout length decreases and P(Wake) increases after chronic (**a**–**d**, *P* ≤ 0.0478 for bout length and *P* ≤ 0.0478 for P(Wake), Dunn’s test after Kruskal-Wallis ANOVA) or acute interference with the function of dFBNs (**e**, temperature × genotype interaction: *P* = 0.0411 for bout length and *P* = 0.0149 for P(Wake), two-way ANOVA). There is a trend towards increased sleep bout numbers across several driver lines and modes of interference (**a**–**d**); P(Doze) is unaltered (**a**–**d**, *P* ≥ 0.1639, Dunn’s test after Kruskal-Wallis ANOVA; **e**, temperature × genotype interaction: *P* = 0.1537, two-way ANOVA). Data are means ± s.e.m.; n, number of flies; asterisks indicate significant differences (*P* < 0.05) in planned pairwise comparisons. Data points exceeding the *y*-axis limits are plotted as triangles at the top of graphs; mean and s.e.m. are based on the actual values. For statistical details see Supplementary Table 3.

**Extended Data Fig. 9:**
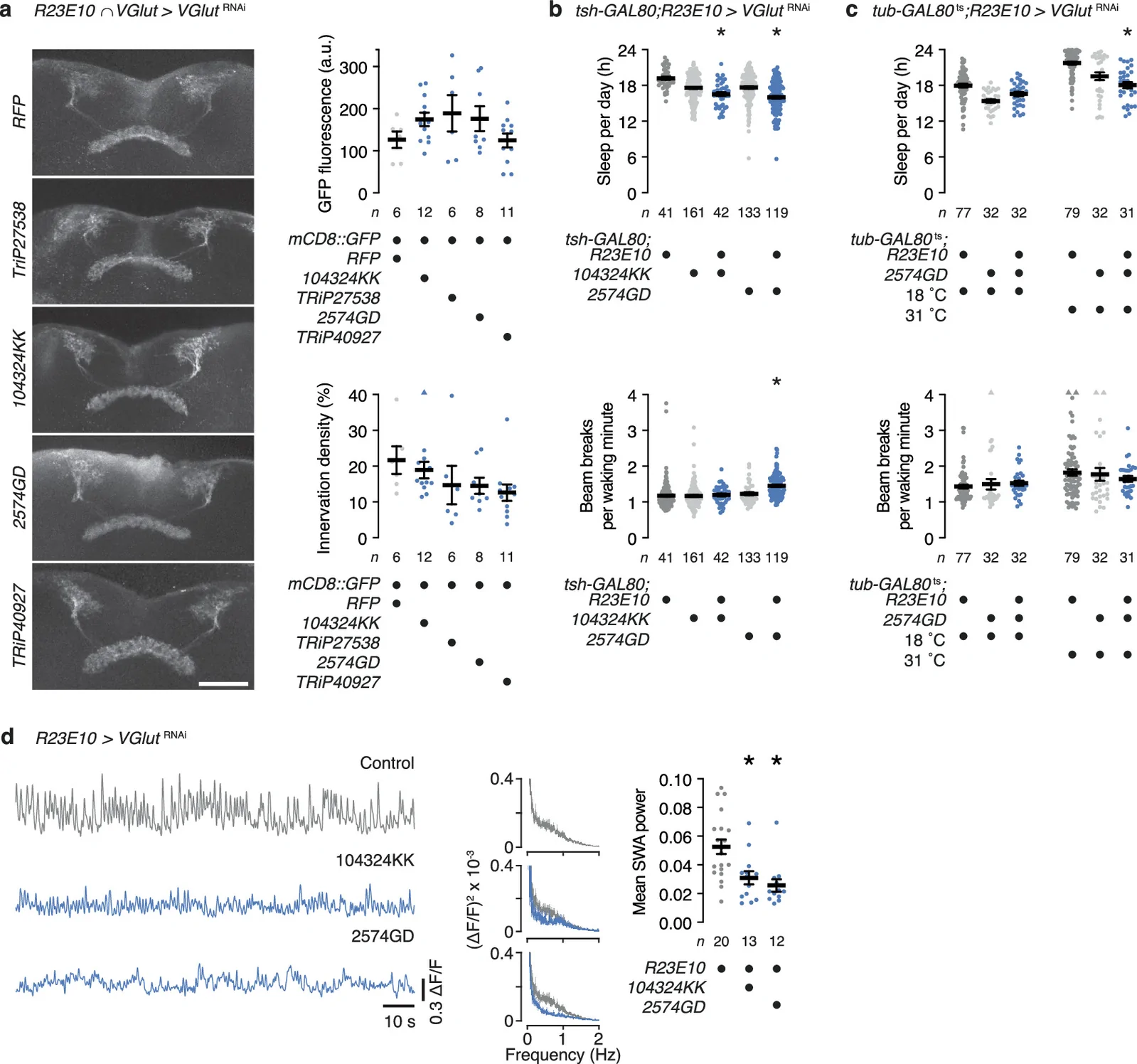
Chronic and acute interference with glutamatergic transmission from dFBNs reduces sleep and SWA without altering neuronal morphology. **a**, Maximum-intensity projections of dFBNs in flies expressing RFP or *VGlut*^RNAi^ under the control of *R23E10* ∩ *VGlut-GAL4* (left). GFP fluorescence (top right) and innervation density (bottom right) of dFBN projections is unaffected by the expression of *VGlut*^RNAi^ (*P* ≥ 0.0608, Dunn’s test after Kruskal-Wallis ANOVA). **b**, *R23E10*-driven expression of *VGlut*^RNAi^ reduces daily sleep in the presence of *teashirt-GAL80* (top, *P* ≤ 0.0174, Dunn’s test after Kruskal-Wallis ANOVA). Expression of *VGlut*^RNAi 2574GD^ (*P* ≤ 0.0001), but not of *VGlut*^RNAi 104324KK^, increases waking locomotor activity (bottom, *P* ≥ 0.5732, Dunn’s test after Kruskal-Wallis ANOVA). **c**, Temperature-inducible, adult-specific expression of *VGlut*^RNAi^ under the control of *R23E10-GAL4* reduces daily sleep (top, temperature × genotype interaction: *P* = 0.0035, two-way ANOVA) without altering waking locomotor activity (bottom, temperature × genotype interaction: *P* = 0.3797, two-way ANOVA). **d**, Example dendritic GCaMP traces (left) and power spectra (center) of dFBNs expressing GCaMP without or with *VGlut*^RNAi^ under the control of *R23E10-GAL4*. *VGlut*^RNAi^ reduces the average SWA power (right, *P* = 0.0005, Kruskal-Wallis ANOVA). Data are means ± s.e.m.; *n*, number of flies; asterisks indicate significant differences (*P* < 0.05) in planned pairwise comparisons. Data points exceeding the *y*-axis limits are plotted as triangles at the top of graphs; mean and s.e.m. are based on the actual values. Scale bar, 50 μm. For statistical details see Supplementary Table 3.

To add evidence of necessity to this demonstration of sufficiency, we incapacitated dFBNs by RNA-mediated interference (RNAi) with the expression of VGlut in the entire *R23E10-GAL4* territory, of which dFBNs are the main glutamatergic members (Extended Data Fig. 4a–c), or by antagonizing glutamate release or membrane depolarization with the help of the intersectional drivers *R23E10 ∩ VGlut-GAL4* or *R23E10* ∩ *R84C10-GAL4* (Extended Data Fig. 4c,f), with concordant results (Fig. 5a–d and Extended Data Figs. 6 and 7). The depletion of VGlut from dFBNs or the introduction of the low-voltage-activated or inwardly rectifying potassium channels EKO or Kir2.1 reduced baseline and rebound sleep after sleep deprivation, using any of three—mechanical and nonmechanical—methods (Fig. 5a–d and Extended Data Figs. 6 and 7). Sleep losses were accompanied by sleep fragmentation (Extended Data Fig. 8) and occurred without potentially confounding waking hyperactivity (Extended Data Fig. 6a,c,f) or innervation defects that might have followed chronically impaired glutamatergic transmission (Extended Data Fig. 9a). The sleep deficits persisted when *teashirt-GAL80* eliminated *R23E10-GAL4*-driven *VGlut*^RNAi^ transgene expression in the ventral nerve cord (Extended Data Figs. 4e and 9b) or a *tubulin-GAL80*^ts^-mediated block was relieved only after eclosion (Extended Data Fig. 9c). Consistent with a critical role of reciprocal inhibition in antiphase oscillations^10–13^, interference with glutamatergic transmission also disrupted SWA (Fig. 5e and Extended Data Fig. 9d).

### SWA encodes sleep pressure

**Fig. 6:**
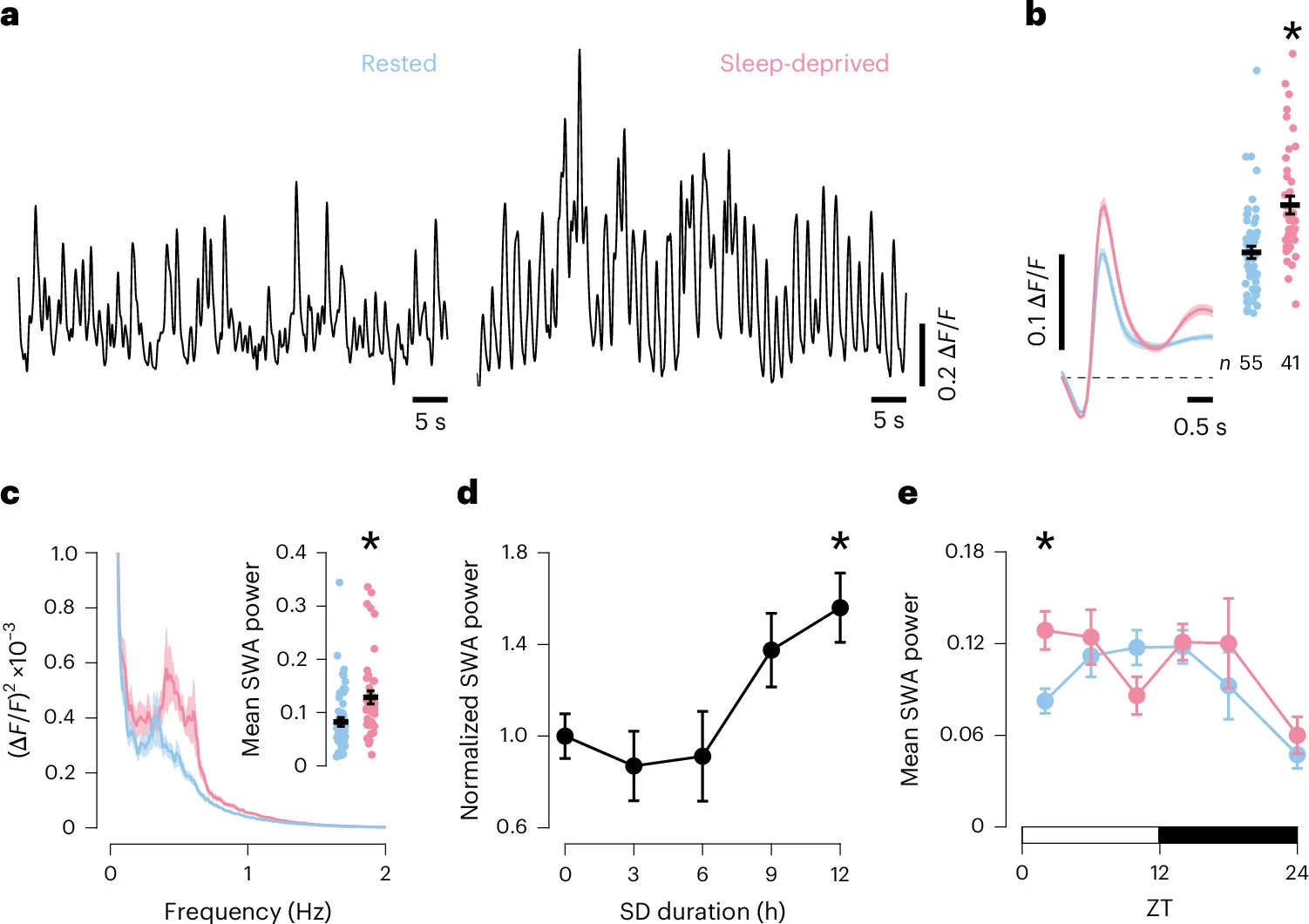
dFBN SWA encodes sleep pressure. **a**, Example GCaMP traces of dFBN dendrites at ZT 0–4, after unperturbed rest (blue) or sleep deprivation between ZT 12 and 24 (red). **b**,**c**, Sleep deprivation increases the average GCaMP transient (**b**, left), its peak amplitude (**b**, right, *P* < 0.0001, two-sided Mann–Whitney test) and SWA power (**c**, *P* = 0.0005, two-sided Mann–Whitney test). **d**, SWA power (normalized to unperturbed controls) at ZT 0–4 as a function of the duration of prior sleep deprivation (SD) beginning at ZT 12, 15, 18 and 21 and ending at ZT 24 (*P* = 0.0056, Kruskal–Wallis ANOVA). *n* at SD durations of 0, 3, 6, 9 and 12 h: 55, 14, 12, 47, 41 (same data at 0- and 12-h time points as in **b** and **c**). **e**, Mean SWA power during the course of a day in rested (blue) and sleep-deprived (red) flies (effect of sleep history: *P* = 0.0001, Kruskal–Wallis ANOVA; effect of ZT in rested flies: *P* = 0.0010, Kruskal–Wallis ANOVA). *n* of rested and sleep-deprived flies, respectively, at the given ZT were 0–4: 55, 41; 4–8: 32, 14; 8–12: 28, 14; 12–16: 34, 15; 16–23: 13, 9; 23–4: 9, 11. Data are means ± s.e.m.; *n*, number of flies; asterisks indicate significant differences (*P* < 0.05) in planned pairwise comparisons. Same dataset as in Figs. 1a and 2 and Extended Data Figs. 1h–j and 2. For imaging details see Supplementary Table 1. For statistical details see Supplementary Table 2.

The sleep losses following the dissolution of SWA hint, and the sleep gains due to SWA replay over other forms of stimulation strongly suggest, that slow waves have an instructive role in the translation of sleep pressure into sleep (Figs. 4 and 5 and Extended Data Figs. 6–9). The electroencephalogram (EEG) during mammalian non-rapid eye movement (NREM) sleep^8,29^ is characterized by SWA that reflects synchronous transitions of cortical neurons between depolarized UP and hyperpolarized DOWN states^30^, not unlike those of rhythmic dFBNs (Fig. 1d). Cortical SWA is a classical EEG marker of sleep pressure: SWA levels at sleep onset increase after prolonged wakefulness and decrease after sleep^8,31^. To search for analogous modulations of dFBN activity, we compared SWA in flies that had been mechanically sleep-deprived for different spans of time with rested controls. Overnight sleep deprivation for 12 h (again using two different methods to similar effect) increased the average peak amplitude of GCaMP transients and the mean SWA power (Fig. 6a–c and Extended Data Fig. 2d,e) but left other frequency bands unchanged (Fig. 6c). SWA grew roughly linearly with the duration of sleep deprivation during the first half of the night and showed little or no further increases during the second half (Fig. 6d and Extended Data Fig. 2f). As flies recovered, Ca^2+^ transients and SWA power decayed back to baseline over the course of 12–24 h (Fig. 6e and Extended Data Fig. 2g). Following an unperturbed night of rest, in contrast, Ca^2+^ transients and SWA were at their minimum in the morning (zeitgeber time (ZT) 0–4), rose naturally during the day to an evening peak (ZT 12) and declined again during the night (Fig. 6e and Extended Data Fig. 2g).

**Fig. 7:**
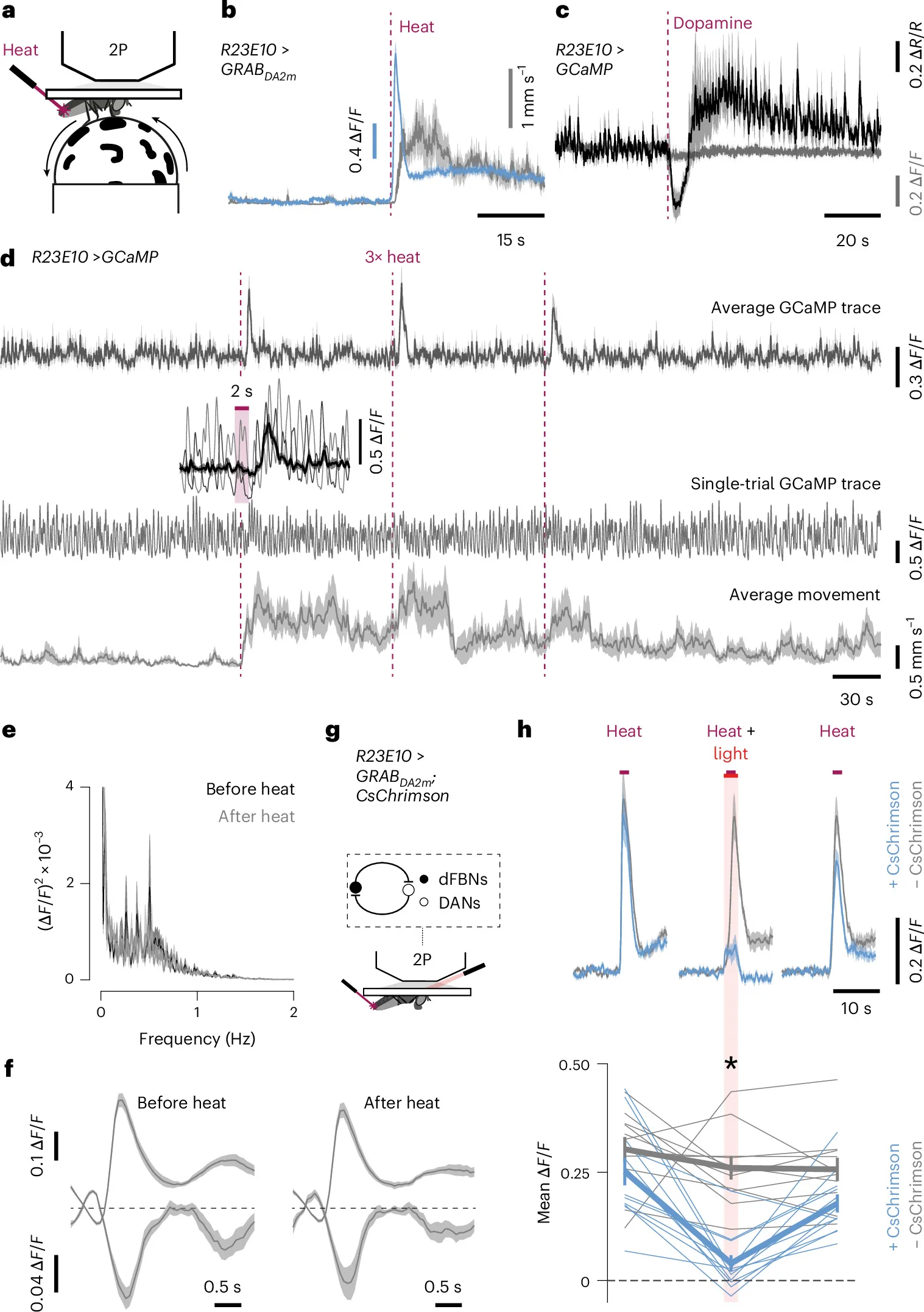
SWA persists during arousal. **a**, Movement tracking and imaging during the application of arousing heat. **b**, Heat (dashed red line) elicits dopamine release onto dFBN dendrites expressing GRAB_DA2m_ (blue) and stimulates locomotion (gray) (*n* = 9 flies). **c**, Imaging of dFBN dendrites during pressure ejection of dopamine (*n* = 4 flies, 8 ROIs). Average GCaMP trace (black) normalized to mCD4::tdTomato fluorescence (Δ*R*/*R*) and aligned to a 640-ms dopamine pulse. Average Δ*F*/*F* of mCD4::tdTomato is shown for comparison (gray). **d**, Dendritic GCaMP and locomotor traces before, during and after three heat applications (dashed red lines). Top, cancellation of SWA in the average GCaMP trace (*n* = 11 flies) reveals rebound responses aligned to the offset of heat. Lower end of scale bar, Δ*F*/*F* = 0. Center, GCaMP trace of a single trial. Lower end of scale bar, Δ*F*/*F* = –0.2. The inset shows segments of GCaMP traces in both hemispheres during one heat application and the corresponding segment of the average trace (black) at expanded *x* and *y* scales. Bottom: average locomotor speed. Lower end of scale bar, 0 mm per second. **e**,**f**, Power spectra (**e**) and transient-aligned average ipsi- (top) and contralateral (bottom) GCaMP traces (**f**) in eight flies where both hemispheres were imaged, during 140-s windows before and after the application of heat. **g**, Optogenetic stimulation of dFBNs during the application of heat probes for an inhibitory flip-flop arrangement of dFBNs and arousing dopaminergic neurons (DANs). **h**, Light during heat application inhibits dopamine release onto CsChrimson-positive (blue, *n* = 12 flies) but not CsChrimson-negative dFBNs (gray, *n* = 11 flies). Top: example traces of three applications of heat (spaced 30 min apart) to the same flies. Traces were smoothed with a five-element (~300 ms) moving-average filter for display. Bottom: dFBN stimulation reduces mean GRAB_DA2m_ fluorescence during heat application (heat × CsChrimson interaction: *P* = 0.0017, two-way repeated-measures ANOVA). Thin lines, individual flies; thick lines, population averages. Data are means ± s.e.m.; the asterisk indicates a significant difference (*P* < 0.05) in a planned pairwise comparison. For imaging details see Supplementary Table 1. For statistical details see Supplementary Table 2.

Previous reports of sleep-related rhythms^32,33^, and in particular observations of SWA in parts of the central complex^34,35^ (R5 neurons and dFBNs), noted but did not explore possible parallels with NREM sleep because SWA was characterized without a simultaneous behavioral readout of vigilance state. It is thus unclear if the occurrence of dFBN oscillations defines a slow-wave sleep stage as in vertebrates or if SWA tracks variations in sleep pressure throughout the sleep–wake cycle. To resolve this point, we examined whether dFBN oscillations persisted during a verified state of arousal. Head-fixed flies expressing either the dopamine sensor GRAB_DA2m_ or GCaMP in dFBNs were placed on a spherical treadmill and roused with a pulse of infrared laser light focused on their abdomina (Fig. 7a). In line with the notion that dopaminergic neurons projecting to the fan-shaped body mediate arousal by inhibiting dFBNs^6,36,37^, activating the laser for 2 s elicited a movement bout along with dopamine release onto the neurons’ dendrites (but not axons) (Fig. 7b). Like spontaneous spiking during dendritic applications of exogenous dopamine or optogenetic stimulation of dopaminergic neurons^6^, Ca^2+^ oscillations collapsed briefly during a short heat stimulus but resumed thereafter, following a powerful postinhibitory rebound (Fig. 7c–f), while pharmacological doses of dopamine produced pauses lasting many seconds^6^ (Fig. 7c).

Coincident optogenetic activation of dFBNs reduced the amplitude of heat-evoked dopamine transients (Fig. 7g,h), indicating that dFBNs and dopaminergic neurons antagonize each other in an arrangement reminiscent of the inhibitory flip-flop switch between sleep- and wake-promoting neurons in the mammalian hypothalamus^38^.

### Glutamate release adjusts to sleep pressure

**Fig. 8:**
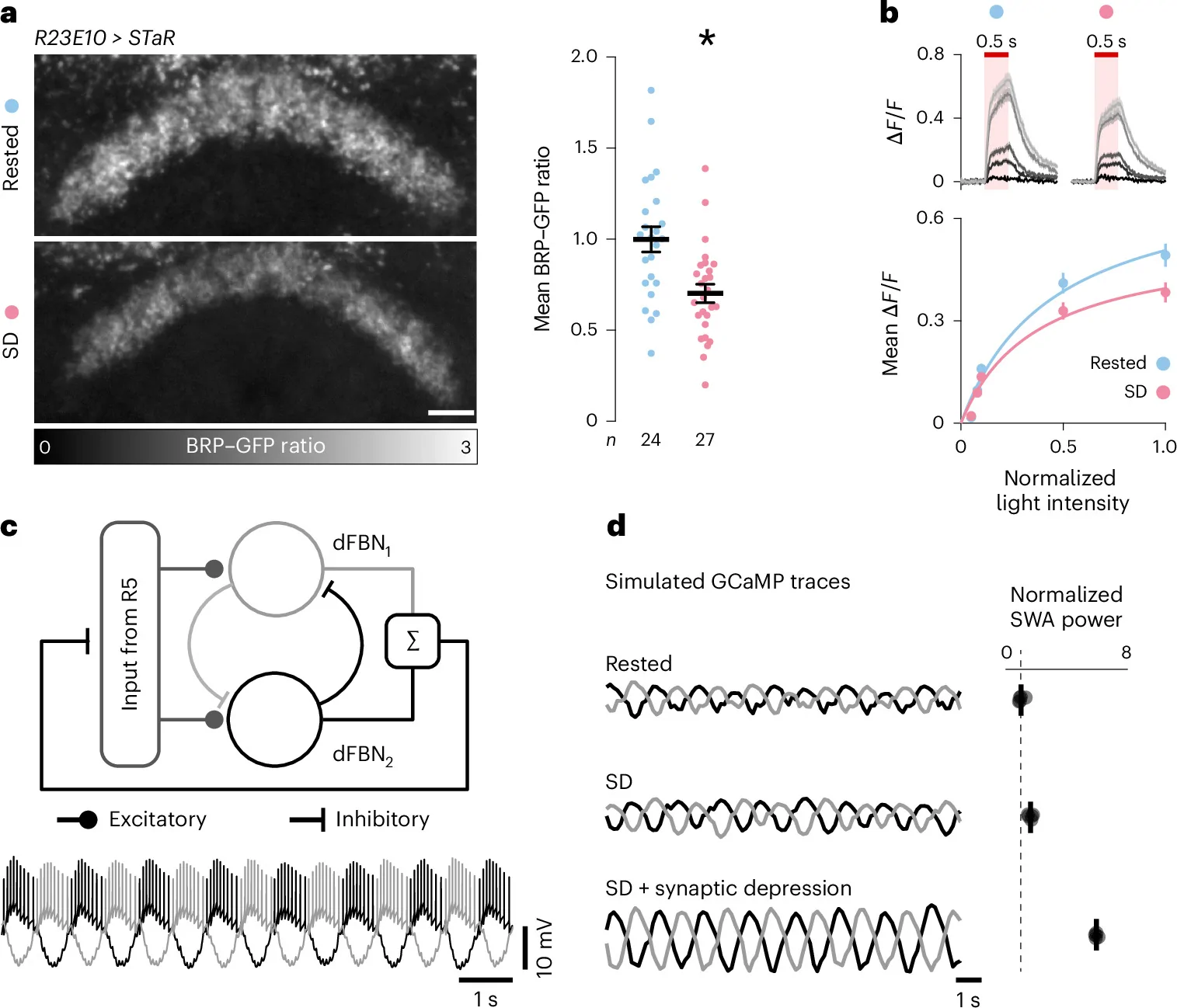
Efferent dFBN synapses depress after sleep deprivation. **a**, Summed-intensity projections of V5-tagged endogenous BRP in dFBN axons coexpressing mCD8::GFP. Emission ratios are intensity coded according to the key at the bottom and decrease after sleep deprivation (SD) (*P* = 0.0009, two-sided *t*-test). **b**, Sleep deprivation reduces glutamate release elicited by optogenetic stimulation of dFBNs (light intensity × sleep history interaction: *P* = 0.0002, two-way repeated-measures ANOVA). Top: iGluSnFR traces (*n* = 11 rested and 12 SD flies). Shades of gray from dark to light show increasing light intensities normalized to peak (25 mW cm^−2^). Bottom: mean iGluSnFR Δ*F*/*F* during illumination in rested (blue) and SD flies (red) as functions of light intensity. Data are fitted by saturation hyperbolas. **c**, Minimal model of two dFBNs with reciprocal inhibitory connections in a simplified feedback circuit with a pool of excitatory Poisson units. Top: model schematic. Bottom: simulated voltage traces of dFBNs color coded as on top. **d**, Simulated GCaMP traces (left) and mean SWA power (right) at baseline and after sleep deprivation, in the absence and presence of plastic feedback connections with Poisson units (*n* = 5 simulations). Data are means ± s.e.m.; the asterisk indicates a significant difference (*P* < 0.05) in a planned pairwise comparison. Scale bar, 10 μm. For imaging details see Supplementary Table 1. For statistical details see Supplementary Table 2.

**Extended Data Fig. 10:**
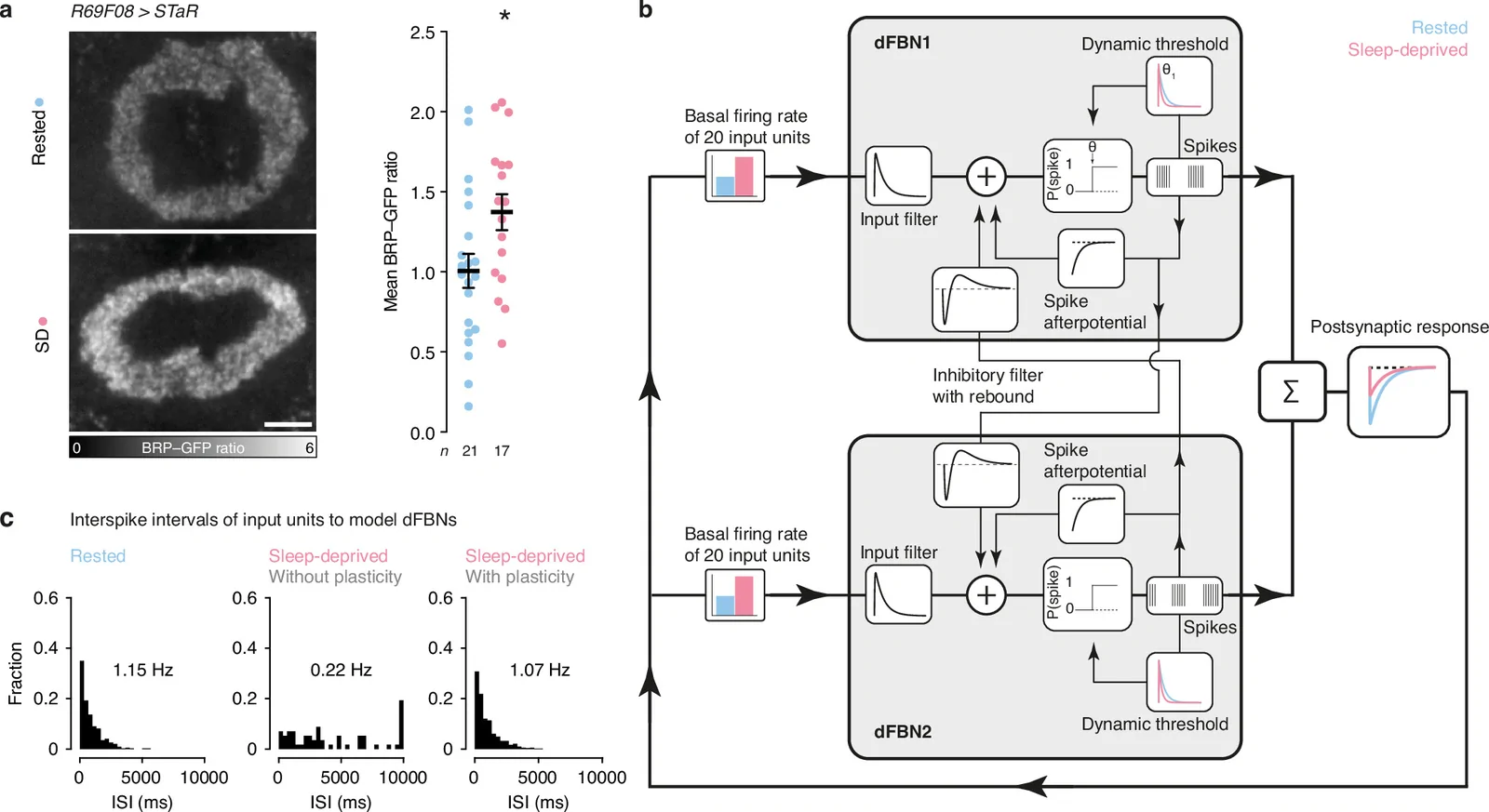
Efferent dFBN synapses depress after sleep deprivation. **a**, Summed-intensity projections of V5-tagged endogenous BRP in R5 neuron axons coexpressing mCD8::GFP. Emission ratios are intensity coded according to the key at the bottom and increase after sleep deprivation (SD) (*P* = 0.0236, two-sided *t*-test). **b**, Model architecture. dFBN1 and dFBN2 are each driven by 20 presynaptic units whose spike trains are inhomogeneous Poisson processes; the rates of these Poisson processes are subject to feedback inhibition by the summed spikes of both dFBNs. The membrane potential of each dFBN is a linear sum of resting potential, excitatory postsynaptic potentials due to Poisson units (input filter), inhibitory postsynaptic potentials and postinhibitory rebound due to the contralateral dFBN (inhibitory filter with rebound), and spike afterpotential. The membrane potential passes through a dynamic threshold to produce spikes. Color indicates sleep history-dependent variations in the basal firing rates of Poisson units, the dynamic threshold of dFBNs and the postsynaptic response to transmission from dFBN synapses. **c**, Interspike interval (ISI) distributions of Poisson units at baseline and after sleep deprivation, in the absence and presence of plastic feedback connections from dFBNs. Data are means ± s.e.m.; the asterisk indicates a significant difference (*P* < 0.05) in a planned pairwise comparison. Scale bar, 10 μm. For statistical details see Supplementary Table 3.

In contrast to SWA in mammals, which can be recorded across the entire cortical surface during NREM sleep^8^, dFBN slow waves are products of a local circuit with features of a central pattern generator. Despite their apparent simplicity, pattern generators are notorious for the sensitivity of their dynamics to modulatory changes in biophysical parameters^39–41^. dFBNs experience many such changes as sleep pressure builds: they increase their gain^2^, modulate voltage-gated and leak potassium conductances in opposite directions^6^, and receive heightened (indirect) excitatory drive from R5 neurons^42^. Single-cell transcriptomic data^4^ suggest that these changes, which all enhance activity, are counterbalanced by a weakening of efferent dFBN synapses. Sleep deprivation was found to selectively downregulate gene products with roles in synapse assembly, active zone maintenance, synaptic vesicle release and presynaptic homeostatic plasticity^4,43^. Prominent among these differentially expressed genes was *bruchpilot* (*brp*)^4^, which encodes a structural component of active zones^44,45^. Tagging the endogenous BRP protein with a V5 peptide through dFBN-restricted genomic recombination^46^ allowed us to quantify its abundance as a function of sleep history. In a clear reflection of the transcriptional picture^4^, the intensity of BRP::V5 fluorescence (normalized to that of coexpressed mCD8::GFP), an index of active zone size and a structural correlate of synaptic strength^47^, decreased at dFBN synapses of sleep-deprived flies (Fig. 8a). This is an unusual adjustment: waking experience, whether enforced by mechanical agitation or enriched by social interaction, is widely thought to lead to synaptic potentiation^48^, and axon terminal numbers or BRP levels indeed increase in many parts of the sleep-deprived *Drosophila* brain^49,50^, including virtually all specific neuron types that have been examined, such as R5 neurons^42^ (Extended Data Fig. 10a), Kenyon cells^51^ and clock neurons^49^. The anticyclical depression of dFBN synapses after sleep loss thus underlines, like their anticyclical energy metabolism does^4^, their special status with respect to sleep.

For a functional confirmation under activity-normalized conditions that sleep deprivation attenuated synaptic transmission from dFBNs, we measured the optically evoked axonal release of glutamate from dFBNs coexpressing CsChrimson and iGluSnFR. Saturation hyperbolas described the dependence of transmitter secretion on light intensity irrespective of sleep history, but the iGluSnFR signal saturated at lower levels in sleep-deprived than in rested flies (Fig. 8b). The significance of presynaptic depression, which we have now demonstrated at the transcriptional^4^, structural and functional levels, for the sleep need-dependent modulation of SWA becomes clear in a simple model where two dFBNs are embedded in recurrent circuitry^20,42^ and connected across the midline (Fig. 8c and Extended Data Fig. 10b). These direct, symmetric, and inhibitory connections are necessary (and must be sufficiently strong) to produce rhythmic, pacemaker-like SWA. Empirically, individual dFBNs become more excitable as sleep pressure rises^2^; artificially enhancing this excitability promotes sleep^3–6^; SWA power grows with sleep drive (Fig. 6c and Extended Data Fig. 2e) and optogenetic replay of SWA induces sleep (Fig. 4c–e). However, the network’s recurrent organization allows an increase in SWA following changes in intrinsic dFBN excitability and external excitatory drive only if a homeostatic weakening of dFBN synapses is part of the simulations (Fig. 8d); without it, dFBNs would smother their own sources of excitation (Extended Data Fig. 10c). The two seemingly opposing forces observed in dFBNs after sleep deprivation—increased excitability and decreased synaptic output—may in reality work together to generate an oscillatory representation of sleep need.

## Discussion

Our work, along with previous studies^32–35^, has uncovered remarkable parallels and striking differences in the sleep-related neuronal dynamics of flies and mammals. Slow waves reflecting alternating UP and DOWN states are a common marker of sleep pressure, but the biophysical origin, spatial spread and temporal persistence of these waves differ. A central pattern generator with rhythmic dFBNs at its core encodes sleep need in the amplitude of its periodic output, which oscillates robustly also in awake flies. It is this stability, and the considerable lengths dFBNs go to to maintain it at variable levels of sleep pressure, that poses the most intriguing questions.

dFBNs keep a record of sleep need in a biochemical memory where each unit of storage is a Shaker–Hyperkinetic channel whose cofactor oxidation state holds one bit of information^5^. Productive encounters with PUFA-derived carbonyls increase, whereas depolarization-controlled exchange reactions decrease, the fraction of channels with oxidized cofactors^5^. The fill level of the integrator thus depends on a dynamic equilibrium between oxidation and exchange. Regular spikes would tip this equilibrium toward exchange and thereby introduce a leak into the integrator, extending its time constant. We currently do not know how rapidly the Hyperkinetic pool reaches capacity, but the addition of a spike-triggered leak offers flexibility to adjust the time to saturation. With a range of spiking patterns in the dFBN population, which is likely to include forms of coordinated activity that have remained undetected by Ca^2+^ imaging, the same mechanism could, in principle, produce a spectrum of integration times and the characteristic heavy-tailed distribution of sleep bout durations.

dFBNs have a demonstrated ability to switch reversibly between long-lasting electrically active ON and quiescent OFF states^6^. These binary states (which were discovered under conditions where transmembrane currents were clamped^6^) bracket the extremes of the dynamic range used by dFBNs to map largely cell-autonomous, sleep history-dependent metabolic variables, such as mitochondrial electron leakage^3,4^ and levels of peroxidized lipids^5^, onto electrical signals that can be communicated to other cells. Under unclamped conditions, we now understand, this dynamic range is set so that an oscillatory representation of sleep pressure continuously flows to downstream structures, including during wakefulness. Only manipulations that forcibly block the discharge of sleep pressure appear to interrupt this periodic signal—at a price that is debited biochemically^3–5^ and repaid in heightened SWA power when inhibition is released. dFBNs fall silent during applications of exogenous dopamine^6^ and reduce their activity below baseline during mechanical sleep deprivation^52^; conversely, artificial depolarization of dFBNs during enforced waking prevents the mitochondrial rearrangements that are a visible token of the associated metabolic cost^4^. As sleep and energy homeostasis are so tightly interwoven^4,53^, overlapping neuronal populations—dFBNs in flies^54,55^ and hypothalamic neurons expressing agouti-related protein, pro-opiomelanocortin or hypocretin/orexin in mammals^56–59^—may serve double duty in coordinating both.

The utility of communicating an unbroken record of sleep pressure to the action-selection circuitry of the fan-shaped body^16^ is obvious: the likely need for future sleep is an important factor in choosing what to do now. However, if the same periodic signal also controls transitions between sleep–wake states, as our replay and interference experiments confirm, continuous variations in SWA power must be transformed into binary outcomes. This analog-to-digital conversion could take several forms. For example, dFBNs could target the central complex through strongly facilitating synapses that selectively transmit^60^ the high-frequency, large-amplitude somnogenic bursts we observe. Alternatively, because action selection is an inherently competitive winner-take-all process, it may be sufficient for dFBNs to alter the probability that inaction is favored among competing options and let the internal architecture of the fan-shaped body^16^ secure the exclusivity of the choice.

## Methods

### *Drosophila* strains and culture

Flies were grown on media of cornmeal (62.5 g l^−1^), inactive yeast powder (25 g l^−1^), agar (6.75 g l^−1^), molasses (37.5 ml l^−1^), propionic acid (4.2 ml l^−1^), tegosept (1.4 g l^−1^) and ethanol (7 ml l^−1^) under a 12-h light:12-h dark cycle at 25 °C in ~60% relative humidity, unless stated otherwise. To prevent the undesired activation of optogenetic actuators or the isomerization of all-*trans* retinal by ambient light, flies expressing CsChrimson and their controls were reared and housed in constant darkness and transferred to food supplemented with 2 mM all-*trans* retinal (Molekula or Sigma) in DMSO, or to DMSO vehicle only, 2 days before the optical stimulation experiments, at an age of 1–3 days post eclosion. Carriers of the *hsFLP* transgene^61^ were cultivated at 18 °C and placed on retinal-supplemented food for 5 days before optogenetic stimulation experiments; carriers of the *tub-GAL80*^*ts*^ or *UAS-TrpA1* transgenes^62,63^ and their controls were raised at 18 °C or 21 °C, respectively.

Driver lines^64,65^ *R23E10-GAL4* and *R23E10*-*LexA*; the repressor lines *VGlut-3xGAL80* (ref. ^66^), *teashirt-GAL80* (ref. ^67^) and *tubulin-GAL80*^*ts*^ (ref. ^62^); and the split-GAL4 hemidrivers^66,68–70^ *R23E10-DBD*, *VGlut-p65AD*, *R84C10-p65AD* and *Gad1-p65AD* were used in the indicated combinations to target dFBNs or other constituents of the *R23E10-GAL4* pattern; *R69F08-GAL4* labeled R5 ring neurons of the ellipsoid body^42,64^. Effector transgenes encoded fluorescent markers for neuroanatomy (*UAS-6xEGFP*^71^, *UAS-mCD8::GFP*^72^, *lexAop-GFP*^73^, *UAS-mCD4::tdTomato*^74^ and *UAS-myrRFP*) or the labeling of synaptic active zones^46^ (*lexAop-GFP*;*UAS-STaR*); the Ca^2+^ sensor^75,76^ GCaMP6f; the glutamate^77^, acetylcholine^78,79^ or dopamine^80^ sensors iGluSnFR(A184V), GRAB_ACh_, iAChSnFr or GRAB_DA2m_, respectively; the opto- or thermogenetic actuators^28,81^ CsChrimson^82^ or TrpA1 (ref. ^63^); cassettes for the mutually exclusive^24^ expression of green fluorescence (CD8::GFP or GCaMP6f) or CsChrimson::tdTomato, or of ECFP::VAChT and EGFP::VGlut fusion proteins^23^, after the FLP-mediated excision^61,83^ of a transcriptional terminator; the low voltage-activated Shaker derivative^84^ EKO or the inwardly rectifying potassium channel^43,85^ Kir2.1; or RNAi constructs for interference with the expression of *VGlut* (four independent transgenes^86,87^). Supplementary Table 4 lists all fly strains and their sources.

### Sleep measurements, sleep deprivation and optogenetic induction of sleep

Females aged 2–4 days were individually inserted into 65-mm glass tubes containing food reservoirs, loaded into the Trikinetics *Drosophila* Activity Monitor system, and housed under 12-h light:12-h dark conditions at 25 °C in ~60% relative humidity. Flies were allowed to habituate for 1 day before sleep—classified^88,89^ as periods of inactivity lasting >5 min and averaged over two consecutive recording days (Extended Data Fig. 7)—and the probabilities of initiating or terminating movement^90^ (P(Wake) and P(Doze), respectively) were quantified with the help of the Sleep and Circadian Analysis MATLAB Program^91^. Immobile flies (<2 beam breaks per 24 h) were manually excluded. Flies carrying *tub-GAL80*^*ts*^ transgenes and their controls were transferred to 31 °C for 2 days before sleep measurements, also at 31 °C, or kept at 18 °C throughout.

Our standard method of sleep deprivation used the sleep-nullifying apparatus^92^ (SNAP): a spring-loaded platform stacked with Trikinetics monitors was slowly tilted by an electric motor, released and allowed to snap back to its original position. The mechanical cycles lasted 10 s and were repeated continuously. Alternatively, as noted, an Ohaus Vortex Mixer stacked with Trikinetics monitors produced horizontal circular motion stimuli with a radius of ~1 cm at 25 Hz for 2 s; stimulation periods were randomly normally distributed within 20-s bins. Unless otherwise stated, sleep deprivation lasted for 12 h, beginning at ZT 12. The two methods produced comparable levels of sleep loss and rebound (Fig. 5b,d and Extended Data Figs. 6 and 7) without impairing postdeprivation locomotion (Extended Data Fig. 7f). A third, nonmechanical method of sleep deprivation, using flashing light stimuli, is described below.

Rebound sleep was measured in the 24-h window after mechanical deprivation. A cumulative sleep loss plot was calculated for each individual by comparing the percentage of sleep lost during overnight sleep deprivation to the immediately preceding unperturbed night. The percentage of individual sleep lost was normalized to the maximal sleep loss for each genotype. Individual sleep regained was quantified by normalizing the difference in sleep amount between the rebound and baseline days to baseline sleep. Only flies losing >95% of baseline sleep were included in the analysis.

In photostimulation experiments, female flies expressing CsChrimson in dFBNs were individually inserted into 65-mm glass tubes and loaded into a custom array^20^ of light-tight chambers with high-power 630-nm LEDs (Multicomp OSW-4388). The apparatus was operated in a temperature-controlled incubator (Sanyo MIR-154) at 25 °C. For movement tracking, the chambers were continuously illuminated from below using low-power infrared (850 nm) LEDs and imaged from above with a high-resolution CMOS camera (Thorlabs DCC1545M) equipped with a long-pass filter (Thorlabs, FL850-10) to reject stimulation light pulses, which lasted 3 ms and delivered ~28 mW cm^−2^ of optical power. A virtual instrument written in LabVIEW (National Instruments) extracted real-time position data from video images. Periods of inactivity lasting ≥5 min were classified as sleep. If flies were found dead at the end of the 2-day experiment, data from the 30-min time bin preceding the onset of continuous immobility (≥29.5 min per 30-min bin for ≥2 h until the end of the experiment) onward were excluded; if the period of continuous immobility began during or before optical stimulation, all data from that individual were discarded. The 29.5-min threshold was applied to avoid scoring rare instances of video tracker noise as movement.

To rouse flies during optogenetic stimulation of dFBNs (Fig. 4c), a 385-nm LED (M385L3, ThorLabs) controlled by a dimmable LED driver (LEDD1B, ThorLabs) delivered ~1–3 mW cm^−2^ of optical power continuously for 3 min, followed by 3 min of darkness. Cycles lasted for 1 h, beginning at ZT 13.5.

The same device was used for visual sleep deprivation (Extended Data Fig. 6g,h). Flies were kept under a 12-h light:12-h dark cycle and exposed for 12 h overnight to arousing 385-nm light, which was delivered as a series of Poisson-distributed epochs (*λ* = 24.3 epochs per hour); the spacing and duration of these epochs was truncated to the range of 2–4 min. Visual stimuli changed over the course of the night from continuous illumination (ZT 12–15) to flicker frequencies drawn from uniform probability distributions with ranges of 1–100 Hz (ZT 15–18), 5–40 Hz (ZT 18–21) and 15–17 Hz (ZT 21–24); the duty cycle of each stimulation block was randomly chosen to fall between 5% and 95%. Residual sleep during visual stimulation was compared to the preceding unperturbed night; sleep regained during the subsequent 12-h daytime period was quantified relative to the preceding 12-h day and normalized to the amount of sleep lost during visual deprivation. Only flies losing >30% of baseline sleep were included in the analysis.

### Two-photon imaging

Females aged 1–3 days were implanted with chronic imaging windows^93,94^ 2–3 days before the experiment. Unless otherwise stated (Supplementary Table 1), surgical openings created by removing cuticle, adipose tissue and trachea were sealed with a thin layer of translucent UV-curable epoxy glue (Norland NOA13825; Opticure 2000 UV lamp, Norland) in a custom chamber perfused with CO_2_ to avoid O_2_ inhibition of the curing process, which lasted <45 s. The adhesive stabilized the brain, had a refractive index matched to that of water and lacked any detectable adverse effects—including effects on sleep—after flies had recovered from anesthesia (Extended Data Fig. 1a,b). Flies were housed singly for 2–3 days after the procedure, cold-anesthetized, head-fixed to a custom mount with eicosane (Sigma), allowed to recover for ≥20 min and imaged on a Movable Objective Microscope with resonant scanners (Sutter Instruments) controlled through ScanImage 5.4.0 software (Vidrio Technologies) running on MATLAB2015a (The MathWorks). For sleep deprivation before imaging, experimental flies and their controls were placed into Trikinetics monitors 1–2 days after the implantation of the window.

Only healthy flies in which the adhesive effectively suppressed brain movements were imaged. Fluorophores were excited by a Mai Tai DeepSee Ti:sapphire laser (Spectra Physics model eHP DS; center wavelength of 930 nm). A Pockels cell (302RM, Conoptics) kept the power at the specimen <15 mW (ThorLabs PM100D power meter console with ThorLabs S370C sensor head). Emitted photons were collected by a ×20 (1.0 NA) water immersion objective (W-Plan-Apochromat, Zeiss), split into green and red channels by a dichromatic mirror (Chroma 565dcxr) and bandpass filters (Chroma ET525/70m-2p and Chroma ET605/70m, respectively) and detected by GaAsP photomultiplier tubes (H10770PA-40 SEL, Hamamatsu Photonics). If simultaneous optogenetic stimulation was performed, an alternative bandpass filter (Semrock BrightLine FF01-520/60-25) was present in the green emission path. Photocurrents were passed through high-speed amplifiers (HCA-4M-500K-C, Laser Components) and integrators (BLP-21.4+, Mini-Circuits) to maximize the signal-to-noise ratio. The objective was mounted on a MIPOS piezo actuator (PiezoSystemJena) controlled through ScanImage. Supplementary Table 1 lists the number of imaging planes, their axial distances, numbers of pixels, acquisition rates and additional parameters for each experiment.

For optogenetic stimulation, a 625-nm LED (M625L3, ThorLabs) controlled by a dimmable LED driver (LEDD1B, ThorLabs) delivered ~0.5–25 mW cm^−2^ of optical power through a bandpass filter (Semrock BrightLine FF01-647/57-25) to the head of the fly. The voltage steps controlling the LED driver were recorded in a separate imaging channel for post hoc alignment. Imaging of iGluSnFR, iAChSnFR and GRAB_ACh_ during optogenetic stimulation of dFBNs in Fig. 3d,e was performed in the presence of 1 µM TTX.

To deliver arousing stimuli, an 808-nm laser diode (Thorlabs L808P500MM) was mounted on a temperature-controlled heat sink (ThorLabs TCDLM9 with ThorLabs TED200C controller) and aimed at the abdomen of a fly standing or walking on a 6-mm polystyrene ball supported by a stream of air (0.3 l min^−1^). The diode was restricted to a maximal output of 50 mW by a ThorLabs LDC210C laser diode controller. Rotations of the spherical treadmill were recorded by a GuppyPro camera equipped with a magnification lens and a Semrock FF01-647/57-50 bandpass filter at a mean acquisition rate of 22.80 Hz. The treadmill was dimly illuminated by a red LED through a bandpass filter (Semrock BrightLine FF01-647/57-25). Forward and turning velocities were computed by a custom MATLAB script^95^. Movement and imaging traces were aligned post hoc using simultaneously recorded voltage steps as time stamps.

For focal transmitter applications, patch pipettes (~10 MΩ) containing 10 mM dopamine, 30 mM acetylcholine or 2 mM L-glutamate in extracellular solution were positioned near the dendritic (in the case of dopamine and glutamate) or axonal (in the case of acetylcholine) arborizations of dFBNs^6^. A TTL-controlled voltage step triggered the delivery of transmitter by a PDES-02DX pneumatic drug ejection system (npi electronic GmbH). Voltage steps were recorded in ScanImage for post hoc alignment. Movement artifacts were controlled for by titrating the ejection pressure to 10–25 kPa and recording mCD4::tdTomato fluorescence in a separate imaging channel. In the case of acetylcholine, the pulse duration was 40 ms, close to the minimal valve switching time.

Functional imaging data were analyzed using custom code in MATLAB2024a. Rectangular regions of interest (ROIs) containing dFBN processes or irregularly shaped ROIs following the contours of dFBN somata or axons were manually drawn, along with a background ROI positioned on a nonfluorescent brain area, and time series of mean fluorescence intensities were extracted from two-photon image stacks. After subtraction of the average intensity of the background time series, ∆*F*/*F* curves were computed as ∆*F*_*t*_/*F*^0^_*t*_ = (*F*_*t*_ – *F*^0^_*t*_)/*F*^0^_*t*_, where *F*_*t*_ is the raw fluorescence intensity in frame *t*. For event-aligned analyses (that is, GCaMP transient onset, optogenetic stimulation and arousing heat), *F*^0^_*t*_ was the mean fluorescence in a given time window before the event. To correct for motion during pharmacological dopamine applications, normalized changes in green-to-red ratios (∆*R*/*R*) were computed as ∆*R*_*t*_/*R*_0_ = (*R*_*t*_ – *R*_0_)/*R*_0_, where *R*_*t*_ is the element-wise ratio between background-subtracted GCaMP and mCD4::tdTomato signals and *R*_0_ is the mean ratio in the 40 s before dopamine application.

For measurements of spontaneous dFBN activity in flies expressing *R23E10-GAL4*-driven GCaMP, the time-varying baseline fluorescence *F*^0^_*t*_ was obtained as the tenth percentile of a symmetric sliding window of *F*_*t*_, spanning 501 frames (~34 s). The resulting ∆*F*/*F* traces were smoothed with an 8-element Gaussian sliding window. GCaMP transients were detected as peaks in the low-pass filtered (15-element Gaussian sliding window) time derivative of the ∆*F*/*F* trace; the amplitudes of transients were defined as the peak within the 15 frames following the maximal rise of ∆*F*/*F*. Interhemispheric crosscorrelations were computed on the normalized time derivative of the traces, which eliminates slow trends in the signal but captures the time course of dFBN bursts (Fig. 1f). For the calculation of coherence spectra (Extended Data Fig. 2b), time series were windowed into 500-element-long segments overlapping by 50%.

For measurements of dFBN activity in flies expressing GCaMP under the control of *R23E10* ∩ *R84C10-GAL4*, the time-varying baseline fluorescence *F*^0^_*t*_ was obtained as the tenth percentile of a symmetric sliding window of *F*_*t*_, spanning 1,001 frames (~30 s) due to the higher frame rate relative to experiments using *R23E10-GAL4*-driven GCaMP. No smoothing of the fluorescence time series was performed. Differences in the shapes and magnitudes of power spectra in Figs. 1a, 4b, 5e, 6c and 7e and Extended Data Figs. 2e and 9d reflect differences in image acquisition settings, which were tailored to each experiment and can be found in Supplementary Table 1.

The blinded extraction of ROIs required some exclusions because of low signal-to-noise ratios. ROIs were excluded automatically from further analysis if their background-subtracted mean GCaMP fluorescence failed to exceed the standard deviation of a comparable background signal by 20-fold (raw traces) or 45-fold (smoothed traces). The baseline fluorescence of most ROIs easily passed these thresholds, with mean GCaMP fluorescence exceeding the standard deviation of background noise by factors of 89 (raw traces) or 186 (smoothed traces). In addition, where several focal planes of the same hemisphere were imaged, signals were screened for multiple representations of the same cell(s) by computing pairwise correlations between ROIs at different focal depths. Only the ROI showing the largest fluorescence intensity was retained if the correlation coefficient exceeded 0.85 for dendritic ROIs and 0.5 for somatic ROIs; in the latter case we confirmed by visual inspection that redundant ROIs covered the same somata. In GRAB_DA2m_ experiments, the ROI displaying the highest response to the first heat shock was selected automatically for further analysis.

To relate GCaMP signals recorded simultaneously in somata and dendrites (Extended Data Fig. 1c–g) or dendrites and axons (Extended Data Fig. 1h–j), linear regression models using the somatic or dendritic ∆*F*/*F* traces as predictor variables of the dendritic or axonal traces, respectively, were fit in MATLAB. Model performance was evaluated by fivefold crossvalidation.

As Ca^2+^ diffuses into dFBN somata through the long, thin necks of dendrites and axons, somatic GCaMP signals are temporally low-pass filtered versions of signals recorded in the neuropil. This made an exhaustive characterization and classification of activity patterns of the dFBN ensemble (whose members could only be resolved in cell body images) impossible. Our analysis therefore focused on the distinction of pacemaker-like activity, which was readily detectable, from all other forms of activity. ∆*F*/*F* traces of individual somata were used to compute the peak signal power between 0.2 and 2 Hz; a prominent periodicity in the autocorrelogram with a wide amplitude swing during the first period was diagnostic of a slow rhythm (Fig. 1c).

### Electrophysiology

Female flies were head-fixed to a custom mount with eicosane (Sigma) or soft thermoplastic wax (Agar Scientific). A surgical window was cut into the cuticle; adipose tissue, trachea and the perineural sheath were removed and the brain was superfused with carbogenated extracellular solution (95% O_2_–5% CO_2_, pH 7.3, 275 mOsm) containing (in mM) 103 NaCl, 3 KCl, 5 TES, 8 trehalose, 10 glucose, 7 sucrose, 26 NaHCO_3_, 1 NaH_2_PO_4_, 1.5 CaCl_2_ and 4 MgCl_2_. The GFP- or GCaMP- and mCD4::tdTomato-labeled somata of dFBNs were visualized using ×20 (1.0 NA), ×40 (0.8 NA) or ×60 (1.0 NA) water immersion objectives (W-Plan-Apochromat, Zeiss, or LUMPLFLN40XW or LUMPLFLN60XW, Olympus) and targeted with borosilicate glass electrodes (8–11 MΩ) filled with internal solution (pH 7.3, 265 mOsM) containing (in mM) 10 HEPES, 140 potassium aspartate, 1 KCl, 4 MgATP, 0.5 Na_3_GTP and 1 EGTA. Signals were acquired at room temperature in current-clamp mode with a MultiClamp 700B amplifier (Molecular Devices), low-pass filtered at 5–20 kHz and sampled at 10–50 kHz using an Axon Digidata 1440 digitizer controlled through pCLAMP 10.5 (Molecular Devices) or a Power1401-3A data acquisition interface controlled through Signal (Cambridge Electronic Design).

For simultaneous whole-cell patch-clamp recording and two-photon imaging, a TTL-gated voltage signal was recorded in pCLAMP and ScanImage for the post hoc alignment of electrophysiology and imaging data. Simultaneously recorded membrane voltages were compared to GCaMP fluorescence time series at the soma (nine of ten cells); in one cell, Ca^2+^ signals were imaged in dendrites. The ∆*F*/*F* curve was computed as ∆*F*_*t*_/*F*^0^_t_ = (*F*_*t*_ – *F*^0^_*t*_)/*F*^0^_*t*_, where *F*_*t*_ is the raw fluorescence trace and the time-varying baseline fluorescence *F*^0^_*t*_ was obtained as a 300-element (~5 s) moving average of *F*_*t*_. The imaging time series was *z*-score-normalized and linearly interpolated and patch-clamp recordings were downsampled, so that both signals had the same temporal resolution of 2 kHz; the resulting traces were aligned to the TTL time stamp. The momentary firing rate (or peri-event time histogram) was computed in 100-ms bins and used to create a predictor matrix for the estimation of the GCaMP impulse response from the interpolated and aligned imaging trace, which was smoothed using a 150-ms moving average and then downsampled to the same frame rate as the predictor matrix (10 Hz). The resolution of 10 Hz was chosen to control for zero inflation in the predictor matrix (that is, the number of time bins without spikes in the peri-event time histogram) and reduce the number of coefficient weights to be fitted to 20 for a 2-s long impulse response. Model performance was evaluated by estimating the individual GCaMP impulse responses of all ten cells by linear regression and predicting the Ca^2+^ signal of each dFBN from its spiking record, using the normalized impulse response derived from the other nine cells (leave-one-out crossvalidation). Coefficients of determination (*R*^2^) were obtained from the squared maximum crosscorrelations between normalized and predicted imaging data.

In studies of inter-dFBN connectivity, low basal expression of the *hsFLP* transgene, without additional heat shock, was sufficient to produce recombination events^24,61^ whose visible sign was a mosaic of green and red fluorescent dFBNs (Extended Data Fig. 4i). The presence of both dFBN populations was confirmed by live microscopy during each experiment. CsChrimson-expressing cells were stimulated with a 630-nm LED (Multicomp OSW-4388) controlled by a TTL-triggered dimmable LED driver (Recom RCD-24-0.70/W/X3) and focused on the fly’s head with a mounted 60-mm lens (Thorlabs). The light source delivered 11–80 mW cm^−2^ of optical power. Where indicated, 1 μM TTX was perfused into the bath to probe for monosynaptic connections^24,96^. In experiments with TrpA1, temperature ramps were generated by a TC-324C controller with SH-27B in-line solution heater (Warner Instruments). Data were analyzed with custom procedures, using the NeuroMatic v3 package^97^ (http://neuromatic.thinkrandom.com) in Igor Pro 8.04 (WaveMetrics).

### Confocal imaging

Single-housed females aged 6 days post eclosion were dissected at ZT 0. For the quantification of BRP::V5, experimental and control samples were processed in parallel, following ad libitum sleep or 12 h of sleep deprivation. Nervous systems were fixed for 20 min in 0.3% (v/v) Triton X-100 in PBS (PBST) with 4% (w/v) paraformaldehyde, washed five times with PBST and incubated sequentially at 4 °C in blocking solution (10% goat serum in 0.3% PBST) overnight, with primary antibodies in blocking solution for 2–3 days, and with secondary antibodies in blocking solution for 2 days. The primary antibodies included mouse nc82 anti-BRP (1:10, Developmental Studies Hybridoma Bank), mouse anti-V5 (1:400, Thermo Fisher), chicken anti-GFP (1:1,000 or 1:500 for STaR, AbCam) and mouse anti-GFP (1:50 for GCaMP, Thermo Fisher); the secondary antibodies were goat anti-Mouse Alexa Fluor 488 (1:1000, Thermo Fisher), goat anti-Chicken Alexa Fluor 488 (1:1,000 or 1:500 for STaR, Thermo Fisher) and goat anti-Mouse Alexa Fluor 633 (1:500, Thermo Fisher). The samples were washed five times with PBST before and after the addition of secondary antibodies, mounted in Vectashield and imaged on a Leica TCS SP5 confocal microscope (Leica Application Suite AF 2.7.3.9723) with an HCX IRAPO L ×25 (0.95 NA) water immersion objective. Only anatomically intact specimens from live flies (at the point of dissection) were analyzed.

For evaluating possible anatomical anomalies after interference with the expression of VGlut in dFBNs, brains expressing *R23E10* *∩* *VGlut-GAL4*-driven GFP and *VGlut*^RNAi^ or *RFP* transgenes were counterstained for BRP, imaged and analyzed with a semi-automated custom script in Fiji. The BRP signal was used to define the dFB volume manually. GFP fluorescence was quantified in summed *z* stacks containing this volume, following the subtraction of average background fluorescence in a nearby area. To estimate the density of dFBN projections into this volume (that is, the innervation density), *z* slices were passed through a Gaussian low-pass filter (0.5 σ) and manually thresholded to exclude isolated low-intensity pixels. GFP-positive volumes were identified and measured with the ‘3D Objects Counter’ function in Fiji (‘threshold’ 10, ‘minimum puncta size’ 100 voxels), summed and expressed as a percentage of the dFB volume.

Transporters mediating the uptake of acetylcholine (VAChT) and glutamate (VGlut) into synaptic vesicles were visualized in flies carrying the fluorescent protein fusion alleles^23^ *ECFP::VAChT* or *EGFP::VGlut*. Expression of these fusion alleles—which retain the regulatory context of the endogenous genes—was conditional on the FLP-mediated excision of an *Frt*-flanked stop codon, located downstream of the respective gene’s endogenous start codon (*Frt-stop-Frt-EGFP::VGlut* or *Frt-stop-Frt-ECFP::VAChT*). Brains were imaged on a Zeiss LSM980 microscope (ZEN blue 3.3) with a Plan-Apochromat ×20 (0.8 NA) objective, an excitation wavelength of 488 nm for EGFP and 433 nm for ECFP, and emission bands of 489–657 nm and 454–560 nm, respectively.

### Connectome analysis

The hemibrain:v1.2.1 connectome^98^ and the FlyWire female adult fly brain dataset FAFB v783 (refs. ^17,99,100^) were accessed in neuPrint+ (ref. ^101^) and Codex (https://codex.flywire.ai), respectively. The total number of synapses per connection was used as measure of connection strength. Connectivity matrices take into account cells assigned to *R23E10-GAL4* in the hemibrain^16^ and cell types FB6E1, FB6E3, FB6E4 and FB7E2 in the FlyWire^100^ connectomes; the same classification was used for comparisons between and within cell types.

Connectivity matrices were computed and analyzed in MATLAB2024a. Omitting all diagonal entries (the number of autapses, which was uniformly zero), an index of network density was obtained as the number of observed edges (inter-dFBN connections) divided by the number of all possible edges in the graph. The original directed graph was compared to an undirected graph, which was created by averaging the weighted connectivity matrix with its transpose. Instances of unidirectional connections formed by one synapse were assigned a mean of 1 in the undirected graph. Nonzero entries above the diagonal of the undirected connectivity matrices were binned to create the histograms of Extended Data Fig. 3b,g. To avoid potential confounds arising from the heavy-tailed weight distribution and high network density, the reciprocity of inter-dFBN connections was quantified as a function of connection strength. To do so, the weighted connectivity matrices were binarized with thresholds s ranging from 0 to 100, resulting in series of Boolean matrices ${B}^{(s)}$. The reciprocity r(s) of connections with synapse number n>s was obtained as

1 $$r(s)=\frac{1}{m(s)}\sum _{{ij}}{B}_{{ij}}^{(s)}{B}_{{ji}}^{(s)},$$

where m(s) is the total number of connections (nonzero entries) in ${B}^{(s)}$. Connection symmetry (that is, the relative strength of reciprocal connections) was evaluated by comparing the weighted connectivity matrix A with its transpose ${A}^{T}$. The Pearson correlation coefficient (omitting diagonal entries) of A and ${A}^{T}$ approaches 1, while the difference between A and ${A}^{T}$ approaches zero for a perfectly symmetric matrix. The scaled distance *a* from the zero matrix was therefore used as a measure of asymmetry

2 $$a=\frac{{{\rm{||}}{A}^{T}-\,A{\rm{||}}}_{F}^{2}}{{{\rm{||}}A{\rm{||}}}_{F}^{2}},$$

where ${{||}{\rm{\cdot }}{||}}_{F}^{2}$ is the squared Frobenius norm (equivalent to the sum of squares of all entries) and ${{||A||}}_{F}^{2}$ is used for normalization. For null model comparisons (*n* = 100,000), all entries of A (and consequently ${A}^{T}$) were randomly permuted before computing a. Values close to the mean of the respective null models are a sign of asymmetric connectivity (see, for example, MBONs in Extended Data Fig. 3l).

To determine the likely identities of non-dFB central neurons labeled by *R23E10 ∩ VGlut-GAL4*, the *R23E10-GAL4* line was inspected in NeuronBridge^102,103^, and 60 slides containing non-dFBN cells with arborizations in the posterior lateral protocerebrum or dorsal brain were examined by Color Depth Search. For each slide, the 30 top-scoring cell IDs were retrieved and retained if the cells (1) were not dFBNs, (2) their somata were located in the midbrain, (3) the cell IDs were present in the hemibrain:v1.2.1 or FlyWire FAFB v783 libraries and (4) the cells had been assigned the neurotransmitter type ‘glutamate’ in the FlyWire connectome.

### Statistics and reproducibility

Imaging and behavioral data were analyzed in MATLAB2024a and Prism 10 (GraphPad). All null hypothesis tests were two-sided. To control for type I errors, *P* values were adjusted to achieve a joint *α* of 0.05 at each level in a hypothesis hierarchy; multiplicity-adjusted *P* values are reported in cases of multiple comparisons at one level. Group means were compared by *t-*tests, ANOVA, two-way repeated-measures ANOVA or mixed-effects models, as stated, followed by planned pairwise analyses using Holm-Šidák’s multiple comparisons test. Repeated-measures ANOVA and mixed-effect models used the Geisser–Greenhouse correction. Where the assumption of normality was violated (as indicated by D’Agostino–Pearson test), group means were compared by Mann–Whitney test or Kruskal–Wallis ANOVA, followed by Dunn’s multiple comparisons test, as indicated.

The investigators were blind to sleep history, ZT, the inclusion or exclusion of dietary retinal and/or genotype during the selection of ROIs and background regions in functional imaging experiments (Figs. 1a, 2, 3f, 5e, 6, 7 and 8b and Extended Data Figs. 1h–j, 2 and 9d), the analysis of dFBN projections after interference with glutamatergic transmission (Extended Data Fig. 9a) and STaR experiments (Fig. 8a and Extended Data Fig. 10a). Expression patterns were reproducible in a minimum of five brains per genotype; Fig. 3a–c and Extended Data Fig. 4 show representative examples. No statistical tests were used to predetermine sample sizes for functional imaging or electrophysiological experiments, which are similar to those reported in the field.

Sample sizes in behavioral experiments (typically *n* = 32 flies per genotype) were chosen to detect 2-h differences in daily sleep with a power of 0.9. All behavioral experiments were run at least three times, on different days and with different batches of flies. The figures show pooled data from all replicates.

### Modeling

We studied a model of two dFBNs (one in the left and one in the right hemisphere) with reciprocal inhibitory connections. Each dFBN was driven by 20 presynaptic units whose spike trains were inhomogeneous Poisson processes; the rates of these Poisson processes were subject to feedback modulation by the summed inhibitory output of both dFBNs. The architecture of the model is based on anatomical and functional evidence of recurrent connectivity between R5 neurons of the ellipsoid body (corresponding to the presynaptic units) and dFBNs^20,42^. R5 neurons provide indirect excitation to dFBNs^42^, whose inhibitory activity in turn diminishes (again via indirect connections^20^) the excitatory drive R5 neurons provide. Our minimal model recapitulates the basic feedback logic of the circuit but simplifies it by replacing indirect with direct connections and relying on a biphasic filter (rather than explicit conductances) to model hyperpolarization and hyperpolarization-induced escape.

Sleep loss-dependent changes were incorporated via three model parameters, as suggested by empirical data (Extended Data Fig. 10b). First, dFBNs in sleep-deprived flies increase their intrinsic excitability^2^. This was modeled as a shortening of the refractory period following a dFBN spike. Second, R5 neurons display higher firing rates and larger presynaptic active zones after sleep deprivation^42^ (Extended Data Fig. 10a). This was modeled as an increase in the rate of the Poisson units. Third, dFBN output synapses weaken after sleep loss (Fig. 8a,b). This was modeled as a decrease in the connection weight between dFBNs and the Poisson units.

A spike-response model^104^ described the membrane potential ${v}_{j}\left(t\right)$ of each dFBN in discrete 1-ms time steps as a linear sum of a resting membrane potential (${v}_{\mathrm{rest}}$) of –50 mV, excitatory postsynaptic potentials due to input from *n* = 20 Poisson units, spike after-hyperpolarization, and the inhibitory postsynaptic potentials and postinhibitory rebound elicited by the contralateral dFBN

3 $${v}_{j}\left(t\right)={v}_{\mathrm{rest}}+\sum _{i=1}^{n}\sum _{{t}_{i}\in {S}_{i}^{\mathrm{in}}}\varepsilon \left(t-{t}_{i}\right)+\sum _{{t}_{j}\in {S}_{j}^{\mathrm{dFB}}}\eta \left(t-{t}_{j}\right)+\sum _{{t}_{{k}}\in {S}_{k}^{\mathrm{dFB}}}{w}_{{jk}} \zeta \left(t-{t}_{k}\right).$$

*S*_*i*_^in^, *S*_*j*_^dFB^ and *S*_*k*_^dFB^ are sets of spike times (*t*_*i*_, *t*_*j*_ and *t*_*k*_) of the *i*th input unit, dFBN_*j*_, and the contralateral dFBN_*k*_.

Excitatory postsynaptic potentials had a rise time constant ${\tau }_{1}$ of 7 ms and a decay time constant ${\tau }_{2}$ of 150 ms; their amplitude ε was scaled to a peak of 0.75 mV

4 $$\varepsilon \left(t-{t}_{i}\right)=\exp \left(-\frac{t-{t}_{i}}{{\tau }_{1}}\right)-\exp \left(-\frac{t-{t}_{i}}{{\tau }_{2}}\right).$$

The post-spike hyperpolarization was defined as

5 $$\eta \left(t-{t}_{j}\right)={\eta }_{0} \exp \left(-\frac{t-{t}_{j}}{{\tau }_{3}}\right)$$

with ${\tau }_{3}$ = 300 ms and ${\eta }_{0}=$ –6 mV.

The time course of an inhibitory postsynaptic potential and its postinhibitory rebound was encapsulated by a biphasic filter

6 $$\zeta \left(t-{t}_{k}\right)=\exp \left(-\frac{t-{t}_{k}}{{\tau }_{4}}\right)-\rho \exp \left(-\frac{t-{t}_{k}}{{\tau }_{5}}\right)+\exp \left(-\frac{t-{t}_{k}}{{\tau }_{6}}\right),$$

with ${\tau }_{4}$ = 450 ms, ${\tau }_{5}$ = 225 ms, ${\tau }_{6}$ = 56 ms and ρ = 2. The filter was normalized and weighted by the amplitude ${w}_{{jk}}={w}_{{kj}}=$ 8 mV for each contralateral dFBN spike (equation (3)).

Spike initiation by dFBNs was deterministic. If a time-varying voltage threshold $\vartheta \left(t\right)$ was crossed, an action potential was initiated (modeled as the addition of a 20-mV impulse to the current threshold value). To incorporate refractoriness, this deterministic threshold was a function of spiking history

7 $$\vartheta \left(t\right)={\vartheta }_{0}+\sum _{{t}_{j}\in {S}_{j}^{\mathrm{dFB}}}\theta \left(t-{t}_{j}\right),$$

where the baseline spiking threshold ${\vartheta }_{0}$ was –45 mV and

8 $$\theta \left(t-{t}_{j}\right)={\theta }_{0} \exp \left(-\frac{t-{t}_{j}}{{\tau }_{7}}\right).$$

The decay time constant ${\tau }_{7}$ was 30 ms in rested and 15 ms in sleep-deprived conditions, respectively, to account for variation in the intrinsic excitability^2^ of dFBNs (Extended Data Fig. 10b). ${\theta }_{0}$ was 400 mV.

The spike trains of 20 input units per dFBN were modeled as inhomogeneous Poisson processes with a basal rate ${\omega }_{0}$ of 30 in rested and 60 in sleep-deprived flies (Extended Data Fig. 10b), matching empirical firing rate increases in R5 neurons^42^. Inhibitory feedback from the summed output of both dFBNs reduced the momentary Poisson rate $\omega \left(t\right)$ according to

9 $$\omega \left(t\right)=\psi \left(t-{t}_{j}\right)+\psi \left(t-{t}_{k}\right)+{\omega }_{0},$$

where

$$\psi \left(t-{t}_{j}\right)=-\exp \left(-\frac{t-{t}_{j}}{{\tau }_{8}}\right)+\exp \left(-\frac{t-{t}_{j}}{{\tau }_{9}}\right)$$

and

10 $$\psi \left(t-{t}_{k}\right)=-\exp \left(-\frac{t-{t}_{k}}{{\tau }_{8}}\right)+\exp \left(-\frac{t-{t}_{k}}{{\tau }_{9}}\right),$$

with ${\tau }_{8}$ = 7 ms and ${\tau }_{9}\,$= 1,000 ms. The amplitude of ψ was normalized and scaled by a variable output weight reflecting the observed presynaptic plastic changes in dFBNs; sleep deprivation was modeled as a 50% reduction in the postsynaptic response amplitude (Extended Data Fig. 10b). $\omega \left(t\right)$ was restricted to positive values.

Ca^2+^ imaging experiments were simulated by convolving the momentary firing rates of model dFBNs in 100-ms bins with the GCaMP impulse response function estimated from simultaneous patch-clamp recordings. Data were *z*-score normalized.

### Reporting summary

Further information on research design is available in the Nature Portfolio Reporting Summary linked to this article.

## Online content

Any methods, additional references, Nature Portfolio reporting summaries, source data, extended data, supplementary information, acknowledgements, peer review information; details of author contributions and competing interests; and statements of data and code availability are available at https://doi.org/10.1038/s41593-026-02431-9.

## Supplementary information

Supplementary Information Supplementary Tables 1–4.

Reporting Summary

Supplementary Video 1 Simultaneous two-photon imaging of GCaMP signals in dFBN dendrites of both hemispheres. The video, which is thresholded for display, was acquired at a frame rate of 14.56 Hz in a continuous imaging session lasting ~22 min.

Supplementary Video 2 Two-photon imaging of GCaMP signals in dFBN somata. The video, which is thresholded for display, was acquired at a frame rate of 14.56 Hz in an imaging session lasting 96 s.

Supplementary Video 3 Confocal image stack of the CNS of a fly expressing 6xEGFP under the control of *R23E10-GAL4* in the presence of the transcriptional repressor *VGlut-GAL80*. Green, 6xEGFP; blue, BRP. Scale bars, 100 μm.

Supplementary Video 4 Confocal image stack of the CNS of a fly expressing 6xEGFP under the control of hemidrivers *R23E10-DBD* and *VGlut-p65AD* (*R23E10 ∩ VGlut-GAL4*). Green, 6xEGFP; blue, BRP. Scale bars, 100 μm.

## Source data

Source Data Single file containing all statistical source data.

## Data Availability

Source data are provided with this paper and are available via GitHub at https://github.com/CNCBMiesenboeck/dFBNOscillator.

## Code availability

The minimal dFBN model described in Fig. 8 and Extended Data Fig. 10 can be run on CodeOcean^105^. MATLAB analysis code is available via GitHub at https://github.com/PeterHasenhuetl/dFBNOscillator.
