# Supplementary material for: A half-center oscillator regulates sleep pressure
Source: Nat Neurosci. 2026 Oct 15;29(11):2798–809. doi: 10.1038/s41593-026-02431-9 (PMC13724273; doi:10.1038/s41593-026-02431-9)
Supplement: Supplementary file 1 [file 41593_2026_2431_MOESM1_ESM.pdf]

nature neuroscience

Supplementary information

https://doi.org/10.1038/s41593-026-02431-9

A half-center oscillator regulates sleep pressure

In the format provided by the authors and unedited

Supplementary Table 1. Two-photon imaging parameters.

**Experiment and figures**	**N pixels (rows × columns)**	**Axial coordinates of focal planes (μm)†**	**Acquisition rate (Hz)¶**	**Sealed imaging window***	**Zeitgeber time**	**Additional details**
Dendritic GCaMP imaging (Fig. 1a, 2, 6, Extended Data Fig.1h–j, 2)	256 × 256	0, 10, 30, 40	14.56	Yes	0–4(+1); specified in Fig. 6.	ROI selection blind to sleep history and zeitgeber time. Data displayed in Fig.1a, 2, 6 Extended Data Fig. 1h–j and Extended Data Fig. 2 are from the same dataset. (20 μm instead of 40 in vortex experiments)
Cell body GCaMP imaging (Fig. 1b,c)	256 × 256	Four planes at variable distances of 0-30 μm	14.56	No	2–13	No between-group comparisons and thus no blinding during ROI selection. In some cases data were obtained from the left and right hemispheres of the same brain in separate imaging sessions. These were treated as independent experiments. dFBNs with a slow rhythm: 0–2 per hemisphere (median: 1).
Simultaneous cell body and dendritic GCaMP imaging (Extended Data Fig. 1c–g)	256 × 256	Four planes at variable distances of 0-40 μm	14.56	No	2–17	No between-group comparisons and thus no blinding during ROI selection. In some cases data were obtained from the left and right hemispheres of the same brain in separate imaging sessions. These were treated as independent experiments.
Simultaneous patch-clamp and GCaMP imaging (Fig. 1d–f)	256 × 256	0	58.25	No	3–12	No between-group comparisons and thus no blinding during ROI selection.
Dendritic GCaMP imaging plus VGlut RNAi in *R23E10* ∩ *R84C10* (Fig. 5e)	512 × 512	0	30.01	Yes	3–8	ROI selection blind to genotype.
Dendritic GCaMP imaging plus VGlut RNAi in *R23E10-GAL4* (Extended Data Fig. 9d,e)	256 × 256	0, 10, 20, 30	14.56	Yes	1–9	ROI selection blind to genotype.
iGluSnFR, iAChSnFR, and $GRAB_{ACh}$ imaging during optogenetic stimulation of dFBNs (Fig. 3d,e)	100 × 256	0, 10	68.32	No	4–11	No blinding during ROI selection. Stimulus-aligned traces are means of 20 repetitions per fly, applied every 6 s.
Dendritic GCaMP imaging during optogenetic stimulation of dFBNs (Fig. 4a)	100 × 256	0	136.63	No	3–13	No blinding during ROI selection. Stimulus-aligned traces are means of 20 repetitions per fly, applied every 6 s.
GCaMP imaging during optogenetic stimulation of a dFBN subset (Fig. 3f)	256 × 256	Two planes at distances of 7–13 μm	29.13	No	3–11	ROI selection blind to dietary retinal and genotype. Stimulus-aligned traces are means of several simultaneously recorded cell bodies per fly, averaged over 10 repetitions (light applied every 20 s; 1-2, 1-3, and 1-5 cell bodies in flies expressing CsChrimson::tdTomato with retinal, CsChrimson::tdTomato without retinal, and no CsChrimson::tdTomato, respectively. In some cases data were obtained from the left and right hemispheres of the same brain. These were recorded in separate imaging sessions and averaged post hoc. Average number of identified CsChrimson::tdTomato-positive cells per fly: 2.2 ±0.92 (mean ±standard deviation).
$GRAB_{DA2m}$ imaging during heat stimulation (Fig. 7b)	256 × 256	0, 10, 20, 30	14.56	Yes	1–10	No between-group comparisons and thus no blinding during ROI selection.
Dendritic GCaMP imaging during heat stimulation (Fig. 7c–e)	256 × 256	0, 10, 20, 30	14.56	Yes	3–11	No between-group comparisons and thus no blinding during ROI selection.
$GRAB_{DA2m}$ imaging during optogenetic stimulation of dFBNs (Fig. 7h)	256 × 256	0, 10, 20, 30	14.56	Yes	1–12	ROI selection blind to genotype.
iGluSnFR imaging during optogenetic stimulation of dFBNs after sleep deprivation (Fig. 8b)	100 × 256	0, 10	68.32	Yes	0–5	ROI selection blind to sleep history. Stimulus-aligned traces are means from two simultaneously recorded axonal ROIs (different focal planes) and 5 repetitions (for each light intensity) per fly. The order in which stimuli with different intensities were applied was varied between flies to avoid adaptation. Flies were placed on retinal food after

						optical window implantation and kept on retinal food during sleep measurements.
GCaMP or iAChSnFR imaging during focal application of dopamine (Extended Data Fig. 7c) or acetylcholine (Fig. 3e inset), respectively	256 × 256	Four planes at distances of 0-50 μm	14.56	No	4–10	No between-group comparisons and thus no blinding during ROI selection.

† In ascending order from ventral to dorsal.
¶ Volumetric rate when more than one focal plane was imaged.
* If no sealed imaging window was used, dissections were done on the recording day and brains were superfused with extracellular solution (see Methods).

Supplementary Table 2. Statistical analyses of Figures 3–8.

Figure	Statistical test	Pairwise comparison	Test statistic	*P*
**3f**	One-way ANOVA			
	Effect of genotype and retinal		$F_{2,27}$=43.66	<0.0001
	Holm-Šidák test	+ Retinal vs. *GCaMP* only	t_{27}=9.077	<0.0001
	Holm-Šidák test	+ Retinal vs. - Retinal	t_{27}=6.462	<0.0001
**3g**	Two-sided Wilcoxon test	Before vs. during	W = -45	0.0039
**4c (left)**	Two-way repeated-measures ANOVA			
	Time		$F_{20.81,1769}$ = 10.21	<0.0001
	Retinal		$F_{1,85}$ = 9.647	0.0026
	Time x retinal		$F_{45,3825}$ = 4.091	<0.0001
**4d**	Two-way repeated-measures ANOVA			
	Time		$F_{22.73,2205}$ = 11.23	<0.0001
	Retinal		$F_{1,97}$ = 19.95	<0.0001
	Time x retinal		$F_{22.73,2205}$ = 3.811	<0.0001
**4e (i)**	*R23E10 ∩ VGlut-GAL4>CsChrimson* vs. *CsChrimson* (burst stimulation)			
	Two-way ANOVA			
	Genotype		$F_{1,336}$ = 16.37	<0.0001
	Number of pulses		$F_{4,336}$ = 2.225	0.0666
	Genotype x number of pulses		$F_{4,336}$ = 0.8464	0.4965
	Burst vs. tonic stimulation (*R23E10 ∩ VGlut-GAL4>CsChrimson*)			
	Two-way ANOVA			
	Temporal structure		$F_{1,303}$ = 6.001	0.0149
	Number of pulses		$F_{4,303}$ = 2.898	0.0223
	Temporal structure x number of pulses		$F_{4,303}$ = 1.110	0.3517
**4e (ii)**	*R23E10 ∩ R84C10-GAL4>CsChrimson* vs. *CsChrimson* (burst stimulation)			
	Two-way ANOVA			
	Genotype		$F_{1,343}$ = 10.05	0.0013
	Number of pulses		$F_{4,343}$ = 4.246	0.0023
	Genotype x number of pulses		$F_{4,343}$ = 0.4740	0.7548
	Burst vs. tonic stimulation (*R23E10 ∩ R84C10-GAL4>CsChrimson*)			
	Two-way ANOVA			
	Temporal structure		$F_{1,369}$ = 6.758	0.0097
	Number of pulses		$F_{4,369}$ = 4.354	0.0019
	Temporal structure x number of pulses		$F_{4,369}$ = 0.9500	0.4350
**4e (iii)**	Burst vs. tonic stimulation (*R23E10 ∩ R84C10-GAL4>CsChrimson*)			
	Two-way ANOVA			
	Temporal structure		$F_{1,304}$ = 5.206	0.0232
	Number of pulses		$F_{3,304}$ = 8.133	<0.0001
	Temporal structure x number of pulses		$F_{3,304}$ = 3.559	0.0147
	Kruskal-Wallis ANOVA			
	Inter-burst frequency		H_4 = 51.23	<0.0001
	Dunn's test	0.1 Hz vs. 0.2 Hz	Z = 0.1933	>0.9999
	Dunn's test	0.1 Hz vs. 0.5 Hz	Z = 4.609	<0.0001
	Dunn's test	0.1 Hz vs. 1 Hz	Z = 5.960	<0.0001
	Dunn's test	0.2 Hz vs. 0.5 Hz	Z = 3.946	0.0005
	Dunn's test	0.2 Hz vs. 1 Hz	Z = 4.955	<0.0001
	Dunn's test	0.5 Hz vs. 1 Hz	Z = 0.5337	>0.9999
**5a**	Kruskal-Wallis ANOVA			
	Genotype		H_5 = 98.77	<0.0001
	Dunn's test	*R23E10 ∩ VGlut-GAL4>VGlut*$^{RNAi\ 104324KK}$ vs. *R23E10 ∩ VGlut-GAL4*	Z = 4.515	<0.0001
	Dunn's test	*R23E10 ∩ VGlut-GAL4>VGlut*$^{RNAi\ 104324KK}$ vs. *VGlut*$^{RNAi\ 104324KK}$	Z = 5.691	<0.0001
	Dunn's test	*R23E10 ∩ VGlut-GAL4>VGlut*$^{RNAi\ 2574GD}$ vs. *R23E10 ∩ VGlut-GAL4*	Z = 6.190	<0.0001
	Dunn's test	*R23E10 ∩ VGlut-GAL4>VGlut*$^{RNAi\ 2574GD}$ vs. *VGlut*$^{RNAi\ 2574GD}$	Z = 7.723	<0.0001
**5b (left)**	Two-way repeated-measures ANOVA			
	Time		$F_{1.556,275.5}$ = 36.70	<0.0001
	Genotype		$F_{2,177}$ = 3.693	0.0268
	Time x genotype		$F_{3113,275.5}$ = 3.399	0.0171
**5b (right)**	Kruskal-Wallis ANOVA			
	Genotype		H_3 = 8.783	0.0124
	Dunn's test	*R23E10 ∩ VGlut-GAL4>VGlut*$^{RNAi\ 104324KK}$ vs. *R23E10 ∩ VGlut-GAL4*	Z = 2.555	0.0213
	Dunn's test	*R23E10 ∩ VGlut-GAL4>VGlut*$^{RNAi\ 104324KK}$ vs. *VGlut*$^{RNAi\ 104324KK}$	Z = 2.503	0.0246

**5c**	One-way ANOVA<br>Genotype<br>Holm-Šidák test<br>Holm-Šidák test<br>Holm-Šidák test<br>Holm-Šidák test	 <br>*R23E10 ∩ R84C10>VGlutRNAi*^{104324KK} vs. *R23E10 ∩ R84C10*<br>*R23E10 ∩ R84C10>VGlutRNAi*^{104324KK} vs. *VGlutRNAi*^{104324KK}<br>*R23E10 ∩ R84C10>VGlut*$^{RNAi\ 2574GD}$ vs. *R23E10 ∩ R84C10*<br>*R23E10 ∩ R84C10>VGlut*$^{RNAi\ 2574GD}$ vs. *VGlut*$^{RNAi\ 2574GD}$	 $F_{4,461}$ = 4.871<br>t_{461}= 2.825<br>t_{461}= 3.317<br>t_{461}= 2.328<br>t_{461}= 2.805	 0.0007<br>0.0147<br>0.0039<br>0.0203<br>0.0147
**5d (left)**	Two-way repeated-measures ANOVA<br>Time<br>Genotype<br>Time x genotype		 $F_{1.651,196.4}$ = 2.597<br>$F_{2,119}$ = 5.814<br>$F_{94,5593}$ = 5.712	 0.0874<br>0.0039<br><0.0001
**5d (right)**	Kruskal-Wallis ANOVA<br>Genotype<br>Dunn's test<br>Dunn's test	 <br>*R23E10 ∩ R84C10>VGlutRNAi*^{104324KK} vs. *R23E10 ∩ R84C10*<br>*R23E10 ∩ R84C10>VGlutRNAi*^{104324KK} vs. *VGlutRNAi*^{104324KK}	 H_3 = 15.45<br>Z = 2.496<br>Z = 3.923	 0.0004<br>0.0251<br>0.0002
**5e (right)**	Two-sided Mann-Whitney test	*R23E10 ∩ R84C10>VGlut*$^{RNAi\ 2574GD}$ vs. *R23E10 ∩ R84C10*	U = 1	<0.0001
**6b**	Two-sided Mann-Whitney test	Rested vs. sleep-deprived	U = 522	<0.0001
**6c**	Two-sided Mann-Whitney test	Rested vs. sleep-deprived	U = 661	0.0005
**6d**	Kuskal-Wallis ANOVA<br>Sleep history<br>Dunn's test<br>Dunn's test<br>Dunn's test<br>Dunn's test	 <br>0 h vs. 3 h<br>0 h vs. 6 h<br>0 h vs. 9 h<br>0 h vs. 12 h	 H_5 = 14.62<br>Z = 0.4286<br>Z = 0.3088<br>Z = 1.562<br>Z = 3.297	 0.0056<br>>0.9999<br>>0.9999<br>0.4732<br>0.0039
**6e**	Kruskal-Wallis ANOVA<br>Zeitgeber time<br>Dunn's test<br>Dunn's test<br>Dunn's test<br>Dunn's test<br>Dunn's test<br><br>Kruskal-Wallis ANOVA<br>Sleep history<br>Dunn's test<br>Dunn's test<br>Dunn's test<br>Dunn's test<br>Dunn's test<br>Dunn's test	 <br>Rested: ZT 0–4 vs. ZT 4–8<br>Rested: ZT 0–4 vs. ZT 8–12<br>Rested: ZT 0–4 vs. ZT 12–16<br>Rested: ZT 0–4 vs. ZT 16–23<br>Rested: ZT 0–4 vs. ZT 23–4<br><br><br><br>ZT 0–4: Rested vs. sleep-deprived<br>ZT 4–8: Rested vs. sleep-deprived<br>ZT 8–12: Rested vs. sleep-deprived<br>ZT 12–16: Rested vs. sleep-deprived<br>ZT 16–23: Rested vs. sleep-deprived<br>ZT 23–4: Rested vs. sleep-deprived	 H_6 = 20.42<br>Z = 1.974<br>Z = 2.706<br>Z = 2.851<br>Z = 0.1517<br>Z = 1.712<br><br><br>H_{12} = 38.18<br>Z = 3.359<br>Z = 0.9144<br>Z = 1.534<br>Z = 0.5184<br>Z = 1.006<br>Z = 0.5589	 0.0010<br>0.2418<br>0.0340<br>0.0218<br>>0.9999<br>0.4344<br><br><br><0.0001<br>0.0047<br>>0.9999<br>0.7505<br>>0.9999<br>>0.9999<br>>0.9999
**7h**	Two-way repeated-measures ANOVA<br>Illumination<br>Genotype<br>Illumination x genotype<br>Holm-Šidák test<br>Holm-Šidák test<br>Holm-Šidák test	 <br><br><br>Pre (- *Chrimson* vs. + *Chrimson*)<br>Light (- *Chrimson* vs. + *Chrimson*)<br>Post (- *Chrimson* vs. + *Chrimson*)	 $F_{2,42}$ = 14.74<br>$F_{1,21}$ = 22.66<br>$F_{2,42}$ = 7.469<br>t_{63} = 1.404<br>t_{63} = 6.027<br>t_{63} = 2.153	 <0.0001<br>0.0001<br>0.0017<br>0.1652<br><0.0001<br>0.0691
**8a**	Two-sided unpaired *t*-test	Rested vs. sleep-deprived	t_{49} = 3.522	0.0009
**8b**	Two-way repeated-measures ANOVA<br>Light intensity<br>Sleep history<br>Light intensity x sleep history		 $F_{1.156,24.28}$ = 334.0<br>$F_{1,21}$ = 3.958<br>$F_{4,84}$ = 6.170	 <0.0001<br>0.0598<br>0.0002

Supplementary Table 3. Statistical analyses of Extended Data Figures 1–10.

Figure (ED)	Statistical test	Pairwise comparison	Test statistic	*P*
**1a**	Two-sided Mann-Whitney test	Control vs. implanted with chronic imaging window	U = 226.5	0.5419
**1b**	Two-way repeated-measures ANOVA			
	Time		$F_{14.21,1549} = 81.82$	<0.0001
	Window implantation		$F_{1,109} = 1.423$	0.2355
	Time x window implantation		$F_{47,5123} = 0.5874$	0.9890
**2d**	Two-sided unpaired *t*-test	Rested vs. sleep-deprived	$t_{39} = 3.468$	0.0013
**2e**	Two-sided unpaired *t*-test	Rested vs. sleep-deprived	$t_{39} = 2.629$	0.0122
**2f**	Kuskal-Wallis ANOVA			
	Sleep history		$H_5 = 15.11$	0.0045
	Dunn's test	0 h vs. 3 h	Z = 0.2235	>0.9999
	Dunn's test	0 h vs. 6 h	Z = 0.1542	>0.9999
	Dunn's test	0 h vs. 9 h	Z = 2.508	0.0485
	Dunn's test	0 h vs. 12 h	Z = 3.256	0.0045
**2g**	Kruskal-Wallis ANOVA			
	Zeitgeber time		$H_6 = 20.31$	0.0011
	Dunn's test	Rested: ZT 0–4 vs. ZT 4–8	Z = 1.602	0.5454
	Dunn's test	Rested: ZT 0–4 vs. ZT 8–12	Z = 2.687	0.0361
	Dunn's test	Rested: ZT 0–4 vs. ZT 12–16	Z = 2709	0.0337
	Dunn's test	Rested: ZT 0–4 vs. ZT 16–23	Z = 0.2439	>0.9999
	Dunn's test	Rested: ZT 0–4 vs. ZT 23–4	Z = 1.805	0.3553
	Kruskal-Wallis ANOVA			
	Sleep history		$H_{12} = 41.73$	<0.0001
	Dunn's test	ZT 0–4: Rested vs. sleep-deprived	Z = 3.471	0.0031
	Dunn's test	ZT 4–8: Rested vs. sleep-deprived	Z = 1.105	>0.9999
	Dunn's test	ZT 8–12: Rested vs. sleep-deprived	Z = 1.439	0.9007
	Dunn's test	ZT 12–16: Rested vs. sleep-deprived	Z = 0.5795	>0.9999
	Dunn's test	ZT 16–23: Rested vs. sleep-deprived	Z = 1.346	>0.9999
	Dunn's test	ZT 23–4: Rested vs. sleep-deprived	Z = 0.2803	>0.9999
**3c**	Two-sided Mann-Whitney test	Same layer vs. different layer	U = 18821	<0.0001
**3d, e**	Two-sided Mann-Whitney test	Same cell type vs. different cell type	U = 5285	<0.0001
**3h**	Two-sided Mann-Whitney test	Same layer vs. different layer	U = 24731	<0.0001
**3i, j**	Two-sided Mann-Whitney test	Same cell type vs. different cell type	U = 46519	<0.0001
**6a (left)**	One-way ANOVA			
	Genotype		$F_{8,442} = 20.16$	<0.0001
	Holm-Šidák test	$R23E10>VGlut^{RNAi\ 104324KK}$ vs. *R23E10*	$t_{442} = 7.488$	<0.0001
	Holm-Šidák test	$R23E10>VGlut^{RNAi\ 104324KK}$ vs. $VGlut^{RNAi\ 104324KK}$	$t_{442} = 2.881$	0.0077
	Holm-Šidák test	$R23E10>VGlut^{RNAi\ TRiP\ \#27538}$ vs. *R23E10*	$t_{442} = 7.497$	<0.0001
	Holm-Šidák test	$R23E10>VGlut^{RNAi\ TRiP\ \#27538}$ vs. $VGlut^{RNAi\ TRiP\ \#27538}$	$t_{442} = 6.782$	<0.0001
	Holm-Šidák test	$R23E10>VGlut^{RNAi\ 2574GD}$ vs. *R23E10*	$t_{442} = 8.919$	<0.0001
	Holm-Šidák test	$R23E10>VGlut^{RNAi\ 2574GD}$ vs. $VGlut^{RNAi\ 2574GD}$	$t_{442} = 5.084$	<0.0001
	Holm-Šidák test	$R23E10>VGlut^{RNAi\ TRiP\ \#40927}$ vs. *R23E10*	$t_{442} = 4.090$	0.0002
	Holm-Šidák test	$R23E10>VGlut^{RNAi\ TRiP\ \#40927}$ vs. $VGlut^{RNAi\ TRiP\ \#40927}$	$t_{442} = 2.903$	0.0077
**6a (right)**	Kruskal-Wallis ANOVA			
	Genotype		$H_9 = 80.60$	<0.0001
	Dunn's test	$R23E10>VGlut^{RNAi\ 104324KK}$ vs. *R23E10*	Z = 1.940	0.4195
	Dunn's test	$R23E10>VGlut^{RNAi\ 104324KK}$ vs. $VGlut^{RNAi\ 104324KK}$	Z = 2.901	0.0298
	Dunn's test	$R23E10>VGlut^{RNAi\ TRiP\ \#27538}$ vs. *R23E10*	Z = 0.8568	>0.9999
	Dunn's test	$R23E10>VGlut^{RNAi\ TRiP\ \#27538}$ vs. $VGlut^{RNAi\ TRiP\ \#27538}$	Z = 0.6311	>0.9999
	Dunn's test	$R23E10>VGlut^{RNAi\ 2574GD}$ vs. *R23E10*	Z = 2.326	0.1601
	Dunn's test	$R23E10>VGlut^{RNAi\ 2574GD}$ vs. $VGlut^{RNAi\ 2574GD}$	Z = 4.648	<0.0001
	Dunn's test	$R23E10>VGlut^{RNAi\ TRiP\ \#40927}$ vs. *R23E10*	Z = 3.045	0.0186
	Dunn's test	$R23E10>VGlut^{RNAi\ TRiP\ \#40927}$ vs. $VGlut^{RNAi\ TRiP\ \#40927}$	Z = 2.089	0.2937
**6b (left)**	Two-way repeated-measures ANOVA			
	Time		$F_{1.476,175.7} = 29.12$	<0.0001
	Genotype		$F_{2,119} = 4.178$	0.0176
	Time x genotype		$F_{94,5593} = 5.473$	<0.0001
**6b (center)**	Two-way repeated-measures ANOVA			
	Time		$F_{1.596,178.7} = 3.425$	0.04511
	Genotype		$F_{2,112} = 10.64$	<0.0001
	Time x genotype		$F_{94,5264} = 15.73$	<0.0001

**6b (right)**	Kruskal-Wallis ANOVA			
	Genotype		H_5 = 55.91	<0.0001
	Dunn's test	*R23E10>VGlut*$^{RNAi\ 104324KK}$ vs. *R23E10*	Z = 3.193	0.0056
	Dunn's test	*R23E10>VGlut*$^{RNAi\ 104324KK}$ vs. *VGlut*$^{RNAi\ 104324KK}$	Z = 3.841	0.0005
	Dunn's test	*R23E10>VGlut*$^{RNAi\ TRiP\ \#27538}$ vs. *R23E10*	Z = 5.874	<0.0001
	Dunn's test	*R23E10>VGlut*$^{RNAi\ TRiP\ \#27538}$ vs. *VGlut*$^{RNAi\ TRiP\ \#27538}$	Z = 2.996	0.0109
**6c (left)**	Kruskal-Wallis ANOVA			
	Genotype		H_9 = 143.9	<0.0001
	Dunn's test	*R23E10 ∩ VGlut-GAL4>VGlut*$^{RNAi\ 104324KK}$ vs. *R23E10 ∩ VGlut-GAL4*	Z = 4.712	<0.0001
	Dunn's test	*R23E10 ∩ VGlut-GAL4>VGlut*$^{RNAi\ 104324KK}$ vs. *VGlut*$^{RNAi\ 104324KK}$	Z = 5.898	<0.0001
	Dunn's test	*R23E10 ∩ VGlut-GAL4>VGlut*$^{RNAi\ 2574GD}$ vs. *R23E10 ∩ VGlut-GAL4*	Z = 6.468	<0.0001
	Dunn's test	*R23E10 ∩ VGlut-GAL4>VGlut*$^{RNAi\ 2574GD}$ vs. *VGlut*$^{RNAi\ 2574GD}$	Z = 8.060	<0.0001
	Dunn's test	*R23E10 ∩ VGlut-GAL4>EKO* vs. *R23E10 ∩ VGlut-GAL4*	Z = 4.500	<0.0001
	Dunn's test	*R23E10 ∩ VGlut-GAL4>EKO* vs. *EKO*	Z = 5.152	<0.0001
	Dunn's test	*R23E10 ∩ VGlut-GAL4>Kir2.1* vs. *R23E10 ∩ VGlut-GAL4*	Z = 3.211	0.0106
	Dunn's test	*R23E10 ∩ VGlut-GAL4> Kir2.1* vs. *Kir2.1*	Z = 3.140	0.0135
**6c (right)**	Kruskal-Wallis ANOVA			
	Genotype		H_9 = 64.72	<0.0001
	Dunn's test	*R23E10 ∩ VGlut-GAL4>VGlut*$^{RNAi\ 104324KK}$ vs. *R23E10 ∩ VGlut-GAL4*	Z = 2.633	0.0678
	Dunn's test	*R23E10 ∩ VGlut-GAL4>VGlut*$^{RNAi\ 104324KK}$ vs. *VGlut*$^{RNAi\ 104324KK}$	Z = 1.196	>0.9999
	Dunn's test	*R23E10 ∩ VGlut-GAL4>VGlut*$^{RNAi\ 2574GD}$ vs. *R23E10 ∩ VGlut-GAL4*	Z = 0.7564	>0.9999
	Dunn's test	*R23E10 ∩ VGlut-GAL4>VGlut*$^{RNAi\ 2574GD}$ vs. *VGlut*$^{RNAi\ 2574GD}$	Z = 3.058	0.0178
	Dunn's test	*R23E10 ∩ VGlut-GAL4>EKO* vs. *R23E10 ∩ VGlut-GAL4*	Z = 4.345	0.0001
	Dunn's test	*R23E10 ∩ VGlut-GAL4>EKO* vs. *EKO*	Z = 0.8793	>0.9999
	Dunn's test	*R23E10 ∩ VGlut-GAL4>Kir2.1* vs. *R23E10 ∩ VGlut-GAL4*	Z = 0.2990	>0.9999
	Dunn's test	*R23E10 ∩ VGlut-GAL4> Kir2.1* vs. *Kir2.1*	Z = 0.9073	>0.9999
**6d (left)**	Two-way repeated-measures ANOVA			
	Time		$F_{1.556,275.5}$ = 36.70	<0.0001
	Genotype		$F_{2,177}$ = 3.693	0.0268
	Time x genotype		$F_{3113,275.5}$ = 3.399	0.0171
**6d (center)**	Two-way repeated-measures ANOVA			
	Time		$F_{1.535,244.1}$ = 35.09	<0.0001
	Genotype		$F_{2,159}$ = 3.538	0.0314
	Time x genotype		$F_{94,7473}$ = 3.220	<0.0001
**6d (right)**	Kruskal-Wallis ANOVA			
	Genotype		H_5 = 20.32	0.0004
	Dunn's test	*R23E10 ∩ VGlut-GAL4>VGlut*$^{RNAi\ 104324KK}$ vs. *R23E10 ∩ VGlut-GAL4*	Z = 2.552	0.0429
	Dunn's test	*R23E10 ∩ VGlut-GAL4>VGlut*$^{RNAi\ 104324KK}$ vs. *VGlut*$^{RNAi\ 104324KK}$	Z = 2.549	0.0432
	Dunn's test	*R23E10 ∩ VGlut-GAL4>VGlut*$^{RNAi\ 2574GD}$ vs. *R23E10 ∩ VGlut-GAL4*	Z = 2.633	0.0339
	Dunn's test	*R23E10 ∩ VGlut-GAL4>VGlut*$^{RNAi\ 2574GD}$ vs. *VGlut*$^{RNAi\ 2574GD}$	Z = 3.374	0.0030
**6e,**	Two-way repeated-measures ANOVA			
104324KK	Time		$F_{1.543,415.1}$ = 5.475	0.0088
	Genotype		$F_{2,269}$ = 4.829	0.0087
	Time x genotype		$F_{94,12643}$ = 9.672	<0.0001
**6e,**	Two-way repeated-measures ANOVA			
2574GD	Time		$F_{1.626,325.3}$ = 2.607	0.0865
	Genotype		$F_{2,200}$ = 18.03	<0.0001
	Time x genotype		$F_{94,9400}$ = 17.85	<0.0001
**6e,**	Two-way repeated-measures ANOVA			
EKO	Time		$F_{1.558,339.5}$ = 17.43	<0.0001
	Genotype		$F_{2,218}$ = 20.56	<0.0001
	Time x genotype		$F_{94,10246}$ = 40.35	<0.0001
**6e (right)**	Kruskal-Wallis ANOVA			
	Genotype		H_7 = 115.6	<0.0001
	Dunn's test	*R23E10 ∩ VGlut-GAL4>VGlut*$^{RNAi\ 104324KK}$ vs. *R23E10 ∩ VGlut-GAL4*	Z = 2.831	0.0278
	Dunn's test	*R23E10 ∩ VGlut-GAL4>VGlut*$^{RNAi\ 104324KK}$ vs. *VGlut*$^{RNAi\ 104324KK}$	Z = 3.815	0.0008
	Dunn's test	*R23E10 ∩ VGlut-GAL4>VGlut*$^{RNAi\ 2574GD}$ vs. *R23E10 ∩ VGlut-GAL4*	Z = 3.152	0.0097
	Dunn's test	*R23E10 ∩ VGlut-GAL4>VGlut*$^{RNAi\ 2574GD}$ vs. *VGlut*$^{RNAi\ 2574GD}$	Z = 5.820	<0.0001
	Dunn's test	*R23E10 ∩ VGlut-GAL4>EKO* vs. *R23E10 ∩ VGlut-GAL4*	Z = 6.450	<0.0001
	Dunn's test	*R23E10 ∩ VGlut-GAL4>EKO* vs. *EKO*	Z = 6.914	<0.0001
**6f**	Kruskal-Wallis ANOVA			
	Genotype		H_5 = 28.07	<0.0001
	Dunn's test	*R23E10 ∩ R84C10>VGlutRNAi*^{104324KK} vs. *R23E10 ∩ R84C10*	Z = 0.0611	>0.9999
	Dunn's test	*R23E10 ∩ R84C10>VGlutRNAi*^{104324KK} vs. *VGlutRNAi*^{104324KK}	Z = 2.704	0.0274
	Dunn's test	*R23E10 ∩ R84C10>VGlut*$^{RNAi\ 2574GD}$ vs. *R23E10 ∩ R84C10*	Z = 4.400	<0.0001
	Dunn's test	*R23E10 ∩ R84C10>VGlut*$^{RNAi\ 2574GD}$ vs. *VGlut*$^{RNAi\ 2574GD}$	Z = 2.085	0.1483
**6g**	Two-way repeated-measures ANOVA			
	Time		$F_{30.69,4910}$ = 60.30	<0.0001
	Genotype		$F_{2,160}$ = 10.64	<0.0001
	Time x genotype		$F_{61.37,4910}$ = 2.762	<0.0001

**6h (left)**	Kruskal-Wallis ANOVA			
	Genotype		$H_3 = 14.16$	0.0008
	Dunn's test	*R23E10 ∩ R84C10>VGlut*$^{RNAi\ 2574GD}$ vs. *R23E10 ∩ R84C10*	$Z = 2.925$	0.0069
	Dunn's test	*R23E10 ∩ R84C10>VGlut*$^{RNAi\ 2574GD}$ vs. *VGlut*$^{RNAi\ 2574GD}$	$Z = 3.525$	0.0008
**6h (right)**	Kruskal-Wallis ANOVA			
	Genotype		$H_3 = 10.65$	0.0049
	Dunn's test	*R23E10 ∩ R84C10>VGlut*$^{RNAi\ 2574GD}$ vs. *R23E10 ∩ R84C10*	$Z = 3.161$	0.0031
	Dunn's test	*R23E10 ∩ R84C10>VGlut*$^{RNAi\ 2574GD}$ vs. *VGlut*$^{RNAi\ 2574GD}$	$Z = 2.302$	0.0426
**7a (left)**	Two-way repeated-measures ANOVA			
	Time		$F_{25.39,7439} = 184.2$	<0.0001
	Genotype		$F_{2,293} = 27.67$	<0.0001
	Time x genotype		$F_{190,27835} = 4.888$	<0.0001
**7a (center)**	Two-way repeated-measures ANOVA			
	Time		$F_{11.97,3221} = 149.5$	<0.0001
	Genotype		$F_{2,269} = 8.149$	0.0004
	Time x Genotype		$F_{94,12643} = 3.843$	<0.0001
**7a (right)**	Two-way repeated-measures ANOVA			
	Time		$F_{13.09,2317} = 135.6$	<0.0001
	Genotype		$F_{2,177} = 10.67$	<0.0001
	Time x genotype		$F_{94,8319} = 3.650$	<0.0001
**7b (left)**	Two-way repeated-measures ANOVA			
	Time		$F_{25.59,6219} = 123.0$	<0.0001
	Genotype		$F_{2,243} = 43.99$	<0.0001
	Time x genotype		$F_{190,23085} = 2.659$	<0.0001
**7b (center)**	Two-way repeated-measures ANOVA			
	Time		$F_{13.33,2667} = 85.40$	<0.0001
	Genotype		$F_{2,200} = 55.52$	<0.0001
	Time x genotype		$F_{94,9400} = 7.430$	<0.0001
**7b (right)**	Two-way repeated-measures ANOVA			
	Time		$F_{14.29,2272} = 111.3$	<0.0001
	Genotype		$F_{2,159} = 26.06$	<0.0001
	Time x genotype		$F_{94,7473} = 2.935$	<0.0001
**7c (left)**	Two-way repeated-measures ANOVA			
	Time		$F_{30.74,5133} = 89.51$	<0.0001
	Genotype		$F_{2,167} = 21.07$	<0.0001
	Time x genotype		$F_{190,15865} = 5.248$	<0.0001
**7c (right)**	Two-way repeated-measures ANOVA			
	Time		$F_{13.92,3034} = 48.87$	<0.0001
	Genotype		$F_{2,218} = 63.26$	<0.0001
	Time x genotype		$F_{94,10246} = 14.94$	<0.0001
**7d (right)**	Two-way repeated-measures ANOVA			
	Time		$F_{20.62,4062} = 163.3$	<0.0001
	Genotype		$F_{2,197} = 4.811$	0.0091
	Time x genotype		$F_{41.24,4062} = 3.092$	<0.0001
**7e (left)**	Two-way repeated-measures ANOVA			
	Time		$F_{25.87,7167} = 229.3$	<0.0001
	Genotype		$F_{2,277} = 6.314$	0.0021
	Time x genotype		$F_{51.75,7167} = 3.762$	<0.0001
**7e (right)**	Two-way repeated-measures ANOVA			
	Time		$F_{8.761,1043} = 40.18$	<0.0001
	Genotype		$F_{2,119} = 9.540$	0.0001
	Time x genotype		$F_{94,5593} = 2.385$	<0.0001
**7f**	Three-way ANOVA			
	Sleep history		$F_1 = 18.24$	<0.0001
	Sleep deprivation method		$F_1 = 1.45$	0.2287
	Genotype		$F_2 = 0.53$	0.5877
**8a, Bout length**	Kruskal-Wallis ANOVA			
	Genotype		$H_9 = 154.1$	<0.0001
	Dunn's test	*R23E10>VGlut*$^{RNAi\ 104324KK}$ vs. *R23E10*	$Z = 4.922$	<0.0001
	Dunn's test	*R23E10>VGlut*$^{RNAi\ 104324KK}$ vs. *VGlut*$^{RNAi\ 104324KK}$	$Z = 2.982$	0.0229
	Dunn's test	*R23E10>VGlut*$^{RNAi\ TRiP\ \#27538}$ vs. *R23E10*	$Z = 7.408$	<0.0001
	Dunn's test	*R23E10>VGlut*$^{RNAi\ TRiP\ \#27538}$ vs. *VGlut*$^{RNAi\ TRiP\ \#27538}$	$Z = 8.468$	<0.0001
	Dunn's test	*R23E10>VGlut*$^{RNAi\ 2574GD}$ vs. *R23E10*	$Z = 6.876$	<0.0001
	Dunn's test	*R23E10>VGlut*$^{RNAi\ 2574GD}$ vs. *VGlut*$^{RNAi\ 2574GD}$	$Z = 4.747$	<0.0001
	Dunn's test	*R23E10>VGlut*$^{RNAi\ TRiP\ \#40927}$ vs. *R23E10*	$Z = 6.217$	<0.0001
	Dunn's test	*R23E10>VGlut*$^{RNAi\ TRiP\ \#40927}$ vs. *VGlut*$^{RNAi\ TRiP\ \#40927}$	$Z = 4.200$	0.0002

**8a, Bout number**	Kruskal-Wallis ANOVA			
	Genotype		$H_9 = 96.10$	<0.0001
	Dunn's test	*R23E10>VGlut*$^{RNAi\ 104324KK}$ vs. *R23E10*	Z = 1.325	>0.9999
	Dunn's test	*R23E10>VGlut*$^{RNAi\ 104324KK}$ vs. *VGlut*$^{RNAi\ 104324KK}$	Z = 2.089	0.2939
	Dunn's test	*R23E10>VGlut*$^{RNAi\ TRiP\ \#27538}$ vs. *R23E10*	Z = 4.906	<0.0001
	Dunn's test	*R23E10>VGlut*$^{RNAi\ TRiP\ \#27538}$ vs. *VGlut*$^{RNAi\ TRiP\ \#27538}$	Z = 7.082	<0.0001
	Dunn's test	*R23E10>VGlut*$^{RNAi\ 2574GD}$ vs. *R23E10*	Z = 1.351	>0.9999
	Dunn's test	*R23E10>VGlut*$^{RNAi\ 2574GD}$ vs. *VGlut*$^{RNAi\ 2574GD}$	Z = 0.556	>0.9999
	Dunn's test	*R23E10>VGlut*$^{RNAi\ TRiP\ \#40927}$ vs. *R23E10*	Z = 6.012	<0.0001
	Dunn's test	*R23E10>VGlut*$^{RNAi\ TRiP\ \#40927}$ vs. *VGlut*$^{RNAi\ TRiP\ \#40927}$	Z = 3.946	0.0006
**8a, P(Wake)**	Kruskal-Wallis ANOVA			
	Genotype		$H_9 = 134.9$	<0.0001
	Dunn's test	*R23E10>VGlut*$^{RNAi\ 104324KK}$ vs. *R23E10*	Z = 6.200	<0.0001
	Dunn's test	*R23E10>VGlut*$^{RNAi\ 104324KK}$ vs. *VGlut*$^{RNAi\ 104324KK}$	Z = 2.256	0.1926
	Dunn's test	*R23E10>VGlut*$^{RNAi\ TRiP\ \#27538}$ vs. *R23E10*	Z = 7.316	<0.0001
	Dunn's test	*R23E10>VGlut*$^{RNAi\ TRiP\ \#27538}$ vs. *VGlut*$^{RNAi\ TRiP\ \#27538}$	Z = 6.756	<0.0001
	Dunn's test	*R23E10>VGlut*$^{RNAi\ 2574GD}$ vs. *R23E10*	Z = 7.971	<0.0001
	Dunn's test	*R23E10>VGlut*$^{RNAi\ 2574GD}$ vs. *VGlut*$^{RNAi\ 2574GD}$	Z = 4.527	<0.0001
	Dunn's test	*R23E10>VGlut*$^{RNAi\ TRiP\ \#40927}$ vs. *R23E10*	Z = 5.477	<0.0001
	Dunn's test	*R23E10>VGlut*$^{RNAi\ TRiP\ \#40927}$ vs. *VGlut*$^{RNAi\ TRiP\ \#40927}$	Z = 3.187	0.0115
**8a, P(Doze)**	Kruskal-Wallis ANOVA			
	Genotype		$H_9 = 68.67$	<0.0001
	Dunn's test	*R23E10>VGlut*$^{RNAi\ 104324KK}$ vs. *R23E10*	Z = 2.317	0.1639
	Dunn's test	*R23E10>VGlut*$^{RNAi\ 104324KK}$ vs. *VGlut*$^{RNAi\ 104324KK}$	Z = 0.685	>0.9999
	Dunn's test	*R23E10>VGlut*$^{RNAi\ TRiP\ \#27538}$ vs. *R23E10*	Z = 1.449	>0.9999
	Dunn's test	*R23E10>VGlut*$^{RNAi\ TRiP\ \#27538}$ vs. *VGlut*$^{RNAi\ TRiP\ \#27538}$	Z = 2.204	0.2200
	Dunn's test	*R23E10>VGlut*$^{RNAi\ 2574GD}$ vs. *R23E10*	Z = 2.145	0.2553
	Dunn's test	*R23E10>VGlut*$^{RNAi\ 2574GD}$ vs. *VGlut*$^{RNAi\ 2574GD}$	Z = 1.474	>0.9999
	Dunn's test	*R23E10>VGlut*$^{RNAi\ TRiP\ \#40927}$ vs. *R23E10*	Z = 4.944	<0.0001
	Dunn's test	*R23E10>VGlut*$^{RNAi\ TRiP\ \#40927}$ vs. *VGlut*$^{RNAi\ TRiP\ \#40927}$	Z = 1.576	0.9195
**8b, Bout length**	Kruskal-Wallis ANOVA			
	Genotype		$H_9 = 318.4$	<0.0001
	Dunn's test	*R23E10 ∩ VGlut-GAL4>VGlut*$^{RNAi\ 104324KK}$ vs. *R23E10 ∩ VGlut-GAL4*	Z = 4.796	<0.0001
	Dunn's test	*R23E10 ∩ VGlut-GAL4>VGlut*$^{RNAi\ 104324KK}$ vs. *VGlut*$^{RNAi\ 104324KK}$	Z = 9.338	<0.0001
	Dunn's test	*R23E10 ∩ VGlut-GAL4>VGlut*$^{RNAi\ 2574GD}$ vs. *R23E10 ∩ VGlut-GAL4*	Z = 6.929	<0.0001
	Dunn's test	*R23E10 ∩ VGlut-GAL4>VGlut*$^{RNAi\ 2574GD}$ vs. *VGlut*$^{RNAi\ 2574GD}$	Z = 10.98	<0.0001
	Dunn's test	*R23E10 ∩ VGlut-GAL4>EKO* vs. *R23E10 ∩ VGlut-GAL4*	Z = 6.752	<0.0001
	Dunn's test	*R23E10 ∩ VGlut-GAL4>EKO* vs. *EKO*	Z = 4.045	0.0004
	Dunn's test	*R23E10 ∩ VGlut-GAL4>Kir2.1* vs. *R23E10 ∩ VGlut-GAL4*	Z = 2.202	0.2214
	Dunn's test	*R23E10 ∩ VGlut-GAL4> Kir2.1* vs. *Kir2.1*	Z = 4.270	0.0002
**8b, Bout number**	Kruskal-Wallis ANOVA			
	Genotype		$H_9 = 259.0$	<0.0001
	Dunn's test	*R23E10 ∩ VGlut-GAL4>VGlut*$^{RNAi\ 104324KK}$ vs. *R23E10 ∩ VGlut-GAL4*	Z = 2.615	0.0713
	Dunn's test	*R23E10 ∩ VGlut-GAL4>VGlut*$^{RNAi\ 104324KK}$ vs. *VGlut*$^{RNAi\ 104324KK}$	Z = 6.744	<0.0001
	Dunn's test	*R23E10 ∩ VGlut-GAL4>VGlut*$^{RNAi\ 2574GD}$ vs. *R23E10 ∩ VGlut-GAL4*	Z = 4.580	<0.0001
	Dunn's test	*R23E10 ∩ VGlut-GAL4>VGlut*$^{RNAi\ 2574GD}$ vs. *VGlut*$^{RNAi\ 2574GD}$	Z = 8.451	<0.0001
	Dunn's test	*R23E10 ∩ VGlut-GAL4>EKO* vs. *R23E10 ∩ VGlut-GAL4*	Z = 5.992	<0.0001
	Dunn's test	*R23E10 ∩ VGlut-GAL4>EKO* vs. *EKO*	Z = 2.115	0.2752
	Dunn's test	*R23E10 ∩ VGlut-GAL4>Kir2.1* vs. *R23E10 ∩ VGlut-GAL4*	Z = 1.338	>0.9999
	Dunn's test	*R23E10 ∩ VGlut-GAL4> Kir2.1* vs. *Kir2.1*	Z = 3.866	0.0009
**8b, P(Wake)**	Kruskal-Wallis ANOVA			
	Genotype		$H_9 = 197.4$	<0.0001
	Dunn's test	*R23E10 ∩ VGlut-GAL4>VGlut*$^{RNAi\ 104324KK}$ vs. *R23E10 ∩ VGlut-GAL4*	Z = 5.499	<0.0001
	Dunn's test	*R23E10 ∩ VGlut-GAL4>VGlut*$^{RNAi\ 104324KK}$ vs. *VGlut*$^{RNAi\ 104324KK}$	Z = 8.148	<0.0001
	Dunn's test	*R23E10 ∩ VGlut-GAL4>VGlut*$^{RNAi\ 2574GD}$ vs. *R23E10 ∩ VGlut-GAL4*	Z = 6.810	<0.0001
	Dunn's test	*R23E10 ∩ VGlut-GAL4>VGlut*$^{RNAi\ 2574GD}$ vs. *VGlut*$^{RNAi\ 2574GD}$	Z = 8.975	<0.0001
	Dunn's test	*R23E10 ∩ VGlut-GAL4>EKO* vs. *R23E10 ∩ VGlut-GAL4*	Z = 5.672	<0.0001
	Dunn's test	*R23E10 ∩ VGlut-GAL4>EKO* vs. *EKO*	Z = 4.914	<0.0001
	Dunn's test	*R23E10 ∩ VGlut-GAL4>Kir2.1* vs. *R23E10 ∩ VGlut-GAL4*	Z = 3.172	0.0121
	Dunn's test	*R23E10 ∩ VGlut-GAL4> Kir2.1* vs. *Kir2.1*	Z = 3.449	0.0045
**8b, P(Doze)**	Kruskal-Wallis ANOVA			
	Genotype		$H_9 = 193.8$	<0.0001
	Dunn's test	*R23E10 ∩ VGlut-GAL4>VGlut*$^{RNAi\ 104324KK}$ vs. *R23E10 ∩ VGlut-GAL4*	Z = 1.741	0.6530
	Dunn's test	*R23E10 ∩ VGlut-GAL4>VGlut*$^{RNAi\ 104324KK}$ vs. *VGlut*$^{RNAi\ 104324KK}$	Z = 4.259	0.0002
	Dunn's test	*R23E10 ∩ VGlut-GAL4>VGlut*$^{RNAi\ 2574GD}$ vs. *R23E10 ∩ VGlut-GAL4*	Z = 1.337	>0.9999
	Dunn's test	*R23E10 ∩ VGlut-GAL4>VGlut*$^{RNAi\ 2574GD}$ vs. *VGlut*$^{RNAi\ 2574GD}$	Z = 1.129	>0.9999
	Dunn's test	*R23E10 ∩ VGlut-GAL4>EKO* vs. *R23E10 ∩ VGlut-GAL4*	Z = 4.884	<0.0001
	Dunn's test	*R23E10 ∩ VGlut-GAL4>EKO* vs. *EKO*	Z = 1.111	>0.9999
	Dunn's test	*R23E10 ∩ VGlut-GAL4>Kir2.1* vs. *R23E10 ∩ VGlut-GAL4*	Z = 0.490	>0.9999
	Dunn's test	*R23E10 ∩ VGlut-GAL4> Kir2.1* vs. *Kir2.1*	Z = 0.403	>0.9999
**8c, Bout length**	Kruskal-Wallis ANOVA			
	Genotype		$H_5 = 54.92$	<0.0001
	Dunn's test	*R23E10 ∩ R84C10>VGlutRNAi*^{104324KK} vs. *R23E10 ∩ R84C10*	Z = 2.514	0.0478
	Dunn's test	*R23E10 ∩ R84C10>VGlutRNAi*^{104324KK} vs. *VGlutRNAi*^{104324KK}	Z = 4.149	0.0001
	Dunn's test	*R23E10 ∩ R84C10>VGlut*$^{RNAi\ 2574GD}$ vs. *R23E10 ∩ R84C10*	Z = 4.313	<0.0001
	Dunn's test	*R23E10 ∩ R84C10>VGlut*$^{RNAi\ 2574GD}$ vs. *VGlut*$^{RNAi\ 2574GD}$	Z = 5.958	<0.0001
**8c, Bout number**	Kruskal-Wallis ANOVA			
	Genotype		$H_5 = 46.16$	<0.0001

	Dunn's test<br>Dunn's test<br>Dunn's test<br>Dunn's test	R23E10 ∩ R84C10>VGlutRNAi^{104324KK} vs. R23E10 ∩ R84C10<br>R23E10 ∩ R84C10>VGlutRNAi^{104324KK} vs. VGlutRNAi^{104324KK}<br>R23E10 ∩ R84C10>VGlut$^{RNAi\ 2574GD}$ vs. R23E10 ∩ R84C10<br>R23E10 ∩ R84C10>VGlut$^{RNAi\ 2574GD}$ vs. VGlut$^{RNAi\ 2574GD}$	Z = 1.572<br>Z = 2.986<br>Z = 4.307<br>Z = 5.786	0.4642<br>0.0113<br><0.0001<br><0.0001
**8c, P(Wake)**	Kruskal-Wallis ANOVA<br>Genotype<br>Dunn's test<br>Dunn's test<br>Dunn's test<br>Dunn's test	 <br>R23E10 ∩ R84C10>VGlutRNAi^{104324KK} vs. R23E10 ∩ R84C10<br>R23E10 ∩ R84C10>VGlutRNAi^{104324KK} vs. VGlutRNAi^{104324KK}<br>R23E10 ∩ R84C10>VGlut$^{RNAi\ 2574GD}$ vs. R23E10 ∩ R84C10<br>R23E10 ∩ R84C10>VGlut$^{RNAi\ 2574GD}$ vs. VGlut$^{RNAi\ 2574GD}$	 H_5 = 28.93<br>Z = 2.165<br>Z = 3.733<br>Z = 2.360<br>Z = 3.814	 <0.0001<br>0.1215<br>0.0008<br>0.0732<br>0.0005
**8c, P(Doze)**	One-way ANOVA<br>Genotype<br>Holm-Šidák test<br>Holm-Šidák test<br>Holm-Šidák test<br>Holm-Šidák test	 <br>R23E10 ∩ R84C10>VGlutRNAi^{104324KK} vs. R23E10 ∩ R84C10<br>R23E10 ∩ R84C10>VGlutRNAi^{104324KK} vs. VGlutRNAi^{104324KK}<br>R23E10 ∩ R84C10>VGlut$^{RNAi\ 2574GD}$ vs. R23E10 ∩ R84C10<br>R23E10 ∩ R84C10>VGlut$^{RNAi\ 2574GD}$ vs. VGlut$^{RNAi\ 2574GD}$	 $F_{4,461}$ = 5.104<br>t_{461} = 0.8515<br>t_{461} = 2.426<br>t_{461} = 0.654<br>t_{461} = 1.529	 0.0005<br>0.6339<br>0.0611<br>0.6339<br>0.3345
**8d, Bout length**	Kruskal-Wallis ANOVA<br>Genotype<br>Dunn's test<br>Dunn's test<br>Dunn's test<br>Dunn's test	 <br>tsh-GAL80;R23E10>VGlutRNAi^{104324KK} vs. tsh-GAL80;R23E10<br>tsh-GAL80;R23E10>VGlutRNAi^{104324KK} vs. VGlutRNAi^{104324KK}<br>tsh-GAL80;R23E10>VGlut$^{RNAi\ 2574GD}$ vs. tsh-GAL80;R23E10<br>tsh-GAL80;R23E10>VGlut$^{RNAi\ 2574GD}$ vs. VGlut$^{RNAi\ 2574GD}$	 H_5 = 139.1<br>Z = 4.520<br>Z = 6.629<br>Z = 5.579<br>Z = 8.945	 <0.0001<br><0.0001<br><0.0001<br><0.0001<br><0.0001
**8d, Bout number**	Kruskal-Wallis ANOVA<br>Genotype<br>Dunn's test<br>Dunn's test<br>Dunn's test<br>Dunn's test	 <br>tsh-GAL80;R23E10>VGlutRNAi^{104324KK} vs. tsh-GAL80;R23E10<br>tsh-GAL80;R23E10>VGlutRNAi^{104324KK} vs. VGlutRNAi^{104324KK}<br>tsh-GAL80;R23E10>VGlut$^{RNAi\ 2574GD}$ vs. tsh-GAL80;R23E10<br>tsh-GAL80;R23E10>VGlut$^{RNAi\ 2574GD}$ vs. VGlut$^{RNAi\ 2574GD}$	 H_5 = 58.27<br>Z = 3.491<br>Z = 6.787<br>Z = 0.078<br>Z = 3.356	 <0.0001<br>0.0019<br><0.0001<br>>0.9999<br>0.0032
**8d, P(Wake)**	Kruskal-Wallis ANOVA<br>Genotype<br>Dunn's test<br>Dunn's test<br>Dunn's test<br>Dunn's test	 <br>tsh-GAL80;R23E10>VGlutRNAi^{104324KK} vs. tsh-GAL80;R23E10<br>tsh-GAL80;R23E10>VGlutRNAi^{104324KK} vs. VGlutRNAi^{104324KK}<br>tsh-GAL80;R23E10>VGlut$^{RNAi\ 2574GD}$ vs. tsh-GAL80;R23E10<br>tsh-GAL80;R23E10>VGlut$^{RNAi\ 2574GD}$ vs. VGlut$^{RNAi\ 2574GD}$	 H_5 = 80.00<br>Z = 5.576<br>Z = 4.693<br>Z = 6.525<br>Z = 5.909	 <0.0001<br><0.0001<br><0.0001<br><0.0001<br><0.0001
**8d, P(Doze)**	Kruskal-Wallis ANOVA<br>Genotype<br>Dunn's test<br>Dunn's test<br>Dunn's test<br>Dunn's test	 <br>tsh-GAL80;R23E10>VGlutRNAi^{104324KK} vs. tsh-GAL80;R23E10<br>tsh-GAL80;R23E10>VGlutRNAi^{104324KK} vs. VGlutRNAi^{104324KK}<br>tsh-GAL80;R23E10>VGlut$^{RNAi\ 2574GD}$ vs. tsh-GAL80;R23E10<br>tsh-GAL80;R23E10>VGlut$^{RNAi\ 2574GD}$ vs. VGlut$^{RNAi\ 2574GD}$	 H_5 = 39.38<br>Z = 1.445<br>Z = 3.713<br>Z = 3.409<br>Z = 1.467	 <0.0001<br>0.5939<br>0.0008<br>0.0026<br>0.5693
**8e, Bout length**	Two-way ANOVA<br>Temperature<br>Genotype<br>Temperature x genotype<br>Holm-Šidák test<br>Holm-Šidák test<br>Holm-Šidák test<br>Holm-Šidák test	 <br><br><br>18 °C: tub-GAL80^{ts};R23E10>VGlut^{RNAi2574GD} vs. tub-GAL80^{ts};R23E10<br>18 °C: tub-GAL80^{ts};R23E10>VGlut^{RNAi2574GD} vs. VGlut^{RNAi2574GD}<br>31 °C: tub-GAL80^{ts};R23E10>VGlut^{RNAi2574GD} vs. tub-GAL80^{ts};R23E10<br>31 °C: tub-GAL80^{ts};R23E10>VGlut^{RNAi2574GD} vs. VGlut^{RNAi2574GD}	 $F_{1,277}$ = 7.843<br>$F_{2,277}$ = 7.238<br>$F_{2,277}$ = 3.230<br>t_{277} = 0.8138<br>t_{277} = 0.1787<br>t_{277} = 4.197<br>t_{277} = 1.282	 0.0055<br>0.0009<br>0.0411<br>0.6595<br>0.8583<br><0.0001<br>0.2008
**8e, Bout number**	Two-way ANOVA<br>Temperature<br>Genotype<br>Temperature x genotype		 $F_{1,277}$ = 5.268<br>$F_{2,277}$ = 26.52<br>$F_{2,277}$ = 2.025	 0.0225<br><0.0001<br>0.1339
**8e, P(Wake)**	Two-way ANOVA<br>Temperature<br>Genotype<br>Temperature x genotype<br>Holm-Šidák test<br>Holm-Šidák test<br>Holm-Šidák test<br>Holm-Šidák test	 <br><br><br>18 °C: tub-GAL80^{ts};R23E10>VGlut^{RNAi2574GD} vs. tub-GAL80^{ts};R23E10<br>18 °C: tub-GAL80^{ts};R23E10>VGlut^{RNAi2574GD} vs. VGlut^{RNAi2574GD}<br>31 °C: tub-GAL80^{ts};R23E10>VGlut^{RNAi2574GD} vs. tub-GAL80^{ts};R23E10<br>31 °C: tub-GAL80^{ts};R23E10>VGlut^{RNAi2574GD} vs. VGlut^{RNAi2574GD}	 $F_{1,277}$ = 33.13<br>$F_{2,277}$ = 80.00<br>$F_{2,277}$ = 4.270<br>t_{277} = 3.711<br>t_{277} = 0.8741<br>t_{277} = 6.698<br>t_{277} = 3.183	 <0.0001<br><0.0001<br>0.0149<br>0.0005<br>0.3828<br><0.0001<br>0.0016
**8e, P(Doze)**	Two-way ANOVA<br>Temperature<br>Genotype<br>Temperature x genotype		 $F_{1,277}$ = 15.59<br>$F_{2,277}$ = 4.872<br>$F_{2,277}$ = 1.886	 <0.0001<br>0.0083<br>0.1537
**9a (top)**	Kruskal-Wallis ANOVA<br>Genotype<br>Dunn's test<br>Dunn's test<br>Dunn's test<br>Dunn's test	 <br>R23E10 ∩ VGlut-GAL4>GFP;RFP vs. R23E10 ∩ VGlut-GAL4>GFP;VGlut$^{RNAi\ 104324KK}$<br>R23E10 ∩ VGlut-GAL4>GFP;RFP vs. R23E10 ∩ VGlut-GAL4>GFP;VGlut$^{RNAi\ TRiP\ \#27538}$<br>R23E10 ∩ VGlut-GAL4>GFP;RFP vs. R23E10 ∩ VGlut-GAL4>GFP;VGlut$^{RNAi\ 2574GD}$<br>R23E10 ∩ VGlut-GAL4>GFP;RFP vs. R23E10 ∩ VGlut-GAL4>GFP;VGlut$^{RNAi\ TRiP\ \#40927}$	 H_5 = 4.246<br>Z = 1.380<br>Z = 1.127<br>Z = 0.9094<br>Z = 0.0238	 0.3737<br>0.6698<br>>0.9999<br>>0.9999<br>>0.9999
**9a (bottom)**	Kruskal-Wallis ANOVA			

	Genotype		$H_5 = 9.870$	0.0427
	Dunn's test	*R23E10 ∩ VGlut-GAL4>GFP;RFP* vs. *R23E10 ∩ VGlut-GAL4>GFP;VGlut*$^{RNAi\ 104324KK}$	$Z = 0.4778$	>0.9999
	Dunn's test	*R23E10 ∩ VGlut-GAL4>GFP;RFP* vs. *R23E10 ∩ VGlut-GAL4>GFP;VGlut*$^{RNAi\ TRiP\ \#27538}$	$Z = 1.885$	0.2376
	Dunn's test	*R23E10 ∩ VGlut-GAL4>GFP;RFP* vs. *R23E10 ∩ VGlut-GAL4>GFP;VGlut*$^{RNAi\ 2574GD}$	$Z = 1.690$	0.3643
	Dunn's test	*R23E10 ∩ VGlut-GAL4>GFP;RFP* vs. *R23E10 ∩ VGlut-GAL4>GFP;VGlut*$^{RNAi\ TRiP\ \#40927}$	$Z = 2.428$	0.0608
**9b (top)**	Kruskal-Wallis ANOVA			
	Genotype		$H_5 = 61.29$	<0.0001
	Dunn's test	*tsh-GAL80;R23E10>VGlutRNAi*^{104324KK} vs. *tsh-GAL80;R23E10*	$Z = 5.188$	<0.0001
	Dunn's test	*tsh-GAL80;R23E10>VGlutRNAi*^{104324KK} vs. *VGlutRNAi*^{104324KK}	$Z = 2.851$	0.0174
	Dunn's test	*tsh-GAL80;R23E10>VGlut*$^{RNAi\ 2574GD}$ vs. *tsh-GAL80;R23E10*	$Z = 6.763$	<0.0001
	Dunn's test	*tsh-GAL80;R23E10>VGlut*$^{RNAi\ 2574GD}$ vs. *VGlut*$^{RNAi\ 2574GD}$	$Z = 5.014$	<0.0001
**9b (bottom)**	Kruskal-Wallis ANOVA			
	Genotype		$H_5 = 79.74$	<0.0001
	Dunn's test	*tsh-GAL80;R23E10>VGlutRNAi*^{104324KK} vs. *tsh-GAL80;R23E10*	$Z = 0.1020$	>0.9999
	Dunn's test	*tsh-GAL80;R23E10>VGlutRNAi*^{104324KK} vs. *VGlutRNAi*^{104324KK}	$Z = 1.464$	0.5732
	Dunn's test	*tsh-GAL80;R23E10>VGlut*$^{RNAi\ 2574GD}$ vs. *tsh-GAL80;R23E10*	$Z = 4.132$	0.0001
	Dunn's test	*tsh-GAL80;R23E10>VGlut*$^{RNAi\ 2574GD}$ vs. *VGlut*$^{RNAi\ 2574GD}$	$Z = 7.578$	<0.0001
**9c (top)**	Two-way ANOVA			
	Temperature		$F_{1,277} = 92.26$	<0.0001
	Genotype		$F_{2,277} = 34.14$	<0.0001
	Temperature x genotype		$F_{2,277} = 5.777$	0.0035
	Holm-Šidák test	18 °C: *tub-GAL80*^{ts}*;R23E10>VGlut*^{RNAi2574GD} vs. *tub-GAL80*^{ts}*;R23E10*	$t_{277} = 2.663$	0.0164
	Holm-Šidák test	18 °C: *tub-GAL80*^{ts}*;R23E10>VGlut*^{RNAi2574GD} vs. *VGlut*^{RNAi2574GD}	$t_{277} = 1.834$	0.0677
	Holm-Šidák test	31 °C: *tub-GAL80*^{ts}*;R23E10>VGlut*^{RNAi2574GD} vs. *tub-GAL80*^{ts}*;R23E10*	$t_{277} = 7.003$	<0.0001
	Holm-Šidák test	31 °C: *tub-GAL80*^{ts}*;R23E10>VGlut*^{RNAi2574GD} vs. *VGlut*^{RNAi2574GD}	$t_{277} = 2.378$	0.0181
**9c (bottom)**	Two-way ANOVA			
	Temperature		$F_{1,277} = 9.023$	0.0029
	Genotype		$F_{2,277} = 0.1216$	0.8855
	Temperature x genotype		$F_{2,277} = 0.9717$	0.3797
**9d**	Kruskal-Wallis ANOVA			
	Genotype		$H_3 = 15.07$	0.0005
	Dunn's test	*R23E10>GCaMP* vs. *R23E10>GCaMP;VGlut*^{RNAi2574GD}	$Z = 3.590$	0.0007
	Dunn's test	*R23E10>GCaMP* vs. *R23E10>GCaMP,VGlutRNAi*^{104324KK}	$Z = 2.744$	0.0121
**10a**	Two-sided unpaired *t-test*	Rested vs. sleep-deprived	$t_{36} = 2.364$	0.0236

Supplementary Table 4. *Drosophila* strains.

Name	Genotype	Source or Identifier	Reference
R23E10	*w[1118]; P{y[+t7.7] w[+mC]=GMR23E10-GAL4}attP2*	BDSC_49032	64
R23E10-LexA	*w[1118]; P{y[+t7.7] w[+mC]=GMR23E10-lexA}attP40*	BDSC_52693	65
VGlut-3xGAL80	*y[1] w[*]; Mi{Trojan-3XGAL80.2}VGlut[MI04979-T3XG80.2]*	BDSC_60316	66
teashirt-GAL80	*w[*]; P{y[+t*]=GAL80}tsh[md621-GAL80]/CyO; TM2/TM6B*	BDSC_605556	67
tub-Gal80^{ts}	*w[*]; P{w[+mC]=tubP-GAL80[ts]}20; TM2/TM6B, Tb[1]*	BDSC_7019	68
R23E10-DBD	*w[1118]; P{y[+t7.7] w[+mC]=R23E10-GAL4.DBD}attP2/TM3, Sb[1]*	BDSC_69269	69
VGlut-AD	*w[*]; Mi{Trojan-p65AD.2}VGlut[MI04979-Tp65AD.2]/CyO*	BDSC_82986	70
R84C10-AD	*w[1118]; P{y[+t7.7] w[+mC]=R84C10-p65.AD}attP40; MKRS/TM6B, Tb[1]*	BDSC_70833	69
Gad1-AD	*y[1] w[*]; Mi{Trojan-p65AD.2}Gad1[MI09277-Tp65AD.2]/TM6B, Tb[1]*	BDSC_60322	66
R69F08	*w[1118]; P{y[+t7.7] w[+mC]=GMR69F08-GAL4}attP2*	BDSC_39499	42, 64
6xEGFP	*20XUAS-6XGFP (VK00018*)	Stowers lab (Montana State University)	71
mCD8::GFP (II)	*y[1] w[*]; P{w[+mC]=UAS-mCD8::GFP.L}LL5, P{UAS-mCD8::GFP.L}2*	BDSC_5137	72
mCD8::GFP (III)	*y[1] w[*]; betaTub60D[Pin-Yt]/CyO; P{w[+mC]=UAS-mCD8::GFP.L}LL6*	BDSC_5130	72
LexAop-GFP	*w[*]; P{w[+mC]=lexAop-rCD2-GFP}2*	BDSC_66687	73
CD4:tdTomato	*y[1] w[*]; P{w[+mC]=UAS-CD4-tdTom}7M1*	BDSC_35841	74
myr::RFP	*w[1118]; P{w[+mC]=UAS-myr-mRFP}2/TM6B, Tb[1]*	BDSC_7119	
STaR	*LexAOP-CD8::GFP; UAS-flp, PBac{y[+mDint2]w[+mc] = brp(FRT.Stop)V5-2A-LexA-VP16}*	Zipursky lab (UCLA)	46
GCaMP6f (or GCaMP)	*w[1118]; P{y[+t7.7] w[+mC]=20XUAS-IVS-GCaMP6f}attP40*	BDSC_42747	76
iGluSnFR(A184V)	*w[1118]; P{y[+t7.7] w[+mC]=20XUAS-iGluSnFR.A184V}attP*	BDSC_59611	77
iAChSnFr	*w[1118]; PBac{y[+mDint2] w[+mC]=20XUAS-iAChSnFR}VK00005*	BDSC_90308	78
GRAB$_{ACh}$	*w[*]; P{w[+mC]=UAS-GRAB(ACh2.0)}2/In(2LR)Gla, wg[Gla-1] PPO1[Bc]*	BDSC_80046	79
GRAB(DA2m)	*w[*]; P{y[+t7.7] w[+mC]=10XUAS-GRAB(DA2m)}attP40/CyO*	BDSC_90878	80
CsChrimson:: tdTomato	*20XUAS-IVS-CsChrimson::tdTomato (VK00005*)	Jayaraman lab (Janelia Research Campus)	82
UAS-TrpA1	*w[*]; P{y[+t7.7] w[+mC]=UAS-TrpA1(B).K}attP16*	BDSC_26263	63
hsFLP	*P{ry[+t7.2]=hsFLP}1, y[1] w[1118]; Dr[1]/TM3, Sb[1]*	BDSC_26902	61
Mutually exclusive CsChrimson/GFP	*10x-UAS-FRT > -IVS-mCD8::GFP-STOP-FRT > -IVS-CsChrimson::tdTomato (attp40)*	Miesenböck lab (University of Oxford)	24
Mutually exclusive CsChrimson/GCaMP	*10x-UAS-FRT > -IVS-GCaMP6f-STOP-FRT > -IVS-CsChrimson::tdTomato (attp40)*	Miesenböck lab (University of Oxford)	This study
FLP	*y[1] w[*]; P{w[+mC]=UAS-FLP.D}JD1*	BDSC_4539	83
Frt-STOP-Frt-EGFP::VGlut	*Frt-STOP-Frt-EGFP::VGlut (II)*	Griffith lab (Brandeis University)	23
Frt-STOP-Frt-EGFP::VGlut	*Frt-STOP-Frt-EGFP::VGlut (II)*	Griffith lab (Brandeis University)	23
EKO	*y[1] w[*]; P{w[+mC]=UAS-EKO[+]}222a P{w[+mC]=UAS-EKO[+]}222b*	BDSC_40974	84
Kir2.1	*UAS-Kir2.1/CyO (attP40)*	Miesenböck lab (University of Oxford)	43
VGlut $^{RNAi\ TRiP\ 104324KK}$	*P{KK107498}VIE-260B*	VDRC_104324	86
VGlut $^{RNAi\ 2574GD}$	*w1118; P{GD834}v2574/TM3*	VDRC_2574	86
VGlut $^{RNAi\ \#27538}$	*y[1] v[1]; P{y[+t7.7] v[+t1.8]=TRiP.JF02689}attP2*	BDSC_27538	87
VGlut $^{RNAi\ TRiP\ \#40927}$	*y[1] sc[*] v[1] sev[21]; P{y[+t7.7] v[+t1.8]=TRiP.HMS02175}attP40*	BDSC_40927	87
