# Supplementary material for: A half-center oscillator regulates sleep pressure
Source: Nat Neurosci. 2026 Oct 15;29(11):2798–809. doi: 10.1038/s41593-026-02431-9 (PMC13724273; doi:10.1038/s41593-026-02431-9)
Supplement: Supplementary file 2 [file 41593_2026_2431_MOESM2_ESM.pdf]

nature portfolio

Corresponding author(s): Gero Miesenböck

Last updated by author(s): Jul 7, 2026

# Reporting Summary

Nature Portfolio wishes to improve the reproducibility of the work that we publish. This form provides structure for consistency and transparency in reporting. For further information on Nature Portfolio policies, see our Editorial Policies and the Editorial Policy Checklist.

## Statistics

For all statistical analyses, confirm that the following items are present in the figure legend, table legend, main text, or Methods section.

| n/a | Confirmed | |
|---|---|---|
| ☐ | ☒ | The exact sample size (*n*) for each experimental group/condition, given as a discrete number and unit of measurement |
| ☐ | ☒ | A statement on whether measurements were taken from distinct samples or whether the same sample was measured repeatedly |
| ☐ | ☒ | The statistical test(s) used AND whether they are one- or two-sided<br>*Only common tests should be described solely by name; describe more complex techniques in the Methods section.* |
| ☒ | ☐ | A description of all covariates tested |
| ☐ | ☒ | A description of any assumptions or corrections, such as tests of normality and adjustment for multiple comparisons |
| ☐ | ☒ | A full description of the statistical parameters including central tendency (e.g. means) or other basic estimates (e.g. regression coefficient) AND variation (e.g. standard deviation) or associated estimates of uncertainty (e.g. confidence intervals) |
| ☐ | ☒ | For null hypothesis testing, the test statistic (e.g. *F*, *t*, *r*) with confidence intervals, effect sizes, degrees of freedom and *P* value noted<br>*Give P values as exact values whenever suitable.* |
| ☒ | ☐ | For Bayesian analysis, information on the choice of priors and Markov chain Monte Carlo settings |
| ☒ | ☐ | For hierarchical and complex designs, identification of the appropriate level for tests and full reporting of outcomes |
| ☐ | ☒ | Estimates of effect sizes (e.g. Cohen's *d*, Pearson's *r*), indicating how they were calculated |

*Our web collection on statistics for biologists contains articles on many of the points above.*

## Software and code

Policy information about availability of computer code

| | |
|---|---|
| Data collection | Two-photon imaging data were collected using ScanImage 5.4.0, running on MATLAB2015a.<br>Confocal imaging data were collected using the LEICA LAS AF 2.7.3.9723.<br>Sleep behavior data were collected using the DAM system (Trikinetics) or custom code in LabVIEW.<br>Electrophysiological data were collected using custom protocols in pCLAMP 10.5 (Molecular Devices) and Signal (Cambridge Electronic Design Ltd.). |
| Data analysis | Confocal imaging data were analyzed in Fiji 2.14.0/1.54f.<br>Two-photon imaging data were analyzed in Fiji and MATLAB2024a with custom code, available on request.<br>Electrophysiological data were analyzed using version 3.0c of the NeuroMatic package in Igor Pro 8.04 (WaveMetrics) and MATLAB2024a.<br>Sleep behavior data were analyzed with the Sleep and Circadian Analysis MATLAB Program (SCAMP v3) or a custom LabVIEW script for video tracking.<br>Connectomic data were analyzed in MATLAB2024a. |

For manuscripts utilizing custom algorithms or software that are central to the research but not yet described in published literature, software must be made available to editors and reviewers. We strongly encourage code deposition in a community repository (e.g. GitHub). See the Nature Portfolio guidelines for submitting code & software for further information.

# Data

Policy information about availability of data

All manuscripts must include a data availability statement. This statement should provide the following information, where applicable:

- Accession codes, unique identifiers, or web links for publicly available datasets
- A description of any restrictions on data availability
- For clinical datasets or third party data, please ensure that the statement adheres to our policy

The Drosophila hemibrain (v1.2.1) and FlyWire (FAFB v783) connectome datasets used in Extended Data Fig. 3 are publicly available at https://neuprint.janelia.org and https://codex.flywire.ai, respectively.
All source data are provided with this manuscript and have been deposited at https://github.com/CNCBMiesenboeck/dFBNOscillator.

# Research involving human participants, their data, or biological material

Policy information about studies with human participants or human data. See also policy information about sex, gender (identity/presentation), and sexual orientation and race, ethnicity and racism.

| | |
|---|---|
| Reporting on sex and gender | n/a |
| Reporting on race, ethnicity, or other socially relevant groupings | n/a |
| Population characteristics | n/a |
| Recruitment | n/a |
| Ethics oversight | n/a |

Note that full information on the approval of the study protocol must also be provided in the manuscript.

# Field-specific reporting

Please select the one below that is the best fit for your research. If you are not sure, read the appropriate sections before making your selection.

☒ Life sciences ☐ Behavioural & social sciences ☐ Ecological, evolutionary & environmental sciences

For a reference copy of the document with all sections, see nature.com/documents/nr-reporting-summary-flat.pdf

# Life sciences study design

All studies must disclose on these points even when the disclosure is negative.

| | |
|---|---|
| Sample size | Sample sizes are provided in figure and extended data figure legends. In behavioral experiments sample sizes (typically n=32 flies per genotype) were chosen to detect 2-h differences in daily sleep with a power of 0.9. |
| Data exclusions | Only healthy flies in which the adhesive glue effectively suppressed brain movements were used for two-photon imaging.<br>ROIs with low signal-to-noise ratio and redundant ROIs (as detailed in Methods) were excluded in an automated and blinded manner.<br>Immobile flies (< 2 beam breaks per 24 h in the Trikinetics Drosophila Activity Monitor system) were excluded from the analysis in standard sleep assays.<br>Only flies losing >95% of baseline sleep (mechanical deprivation) or >30% of baseline sleep (visual deprivation) were included in measurements of sleep rebound.<br>Only anatomically intact specimens from live flies (at the point of dissection) were used for neuroanatomy or STaR.<br>Only dFBNs firing more than one action potential in response to depolarizing current injections, with resting potentials <–30 mV and series resistances <50 MΩ, were characterized. |
| Replication | All behavioral experiments were run at least three times, on different days and with different batches of flies. |
| Randomization | Female flies of a given genotype, as indicated in Methods and figure legends, were randomly selected for analysis. Controls and experimental groups were always tested in parallel and in randomized order. For calcium imaging after sleep deprivation, siblings with imaging windows were assigned randomly to experimental and control groups. |
| Blinding | During the selection of ROIs and background regions, experimenters were blind to sleep history and/or zeitgeber time (calcium imaging in Fig. 1a, 2, 6; Extended Data Fig. 1h–j, Extended Data Fig. 2, STaR in Fig. 8a and Extended Data Fig. 10a; iGluSnFR imaging in Fig. 8b), the retinal content of food (calcium imaging of FLP-constructs in Fig. 3f), RNAi-mediated knockdown of VGlut (calcium imaging in Fig. 5e and Extended Data Fig. 9d,e), and the expression of CsChrimson (dopamine imaging in Fig. 7h), but not otherwise. |

# Reporting for specific materials, systems and methods

We require information from authors about some types of materials, experimental systems and methods used in many studies. Here, indicate whether each material, system or method listed is relevant to your study. If you are not sure if a list item applies to your research, read the appropriate section before selecting a response.

## Materials & experimental systems

| n/a | Involved in the study |
|---|---|
| ☐ | ☒ Antibodies |
| ☒ | ☐ Eukaryotic cell lines |
| ☒ | ☐ Palaeontology and archaeology |
| ☐ | ☒ Animals and other organisms |
| ☒ | ☐ Clinical data |
| ☒ | ☐ Dual use research of concern |
| ☒ | ☐ Plants |

## Methods

| n/a | Involved in the study |
|---|---|
| ☒ | ☐ ChIP-seq |
| ☒ | ☐ Flow cytometry |
| ☒ | ☐ MRI-based neuroimaging |

## Antibodies

| | |
|---|---|
| Antibodies used | Primary: nc82 anti-BRP (1:10, Developmental Studies Hybridoma Bank, AB_2314866), mouse anti-V5 (1:400, ThermoFisher, AB_2556564), chicken anti-GFP (1:1000 or 1:500 for STaR, AbCam, AB_10561923), and mouse anti-GFP (1:50, ThermoFisher, for GCaMP, AB_221568).<br>Secondary: goat anti-Mouse Alexa Fluor 488 (1:1000, ThermoFisher, AB_141607), goat anti-Chicken Alexa Fluor 488 (1:1000 or 1:500 for STaR, ThermoFisher, AB_2534096), and goat anti-Mouse Alexa Fluor 633 (1:500, ThermoFisher, AB_2535719) |
| Validation | AB_2314866 was validated in fly brains by immunofluorescence (IF) microscopy, and has been cited in 1046 references as of February 2024.<br>AB_2556564 was validated by the manufacturer in mammalian cells transfected with V5-tagged vectors in Western blots (WB) and by IF.<br>AB_10561923 was validated in ELISA, immunocytochemistry (ICC), and WB.<br>AB_221568 was validated by the manufacturer in ICC/IF in mammalian cells transfected with GFP-tagged constructs.<br>AB_141607 was validated by the manufacturer in ICC/IF in mammalian cells stained with primary antibody.<br>AB_2534096 was validated by the manufacturer in ICC/IF in tissued stained with primary antibody.<br>AB_2535719 was validated by the manufacturer in mammalian cells stained with primary antibody. |

## Animals and other research organisms

Policy information about studies involving animals; ARRIVE guidelines recommended for reporting animal research, and Sex and Gender in Research

| | |
|---|---|
| Laboratory animals | Transgenic Drosophila melanogaster strains; genotypes indicated in Methods. |
| Wild animals | n/a |
| Reporting on sex | Male and female flies were used to generate the desired genotypes. Female flies were used in all experiments because of their larger body size. |
| Field-collected samples | n/a |
| Ethics oversight | No ethical approval was required for research on Drosophila melanogaster. |

Note that full information on the approval of the study protocol must also be provided in the manuscript.

## Plants

Seed stocks

*Report on the source of all seed stocks or other plant material used. If applicable, state the seed stock centre and catalogue number. If plant specimens were collected from the field, describe the collection location, date and sampling procedures.*

Novel plant genotypes

*Describe the methods by which all novel plant genotypes were produced. This includes those generated by transgenic approaches, gene editing, chemical/radiation-based mutagenesis and hybridization. For transgenic lines, describe the transformation method, the number of independent lines analyzed and the generation upon which experiments were performed. For gene-edited lines, describe the editor used, the endogenous sequence targeted for editing, the targeting guide RNA sequence (if applicable) and how the editor was applied.*

Authentication

*Describe any authentication procedures for each seed stock used or novel genotype generated. Describe any experiments used to assess the effect of a mutation and, where applicable, how potential secondary effects (e.g. second site T-DNA insertions, mosiacism, off-target gene editing) were examined.*
